# Antidepressants for smoking cessation

**DOI:** 10.1002/14651858.CD000031.pub6

**Published:** 2023-05-24

**Authors:** Anisa Hajizadeh, Seth Howes, Annika Theodoulou, Elias Klemperer, Jamie Hartmann-Boyce, Jonathan Livingstone-Banks, Nicola Lindson

**Affiliations:** Nuffield Department of Primary Care Health SciencesUniversity of OxfordOxfordUK; Departments of Psychological Sciences & PsychiatryUniversity of VermontBurlingtonVTUSA

**Keywords:** Adolescent, Adult, Child, Humans, Young Adult, Antidepressive Agents, Antidepressive Agents/adverse effects, Bupropion, Bupropion/adverse effects, Nicotinic Agonists, Nicotinic Agonists/adverse effects, Nortriptyline, Nortriptyline/adverse effects, Smoking Cessation, Smoking Cessation/methods, Varenicline, Varenicline/adverse effects

## Abstract

**Background:**

The pharmacological profiles and mechanisms of antidepressants are varied. However, there are common reasons why they might help people to stop smoking tobacco: nicotine withdrawal can produce short‐term low mood that antidepressants may relieve; and some antidepressants may have a specific effect on neural pathways or receptors that underlie nicotine addiction.

**Objectives:**

To assess the evidence for the efficacy, harms, and tolerability of medications with antidepressant properties in assisting long‐term tobacco smoking cessation in people who smoke cigarettes.

**Search methods:**

We searched the Cochrane Tobacco Addiction Group Specialised Register, most recently on 29 April 2022.

**Selection criteria:**

We included randomised controlled trials (RCTs) in people who smoked, comparing antidepressant medications with placebo or no pharmacological treatment, an alternative pharmacotherapy, or the same medication used differently. We excluded trials with fewer than six months of follow‐up from efficacy analyses. We included trials with any follow‐up length for our analyses of harms.

**Data collection and analysis:**

We extracted data and assessed risk of bias using standard Cochrane methods.

Our primary outcome measure was smoking cessation after at least six months' follow‐up. We used the most rigorous definition of abstinence available in each trial, and biochemically validated rates if available. Our secondary outcomes were harms and tolerance outcomes, including adverse events (AEs), serious adverse events (SAEs), psychiatric AEs, seizures, overdoses, suicide attempts, death by suicide, all‐cause mortality, and trial dropouts due to treatment. We carried out meta‐analyses where appropriate.

**Main results:**

We included a total of 124 studies (48,832 participants) in this review, with 10 new studies added to this update version. Most studies recruited adults from the community or from smoking cessation clinics; four studies focused on adolescents (with participants between 12 and 21 years old). We judged 34 studies to be at high risk of bias; however, restricting analyses only to studies at low or unclear risk of bias did not change clinical interpretation of the results.

There was high‐certainty evidence that bupropion increased smoking cessation rates when compared to placebo or no pharmacological treatment (RR 1.60, 95% CI 1.49 to 1.72; I^2^ = 16%; 50 studies, 18,577 participants). There was moderate‐certainty evidence that a combination of bupropion and varenicline may have resulted in superior quit rates to varenicline alone (RR 1.21, 95% CI 0.95 to 1.55; I^2^ = 15%; 3 studies, 1057 participants). However, there was insufficient evidence to establish whether a combination of bupropion and nicotine replacement therapy (NRT) resulted in superior quit rates to NRT alone (RR 1.17, 95% CI 0.95 to 1.44; I^2^ = 43%; 15 studies, 4117 participants; low‐certainty evidence).

There was moderate‐certainty evidence that participants taking bupropion were more likely to report SAEs than those taking placebo or no pharmacological treatment. However, results were imprecise and the CI also encompassed no difference (RR 1.16, 95% CI 0.90 to 1.48; I^2^ = 0%; 23 studies, 10,958 participants). Results were also imprecise when comparing SAEs between people randomised to a combination of bupropion and NRT versus NRT alone (RR 1.52, 95% CI 0.26 to 8.89; I^2^ = 0%; 4 studies, 657 participants) and randomised to bupropion plus varenicline versus varenicline alone (RR 1.23, 95% CI 0.63 to 2.42; I^2^ = 0%; 5 studies, 1268 participants). In both cases, we judged evidence to be of low certainty.

There was high‐certainty evidence that bupropion resulted in more trial dropouts due to AEs than placebo or no pharmacological treatment (RR 1.44, 95% CI 1.27 to 1.65; I^2^ = 2%; 25 studies, 12,346 participants). However, there was insufficient evidence that bupropion combined with NRT versus NRT alone (RR 1.67, 95% CI 0.95 to 2.92; I^2^ = 0%; 3 studies, 737 participants) or bupropion combined with varenicline versus varenicline alone (RR 0.80, 95% CI 0.45 to 1.45; I^2^ = 0%; 4 studies, 1230 participants) had an impact on the number of dropouts due to treatment. In both cases, imprecision was substantial (we judged the evidence to be of low certainty for both comparisons).

Bupropion resulted in inferior smoking cessation rates to varenicline (RR 0.73, 95% CI 0.67 to 0.80; I^2^ = 0%; 9 studies, 7564 participants), and to combination NRT (RR 0.74, 95% CI 0.55 to 0.98; I^2^ = 0%; 2 studies; 720 participants). However, there was no clear evidence of a difference in efficacy between bupropion and single‐form NRT (RR 1.03, 95% CI 0.93 to 1.13; I^2^ = 0%; 10 studies, 7613 participants). We also found evidence that nortriptyline aided smoking cessation when compared with placebo (RR 2.03, 95% CI 1.48 to 2.78; I^2^ = 16%; 6 studies, 975 participants), and some evidence that bupropion resulted in superior quit rates to nortriptyline (RR 1.30, 95% CI 0.93 to 1.82; I^2^ = 0%; 3 studies, 417 participants), although this result was subject to imprecision.

Findings were sparse and inconsistent as to whether antidepressants, primarily bupropion and nortriptyline, had a particular benefit for people with current or previous depression.

**Authors' conclusions:**

There is high‐certainty evidence that bupropion can aid long‐term smoking cessation. However, bupropion may increase SAEs (moderate‐certainty evidence when compared to placebo/no pharmacological treatment). There is high‐certainty evidence that people taking bupropion are more likely to discontinue treatment compared with people receiving placebo or no pharmacological treatment. Nortriptyline also appears to have a beneficial effect on smoking quit rates relative to placebo, although bupropion may be more effective. Evidence also suggests that bupropion may be as successful as single‐form NRT in helping people to quit smoking, but less effective than combination NRT and varenicline. In most cases, a paucity of data made it difficult to draw conclusions regarding harms and tolerability.

Further studies investigating the efficacy of bupropion versus placebo are unlikely to change our interpretation of the effect, providing no clear justification for pursuing bupropion for smoking cessation over other licensed smoking cessation treatments; namely, NRT and varenicline. However, it is important that future studies of antidepressants for smoking cessation measure and report on harms and tolerability.

## Plain language summary

**Do medicines for depression help people to quit smoking?**

**What are antidepressants?**

Antidepressants are medicines and supplements used to treat depression. Some have also been tested to see whether they can help people to stop smoking. Two of these treatments – bupropion (sometimes called Zyban) and nortriptyline – are sometimes given to help people quit smoking.

**Why we did this Cochrane Review**

Smoking tobacco is extremely bad for people’s health. For people who smoke, quitting is the best thing they can do to improve their health. Many people find it difficult to quit smoking. We wanted to find out whether using antidepressants helps people to stop smoking (for six months or longer), and what potential harms might come from using these medicines.

**We were interested in finding out:**

‐ how many people stopped smoking for at least six months; and

‐ how many people had unwanted effects.

**What did we do?**

We searched for studies that looked at the use of antidepressants to help people quit smoking.

We looked for randomised controlled trials, in which the treatments people received were decided at random. This type of study usually gives the most reliable evidence about the effects of treatment. We included studies of any length when looking at evidence of harms, but studies needed to be at least six months long when assessing whether people had managed to quit smoking.

**What we found**

This review includes 124 studies, including 48,832 participants, looking at how helpful and safe different antidepressants are when used to quit smoking. Most studies were conducted in adults who smoked tobacco, with and without a history of mental illness. Four studies recruited young people aged 12 to 21 years. Most participants were motivated to quit smoking.

**What are the results of our review?**

Compared to not using any medication, using the antidepressant bupropion makes it 49% to 72% more likely that a person will successfully stop smoking, which is equal to six to eight more people successfully quitting for six months or more for every one hundred people who try to quit. There is evidence that people who use the antidepressant nortriptyline to quit smoking also improve their chances of success (48% to 178% more likely).

Bupropion may increase serious unwanted effects (such as death, hospitalisation, or life‐threatening events). Unwanted effects may increase the chance that people stop using the medicine. There is not enough information to draw clear conclusions about the harms of nortriptyline for stopping smoking.

The evidence suggests that taking bupropion at the same time as other stop‐smoking medicines – varenicline (a drug sometimes known as Champix or Chantix which is not an antidepressant) and combination nicotine replacement therapy (a patch plus another form) – may make people more likely to quit smoking than if they use nicotine replacement therapy or varenicline on their own. However, further evidence may change our findings. The evidence does not suggest a benefit of using bupropion at the same time as a single form of nicotine replacement therapy; for example, a patch, gum, or lozenge alone. People may be more likely to quit smoking when using bupropion compared with nortriptyline, but more likely to quit when using varenicline than bupropion.

**How reliable are these results?**

There is high‐certainty evidence that bupropion helps people to quit smoking, meaning further research is very unlikely to change this conclusion. However, there is also high‐certainty evidence to suggest that people using bupropion are more likely to stop taking the medicine because of unpleasant effects than those taking a pill without medication (a placebo) or no medication. The certainty of the evidence was moderate or low for the other key questions we looked at. This means that the findings of those questions may change when more research is carried out. In most cases, this was because there were not enough studies or studies were too small.

**How up to date is this evidence?**

This review updates our previous review. The evidence is up to date to April 2022.

**Key messages**

‐ Bupropion can help people to quit smoking but may make people more likely to experience serious unwanted effects that could result in people stopping taking it or having to go to the hospital.

‐ Nortriptyline also appears to help people to quit smoking, but bupropion may be more effective.

‐ Bupropion may be as helpful as a single form of nicotine replacement therapy in helping people to quit smoking, but less so than combination nicotine replacement therapy (that is, a patch plus another form) and varenicline.

## Summary of findings

**Summary of findings 1:** Bupropion versus placebo/no pharmacological treatment for smoking cessation

**Bupropion versus placebo/no pharmacological treatment for smoking cessation**						
**Population:** people who smoke **Setting:** any; studies conducted in Asia, Australasia, Europe, USA **Intervention:** bupropion **Comparison:** placebo/no pharmacological treatment						
**Outcomes**	Anticipated absolute effects^*^ (95% CI)		**Relative effect (95% CI)**	**№ of participants (studies)**	**Certainty of the evidence (GRADE)**	**Comments**
	**Risk with placebo/no pharmacological treatment**	**Risk with bupropion**				
Smoking cessation (at least six months of follow‐up)	Study population		RR 1.60 (1.49 to 1.72)	18,577 (50 RCTs)	⊕⊕⊕⊕ High	
	12 per 100	19 per 100 (18 to 20)				
Serious adverse events	Study population		RR 1.16 (0.90 to 1.48)	10,958 (23 RCTs)	⊕⊕⊕⊝ Moderate^a,b^	Eight of the studies reported no SAEs across relevant study arms
	2 per 100	3 per 100 (2 to 3)				
Dropouts due to treatment	Study population		RR 1.44 (1.27 to 1.65)	12,346 (25 RCTs)	⊕⊕⊕⊕ High	
	6 per 100	9 per 100 (7 to 9)				
*The risk in the intervention group (and its 95% confidence interval) is based on the assumed risk in the comparison group and the **relative effect** of the intervention (and its 95% CI). **CI:** confidence interval; **NRT:** nicotine replacement therapy; **RCT**: randomised controlled trial; **RR:** risk ratio						
**GRADE Working Group grades of evidence** **High certainty**: we are very confident that the true effect lies close to that of the estimate of the effect. **Moderate certainty**: we are moderately confident in the effect estimate; the true effect is likely to be close to the estimate of the effect, but there is a possibility that it is substantially different. **Low certainty**: our confidence in the effect estimate is limited; the true effect may be substantially different from the estimate of the effect. **Very low certainty**: we have very little confidence in the effect estimate; the true effect is likely to be substantially different from the estimate of effect.						

^a^Downgraded one level due to imprecision. Confidence interval encompasses no difference as well as the potential for more serious adverse events when using bupropion. ^b^Not downgraded due to publication bias, despite some slight asymmetry, as would not expect the reported serious adverse event outcome to influence the likelihood of publication.

**Summary of findings 2:** Bupropion plus nicotine replacement therapy (NRT) versus NRT alone for smoking cessation

**Bupropion plus nicotine replacement therapy (NRT) versus NRT alone for smoking cessation**						
**Population:** people who smoke **Setting:** any; studies conducted in Asia/Africa, Europe, USA **Intervention:** bupropion plus NRT **Comparison:** NRT alone						
**Outcomes**	Anticipated absolute effects^*^ (95% CI)		**Relative effect (95% CI)**	**№ of participants (studies)**	**Certainty of the evidence (GRADE)**	**Comments**
	**Risk with NRT alone**	**Risk with bupropion plus NRT**				
Smoking cessation (at least six months of follow‐up)	Study population		RR 1.17 (0.95 to 1.44)	4117 (15 RCTs)	⊕⊕⊝⊝ Low^a,b^	
	17 per 100	20 per 100 (16 to 25)				
Serious adverse events	Study population		RR 1.52 (0.26 to 8.89)	657 (4 RCTs)	⊕⊕⊝⊝ Low^c^	Two of the studies reported no SAEs across relevant study arms
	1 per 100	1 per 100 (0 to 5)				
Dropouts due to treatment	Study population		RR 1.67 (0.95 to 2.92)	737 (3 RCTs)	⊕⊕⊝⊝ Low^d^	One of the studies reported no dropouts across relevant study arms
	5 per 100	8 per 100 (5 to 14)				
*The risk in the intervention group (and its 95% confidence interval) is based on the assumed risk in the comparison group and the **relative effect** of the intervention (and its 95% CI). **CI:** confidence interval; **NRT:** nicotine replacement therapy; **RCT**: randomised controlled trial; **RR:** risk ratio						
**GRADE Working Group grades of evidence** **High certainty**: we are very confident that the true effect lies close to that of the estimate of the effect. **Moderate certainty**: we are moderately confident in the effect estimate; the true effect is likely to be close to the estimate of the effect, but there is a possibility that it is substantially different. **Low certainty**: our confidence in the effect estimate is limited; the true effect may be substantially different from the estimate of the effect. **Very low certainty**: we have very little confidence in the effect estimate; the true effect is likely to be substantially different from the estimate of effect.						

^a^Downgraded one level due to inconsistency. Unexplained statistical heterogeneity (I^2^ = 43%). ^b^Downgraded one level due to imprecision. The CI encompasses the potential for benefit as well as harm. ^c^Downgraded two levels due to imprecision. Low numbers of events and the CI encompasses the potential for benefit as well as harm.

**Summary of findings 3:** Bupropion plus varenicline versus varenicline alone for smoking cessation

**Bupropion plus varenicline versus varenicline alone for smoking cessation**						
**Population:** people who smoke **Setting:** any; studies conducted in USA **Intervention:** bupropion plus varenicline **Comparison:** varenicline alone						
**Outcomes**	Anticipated absolute effects^*^ (95% CI)		**Relative effect (95% CI)**	**№ of participants (studies)**	**Certainty of the evidence (GRADE)**	**Comments**
	**Risk with varenicline alone**	**Risk with bupropion plus varenicline**				
Smoking cessation (at least six months of follow‐up)	Study population		RR 1.21 (0.95 to 1.55)	1057 (3 RCTs)	⊕⊕⊕⊝ Moderate^a^	
	21 per 100	26 per 100 (20 to 33)				
Serious adverse events	Study population		RR 1.23 (0.63 to 2.42)	1268 (5 RCTs)	⊕⊕⊝⊝ Low^b^	One of the studies reported no SAEs across relevant study arms
	2 per 100	3 per 100 (1 to 6)				
Dropouts due to treatment	Study population		RR 0.80 (0.45 to 1.45)	1230 (4 RCTs)	⊕⊕⊝⊝ Low^b^	
	4 per 100	3 per 100 (2 to 6)				
*The risk in the intervention group (and its 95% confidence interval) is based on the assumed risk in the comparison group and the **relative effect** of the intervention (and its 95% CI). **CI:** confidence interval; **NRT:** nicotine replacement therapy; **RCT**: randomised controlled trial; **RR:** risk ratio						
**GRADE Working Group grades of evidence** **High certainty**: we are very confident that the true effect lies close to that of the estimate of the effect. **Moderate certainty**: we are moderately confident in the effect estimate; the true effect is likely to be close to the estimate of the effect, but there is a possibility that it is substantially different. **Low certainty**: our confidence in the effect estimate is limited; the true effect may be substantially different from the estimate of the effect. **Very low certainty**: we have very little confidence in the effect estimate; the true effect is likely to be substantially different from the estimate of effect.						

^a^Downgraded one level due to imprecision. The CI encompasses no difference as well as clinically significant benefit. ^b^Downgraded two levels due to imprecision. Low numbers of events and the CI encompasses the potential for benefit as well as harm.

## Background

### Description of the condition

Tobacco use is one of the leading causes of preventable illness and death worldwide, accounting for over seven million deaths annually (GBD 2019 TC). Extrapolation based on current smoking trends suggests that, without widespread quitting, approximately 4050 million tobacco‐related deaths will occur between 2000 and 2050, mostly amongst current smokers (Jha 2011). Most smokers would like to stop (CDC 2017); however, quitting tobacco use is difficult. This is because users develop both a psychological and physiological dependence on smoking. The physiological dependence is caused by a component of tobacco, called nicotine (McNeill 2017).

### Description of the intervention

Whilst antidepressant medications are primarily used for the treatment of depression and disorders of negative affect, they have also been used to help individuals stop smoking. They offer an alternative to other first‐line smoking cessation pharmacotherapies (first treatments provided for tobacco addiction), such as nicotine replacement therapy (NRT), and nicotine agonists, such as varenicline. 

The following medications and substances, regarded as having antidepressant properties, have been investigated for their effect on smoking cessation in at least one study.

- Tricyclic antidepressants (TCAs): doxepin, imipramine, and nortriptyline
- Monoamine oxidase inhibitors (MAOIs): moclobemide, selegiline, lazabemide, and EVT302
- Selective serotonin reuptake inhibitors (SSRIs): fluoxetine, paroxetine, sertraline, citalopram, and zimelidine
- Atypical antidepressants: bupropion, tryptophan, and venlafaxine
- Extracts of St. John's wort (*Hypericum perforatum*)
- Dietary supplement: S‐adenosyl‐L‐methionine (SAMe)

Of the antidepressant medications indicated for smoking cessation, the most commonly used is bupropion. It has both dopaminergic and adrenergic actions, and appears to be an antagonist at the nicotinic acetylcholinergic receptor (Fryer 1999). It has been licensed as a prescription medicine for smoking cessation in many countries. The usual dose for smoking cessation is 150 mg once a day for three days, increasing to 150 mg twice a day continued for seven to 12 weeks. Quit attempts are generally initiated one week after starting pharmacotherapy.

Following bupropion, the second most commonly tested medication for smoking cessation is the TCA, nortriptyline. It enhances noradrenergic and serotonergic activity by blocking reuptake of these neurotransmitters (Benowitz 2000). It is licensed for smoking cessation in New Zealand. The recommended regimen is 10 to 28 days of titration before the quit attempt, followed by a 12‐week dose of 75 mg to 100 mg daily (Cahill 2013).

No other antidepressants are currently licensed for use as smoking cessation treatment, although others have been tested for possible use.

### How the intervention might work

Multiple observations have provided a rationale for studying the effects of antidepressant medications for smoking cessation: a history of depression is found more frequently amongst people who smoke than people who do not smoke; nicotine may have antidepressant effects; and antidepressants influence the neurotransmitters and receptors involved in nicotine addiction (Benowitz 2000; Kotlyar 2001). Although evidence suggests that cessation is likely to improve rather than worsen mood in the long‐term (Taylor 2021), smoking abstinence does cause short‐term withdrawal symptoms, including depressed mood and irritability, which may prompt relapse to smoking (Hughes 2007).

The diverse pharmacological targets of antidepressants means their mechanisms of action are varied. Evidence suggests bupropion may aid smoking cessation by blocking nicotine effects, relieving withdrawal (Cryan 2003; West 2008), and reducing depressed mood (Lerman 2002). Monoamine oxidase‐A (MOA‐A) inhibitors may aid smoking cessation by substituting the ability of smoking to act as an MOA inhibitor (Lewis 2007). It has been hypothesised that SSRIs might be helpful because they increase serotonin, which is also associated with improving negative affect (Benowitz 2000). The mechanisms of other antidepressants for smoking cessation remain unstudied.

Although there is an evident relationship between alleviating negative affect and antidepressant pharmacology, it is unclear whether antidepressants work mostly due to reducing negative affect, reducing urges to smoke or withdrawal symptoms, or by acting as nicotine blockers.

### Why it is important to do this review

This is the fifth update of a review first published in 2003. It is a linked to a wider project updating all evidence on licenced pharmacotherapies and e‐cigarettes for smoking cessation (Lindson 2022). It was deemed appropriate to update this review as a useful output of this wider project for the following reasons. The ongoing impact of smoking on global morbidity and mortality necessitates effective and safe treatments to aid smoking cessation. Emerging evidence assessing antidepressants as smoking cessation treatments has the potential to change or strengthen many of our conclusions regarding the efficacy, harms, and tolerability of the antidepressants currently being used to help people quit smoking (bupropion and nortriptyline). Further evidence on harms may help to clarify the potential interaction between bupropion and seizures, as well as psychiatric adverse events. Multiple trials and observational studies have previously associated bupropion with increasing the risk of medically important adverse events, including seizures, anxiety, depression, and insomnia (Aubin 2012). New evidence may also help us to directly compare the harms and efficacy of antidepressants with other first‐line smoking cessation pharmacotherapies, providing a further aid to decision making when helping people to quit tobacco smoking.

## Objectives

To assess the evidence for the efficacy, harms, and tolerability of medications with antidepressant properties in assisting long‐term tobacco smoking cessation in people who smoke cigarettes.

## Methods

### Criteria for considering studies for this review

#### Types of studies

We included randomised controlled trials (RCTs) and cluster‐RCTs. We did not include cross‐over studies as this type of randomised study does not allow assessment of the medium‐ to long‐term effects of smoking cessation medications on abstinence.

#### Types of participants

We included people of any age who smoked tobacco, with or without a history of mental illness. We did not include pregnant women, as this specific subpopulation is covered in a separate Cochrane Review (Claire 2020).

#### Types of interventions

We included trials studying pharmacotherapies with antidepressant properties for smoking cessation. We included trials assessing different doses, durations, and schedules of antidepressants.

We excluded trials where an additional, uncontrolled, non‐antidepressant intervention component was used in only one of the trial arms. This is because the confounding effects of this intervention would have made it difficult to determine whether any change in outcome was related to the antidepressant or the confounding intervention component. Additionally, we excluded trials investigating antidepressant use for smoking harm reduction or relapse prevention, as they are covered elsewhere (Lindson‐Hawley 2016 and Livingstone‐Banks 2019, respectively).

##### Comparators

The following comparators were eligible for assessing harms, efficacy, and tolerability: placebo, no pharmacological treatment, alternative therapeutic control, or different dosages/treatment regimes of the same antidepressant.

#### Types of outcome measures

##### Primary outcomes

- Efficacy, measured as smoking cessation

For this outcome, we only included studies that set out to report smoking cessation rates at least six months after baseline, in line with the standard methods of Cochrane Tobacco Addiction. Where cessation was assessed at multiple intervals, we reported only the longest follow‐up data. Additionally, where studies assessed multiple definitions of abstinence, we reported the strictest of these definitions (e.g. continuous/prolonged abstinence over point prevalence abstinence). We also reported biochemical validation of abstinence over self‐reported abstinence (but it was not necessary for abstinence to have been biochemically validated for a study to be included).

##### Secondary outcomes

- Harms, measured as:
  - number of people experiencing adverse events (AEs) of any severity (e.g. abnormal test findings, clinically significant symptoms and signs, changes in physical examination findings, hypersensitivity, and progression or worsening of underlying disease);
  - number of people experiencing psychiatric AEs (e.g. adverse events relating to mental health);
  - number of people experiencing serious adverse events (SAEs); that is, events that result in death, are life‐threatening (immediate risk of death), require inpatient hospitalisation or prolongation of existing hospitalisation, result in persistent or significant disability or incapacity (e.g. seizures, overdoses, suicide attempts, death by suicide, all‐cause mortality).

We also recorded the following SAEs specifically, as these have previously been associated with the use of antidepressants for smoking cessation.

- Number of people experiencing seizures
- Number of people experiencing overdoses
- Number of people experiencing suicide attempts
- Number of people experiencing death by suicide
- Number of people experiencing all‐cause mortality
- Tolerability, measured as the number of participants who dropped out of the trial due to adverse events

For all harm and tolerability outcomes, we considered studies with follow‐up of any length.

### Search methods for identification of studies

#### Electronic searches

We identified studies from the Cochrane Tobacco Addiction Specialised Register. At the time of the updated search (29 April 2022), the Register included reports of trials indexed in: the Cochrane Central Register of Controlled Trials (CENTRAL), Issue 3, 2022; MEDLINE (via OVID) to update 2022 April 05; Embase (via OVID) to 2022 week 14; PsycINFO (via OVID) to update 2022 April 04 – all from inception. See the Cochrane Tobacco Addiction website for full search strategies and a list of other resources searched to populate the Register. We searched the Register for reports of studies evaluating bupropion, nortriptyline, or any other pharmacotherapy classified as having an antidepressant effect. Searches for the Register are not restricted by date, language, or format of publication. Search terms included relevant individual drug names or antidepressant* or antidepressive*. See Appendix 1 for the Register search strategy.

#### Searching other resources

Our searches of the Cochrane Tobacco Addiction Specialised Register also covered records in ClinicalTrials.gov (ClinicalTrials.gov) and the World Health Organization International Clinical Trials Registry Platform (ICTRP), as these are indexed in CENTRAL.

### Data collection and analysis

#### Selection of studies

For this update, two review authors (of NL, AT, EK, AH) independently screened titles and abstracts resulting from our searches for relevance, and obtained full‐text records of reports of possibly eligible studies. Two review authors (of NL, AT, EK, AH) then independently screened each full‐text record for eligibility. Any disagreements were resolved through discussion with a third review author. For conference abstracts or trial registry entries where the record contained insufficient evidence for us to determine the eligibility, we attempted to contact study investigators to obtain any additional data needed to make a final decision. We recorded all screening decisions made and presented the flow of studies and references through the reviewing process using a PRISMA flow diagram (Moher 2009).

#### Data extraction and management

Two review authors (for this update EK, AH) independently extracted the following study data and compared the findings. We resolved any discrepancies by mutual consent.

- Type of antidepressant
- Type of RCT
- Country and setting
- Recruitment method
- Participant demographics (i.e. average age, gender, average cigarettes per day, motivation to quit, pre‐existing conditions)
- Intervention and control description (including dose, schedule, and behavioural support common to all arms)
- Efficacy outcome(s) used in meta‐analysis, including length of follow‐up, definition of abstinence, and biochemical validation of smoking cessation
- Any analysis investigating the interaction between efficacy and participants' depression status
- Harm and tolerability outcomes, including AEs, psychiatric AEs, SAEs, types of SAEs, withdrawals due to treatment
- Sources of funding and declarations of interest

#### Assessment of risk of bias in included studies

We assessed included studies for risks of selection bias (method of random sequence generation and allocation concealment), bias due to an absence of blinding (taking into account both performance and detection bias in a single domain), attrition bias (levels and reporting of loss to follow‐up), and any other threats to study validity, using the Cochrane risk of bias tool (Higgins 2011). For each new study in this update, two review authors (EK, AH) independently assessed each study for each domain, in accordance with risk of bias guidance developed by Cochrane Tobacco Addiction to assess smoking cessation studies. We resolved any disagreements about the assessment through discussion with a third review author.

We considered studies at high risk of performance and detection bias where there was no blinding of participants or personnel or where there was evidence of unblinding; at unclear risk if insufficient information was available with which to judge; and at low risk if the study reported blinding of participants and personnel in detail and there was no evidence of unblinding. We considered studies to be at low risk of attrition bias where over half of the participants were followed up at the longest follow‐up and where numbers followed up were similar across arms (difference < 20%).

#### Measures of treatment effect

##### Smoking cessation

We calculated cessation rates for all studies that reported cessation at least six months following baseline. For each study, we used the strictest available criteria to define cessation, as described above.

Where data were available, we expressed cessation as a risk ratio (RR) for each study. We calculated this as follows: (quitters in treatment group/total randomised to treatment group)/(quitters in control group/total randomised to control group), alongside 95% confidence intervals (CIs). A RR greater than 1 indicates increased likelihood of quitting in the intervention group than in the control condition.

##### Adverse events (AEs) and serious adverse events (SAEs)

We calculated AE rates for all studies that reported adequate data, regardless of study length. Where numerical data were available, we expressed AE and tolerability data as RRs (95% CI). We calculated this as follows: (number of participants reporting (S)AEs in treatment group/total randomised to treatment group)/(number of participants reporting (S)AEs in control group/total randomised to control group). A RR greater than 1 indicates an increased likelihood of experiencing an AE or SAE in the intervention group than in the control condition.

In addition to overall AEs and overall SAEs, we calculated RRs (95% CI) for the following harm and tolerability outcomes, where data were available.

- Psychiatric AEs
- Seizures
- Overdoses
- Suicide attempts
- Death by suicide
- All‐cause mortality
- Dropout due to adverse events
- Insomnia
- Anxiety

#### Unit of analysis issues

We found three cluster‐RCTs eligible for inclusion: Hilberink 2010, Kumar 2020, and Siddiqi 2013. Siddiqi 2013 was not pooled in any meta‐analysis due to substantial heterogeneity of programme effects across clusters. In consultation with a statistician (TF), clustering effects in Hilberink 2010 were deemed to be negligible and therefore no adjustment was made, and results from Kumar 2020 were adjusted for clustering using a reported design effect.

#### Dealing with missing data

As far as possible, we used an intention‐to‐treat (ITT) analysis, with people who dropped out or were lost to follow‐up treated as continuing smokers. Where participants appeared to have been randomised, but were not included in the data presented by the authors (and we were unable to obtain these), we noted this in the study description (see Characteristics of included studies). We extracted numbers lost to follow‐up from study reports and used these to assess the risk of attrition bias.

#### Assessment of heterogeneity

Before pooling studies, we considered both methodological and clinical variance between studies. Where pooling was deemed appropriate, we investigated statistical heterogeneity using the I^2^ statistic (Higgins 2003). This describes the percentage variability in effect estimates that is due to heterogeneity rather than sampling error (chance).

#### Assessment of reporting biases

For three comparisons (bupropion versus placebo/no pharmacological treatment; bupropion and NRT versus NRT alone; bupropion and varenicline versus varenicline alone), we generated funnel plots where the following outcomes had 10 or more studies contributing to an analysis of these outcomes: smoking cessation; SAEs; dropouts due to treatment (i.e. outcomes included in our summary of findings tables). We interpreted these plots to report on potential publication bias, as advised by the *Cochrane Handbook for Systematic Reviews of Interventions* (Higgins 2019).

We therefore generated the following funnel plots:

- bupropion versus placebo/no pharmacological treatment ‐ smoking cessation;
- bupropion versus placebo/no pharmacological treatment ‐ serious adverse events;
- bupropion and nicotine replacement therapy (NRT) versus NRT alone ‐ smoking cessation.

#### Data synthesis

For each type of medication and comparison where more than one eligible trial was identified, we performed separate meta‐analyses of cessation and harms outcomes using Mantel‐Haenszel fixed‐effect methods. We pooled RRs and 95% CIs from individual study estimates to estimate pooled RRs (95% CIs). Where studies contributed more than one intervention arm to a pooled analysis, we split the control arm to avoid double‐counting.

As in the previous update of this review (Howes 2020), we also carried out post hoc, exploratory analyses combining the following comparisons when evaluating AEs, psychiatric AEs, SAEs, and dropouts due to adverse effects.

- Bupropion compared to placebo/no pharmacological treatment
- Bupropion plus NRT compared to NRT alone
- Bupropion plus varenicline compared to varenicline alone

The rationale for this was that these studies all tested the additional effect of bupropion, and there is no reason to expect an interaction for harm and tolerability outcomes. We subgrouped studies by their comparison type, though we acknowledge that these subgroups may currently be underpowered to detect differences between groups.

#### Subgroup analysis and investigation of heterogeneity

Where possible, we separated participant data into the following subgroups within comparisons, to determine whether antidepressants had differential effects on the relevant population or intervention groups.

- Split by mental health diagnoses: mental health diagnoses versus no mental health diagnoses
- Split by level of behavioural support: multisession group support versus multisession individual counselling versus low‐intensity support versus not specified. To be identified as low intensity, support had to be regarded as part of the provision of routine care; that is, time spent with smoker (including assessment for the trial) was less than 30 minutes at the initial consultation, with no more than two further assessment and reinforcement visits.

Where reported, we also extracted data from analyses evaluating a potential interaction between current depression or past history of depression and quit rates. We relied upon the definition of depression used by study authors, which included both formal diagnoses and scores on validated depression scales. This interaction is investigated in more detail in Van der Meer 2013.

#### Sensitivity analysis

We carried out the following sensitivity analyses.

- We excluded studies from meta‐analyses that we judged to be at high risk of bias for any of the assessed bias domains. We judged whether this exclusion notably altered the pooled RRs (95% CI).
- We excluded studies from meta‐analyses with industry support. We did this in two stages: 1) we excluded studies that were funded by the pharmaceutical industry; 2) we excluded studies that were funded by the pharmaceutical industry or where the study medication was provided by the pharmaceutical industry. We judged whether these exclusions notably altered the pooled RRs (95% CI). There is evidence that Industry funding support may influence the findings of studies investigating the effects of smoking cessation medications (Etter 2007; Klemperer 2020).

#### Summary of findings and assessment of the certainty of the evidence

We created summary of findings tables using standard Cochrane methodology (Higgins 2019), for the following comparisons, which we judged to be most clinically relevant.

- Bupropion compared to placebo/no pharmacological treatment
- Bupropion plus NRT compared to NRT alone
- Bupropion plus varenicline compared to varenicline alone

We judged these comparisons to be of most relevance because bupropion is currently the only antidepressant used as a first‐line pharmacotherapy for smoking cessation worldwide.

Following standard Cochrane methodology (Higgins 2019), we used GRADEpro GDT software and the five GRADE considerations (risk of bias, consistency of effect, imprecision, indirectness, and publication bias) to assess the certainty of the body of evidence for three outcomes: smoking cessation, SAEs, and dropouts due to adverse events of the treatment, and to draw conclusions about the certainty of the evidence (Schünemann 2013). We chose these outcomes as they are important factors to consider regarding pharmaceutical efficacy, harms, and tolerability, and are therefore useful to both clinicians and patients when deciding whether to provide or use a smoking cessation pharmacotherapy.

## Results

### Description of studies

See Characteristics of included studies; Characteristics of excluded studies; Characteristics of ongoing studies

#### Results of the search

The most recent literature search for this update generated 116 records. After we removed duplicates, 105 records remained for title and abstract screening. We ruled out 84 irrelevant records at this stage, leaving 21 records for full‐text screening. At this stage, we identified four new studies for inclusion (Abdelghany 2022; Kumar 2020; Qin 2021; Zhang 2022) and two new ongoing studies (NCT04604509; NCT05205811). We also identified two new references linked to two studies previously included in the review, and six new included studies through further consideration of studies previously excluded from the previous version of this review (Howes 2020). In most cases, these studies had been excluded in the first instance due to the unavailability of key data (Evins 2008; Hilberink 2010; Hoch 2006; Rovina 2003; Schepis 2006; Swanson 2003). See Figure 1 for full details of record/study flow information for the most recent updated search. Of note, we also discovered that two records previously included in this review as two separate studies were reporting on one study (Weinberger 2008). Therefore, these records have now been merged into a single study. Data from this study had not previously been included in meta‐analyses and so did not pose a threat to the integrity of previous analyses.

**1:**
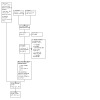
PRISMA flow diagram for April 2022 search update

#### Included studies

In total, we included 10 new studies at this update (four newly identified from this 2022 search: Abdelghany 2022; Kumar 2020; Qin 2021; Zhang 2022; six were previously excluded in the earlier review version (Howes 2020) and have now been reassessed and included: Evins 2008; Hilberink 2010; Hoch 2006; Rovina 2003; Schepis 2006; Swanson 2003), yielding a total of 124 included trials, including 48,832 participants. The new included studies all investigated the use of bupropion. Further details of these newly included, as well as previously included, studies are recorded in the Characteristics of included studies tables.

##### Bupropion

Overall, we included 99 studies of bupropion. The majority of trials were conducted in North America, but we also included studies from Egypt (Abdelghany 2022), Australia (Myles 2004); Brazil (Haggsträm 2006); China (Sheng 2013; Qin 2021); Europe (Aubin 2004; Dalsgarð 2004; Fossati 2007; Górecka 2003; Rovina 2009; Stapleton 2013; Wagena 2005; Wittchen 2011; Zellweger 2005); India (Kumar 2020; Johns 2017; Singh 2010); Israel (Planer 2011); New Zealand (Holt 2005); Pakistan (Siddiqi 2013); Taiwan (NCT00495352); and Turkey (Benli 2017; Uyar 2007; Zincir 2013). Three studies were carried out across multiple continents (Anthenelli 2016; Tonnesen 2003; Tonstad 2003).

A number of studies specifically recruited cohorts of participants with health conditions, including:

- alcohol use disorder (Grant 2007; Hays 2009; Karam‐Hage 2011);
- bipolar disorder (Weinberger 2008);
- cancer (Schnoll 2010);
- cardiovascular disease (Eisenberg 2013; Planer 2011; Rigotti 2006; Tonstad 2003);
- chronic obstructive pulmonary disease (Górecka 2003; Tashkin 2001; Wagena 2005; Hilberink 2010);
- mild depression (Moreno‐Coutino 2015);
- psychiatric conditions (Anthenelli 2016; Evins 2008);
- schizophrenia (Evins 2001; Evins 2005; Evins 2007; Fatemi 2013; George 2002; George 2008; NCT00495352; Weiner 2012);
- post‐traumatic stress disorder (Hertzberg 2001);
- tuberculosis or suspected tuberculosis (Siddiqi 2013; Kumar 2020).

Three of the studies in people with cardiovascular disease, and one other, enrolled hospital inpatients (Eisenberg 2013; Planer 2011; Rigotti 2006; Simon 2009).

Included trials also studied specific populations of people who smoked, including:

- adolescents (Gray 2011; Gray 2012; Killen 2004; Muramoto 2007);
- African‐Americans (Ahluwalia 2002; Cox 2012);
- healthcare workers (Zellweger 2005);
- hospital staff (Dalsgarð 2004);
- people with a low‐income (NCT00308763);
- Maori (Holt 2005);
- males (Rose 2017);
- people awaiting surgery (Myles 2004);
- people who had previously failed to quit smoking using bupropion (Gonzales 2001; Selby 2003);
- people who had recently failed to quit using NRT (Hurt 2003; Rose 2013; Rose 2014);
- sailors (Swanson 2003).

More than half the bupropion studies followed participants for at least 12 months from the start of treatment or the target quit day. Thirty‐four studies followed up participants for six months. The duration of follow‐up was under six months for 14 of the included studies, was of unknown duration for seven studies, and one study measured number of days abstinent rather than numbers abstinent at a particular time point (Perkins 2013). We included these studies as they measured harms and do not contribute efficacy data to our meta‐analyses. Schepis 2006 planned to do a six‐month analysis, but no data were available for this time point. Likewise, Elsasser 2002 planned to do a 52‐week study, but the available abstract only includes data up to the 12‐week follow‐up. Neither study contributes to our efficacy analyses.

In those studies which met or exceeded the six‐month follow‐up threshold, the majority reported an outcome of sustained (prolonged) abstinence. However, in 32 studies, only point prevalence rates were given, or the definition of abstinence was unclear.

Sixty‐two trials evaluated bupropion for smoking cessation as a single pharmacotherapy versus placebo/no pharmacological treatment, and three studies compared different doses of bupropion (Hurt 1997; Muramoto 2007; Swan 2003). Both Muramoto 2007 and Swan 2003 compared a 150 mg dose per day with a 300 mg dose per day, whereas Hurt 1997 looked at 100 mg per day versus 150 mg per day versus 300 mg per day. One study looked at different durations of bupropion (Rovina 2003), at seven and 19 weeks. We pooled studies in which bupropion was used in combination with another pharmacotherapy or versus another pharmacotherapy in separate comparisons.

##### Nortriptyline

We included 10 studies of the tricyclic antidepressant, nortriptyline, in this review. Hall and colleagues conducted three trials (Hall 1998; Hall 2002; Hall 2004), and Prochazka and colleagues conducted two (Prochazka 1998; Prochazka 2004), with all these trials conducted in the USA. One study was conducted in Australia (Richmond 2013), two in Brazil (Da Costa 2002; Haggsträm 2006), one in the Netherlands (Wagena 2005), and one in the UK (Aveyard 2008).

Richmond 2013 was the only study to be conducted in a specialist population, recruiting male prisoners who had been incarcerated for at least one month and had at least six months remaining of their sentences.

All studies were placebo‐controlled. They used nortriptyline doses of 75 mg/day to 100 mg/day or titrated doses to serum levels recommended for depression during the week prior to the quit date.

Treatment duration ranged from 12 to 14 weeks. Nearly all studies used a definition of cessation based on a sustained period of abstinence. Aveyard 2008, Hall 1998, Hall 2002, Hall 2004, and Richmond 2013 reported outcomes at 12 months of follow‐up or longer, and the other six studies had a maximum follow‐up of six months. The three studies by Hall and colleagues used factorial designs to test nortriptyline versus placebo crossed with different intensities of behavioural support (Hall 1998; Hall 2002; Hall 2004). Conversely, the remaining studies provided a set amount of behavioural support to all participants, ranging from brief behavioural counselling to repeated group and individual sessions. Six studies tested nortriptyline as a monotherapy, and four studies tested nortriptyline as an adjunct to NRT.

##### Selective serotonin reuptake inhibitors (SSRIs)

###### Fluoxetine

We have included seven studies of fluoxetine in this review, with two of these studies identified for inclusion in the current update (Minami 2014; NCT00578669).

Most of these trials took place in the USA (Brown 2014; Minami 2014; NCT00578669; Niaura 2002; Saules 2004; Spring 2007), with one in Iceland (Blondal 1999). Participants were recruited from clinics (Blondal 1999; Brown 2014; Niaura 2002; Saules 2004; Spring 2007), the community (Minami 2014), or through an unknown recruitment method (NCT00578669).

Brown 2014 was the only study to be conducted in a specialist population, recruiting smokers with elevated depressive symptoms.

Six of these studies conducted follow‐up to at least six months for cessation outcomes. Minami 2014 had a follow‐up duration of fewer than six months, so we only evaluated adverse events data for this study.

Four studies used varying doses of fluoxetine as a single pharmacotherapy: Niaura 2002 compared a 30 mg daily dose, a 60 mg daily dose, or placebo for 10 weeks; Spring 2007 used 60 mg or placebo for 12 weeks; NCT00578669 compared 20 mg daily for eight weeks preceding and following the target quit date to placebo. Minami 2014 also compared fluoxetine as a monotherapy (20 mg daily for eight weeks prior to and following the target quit date) to placebo only.

The remaining three trials investigated fluoxetine as an adjunct to NRT, and used similar doses of fluoxetine: Blondal 1999 used 20 mg/day or placebo for three months as an adjunct to nicotine inhaler; Saules 2004 used 20 mg/day or 40 mg/day or placebo for 10 weeks as an adjunct to nicotine patch; and Brown 2014 compared 10 weeks of 20 mg daily fluoxetine, 16 weeks of 20 mg daily fluoxetine, or no additional treatment in participants using a nicotine patch for eight weeks.

###### Paroxetine

One trial assessed paroxetine (20 mg, 40 mg, or placebo) for nine weeks as an adjunct to nicotine patch (Killen 2000). It was conducted in the USA, with participants recruited from the community. It measured smoking cessation (defined as seven‐day point prevalence abstinence) at six months' follow‐up.

###### Sertraline

One trial with six months' follow‐up assessed sertraline (200 mg/day) for 11 weeks versus placebo in conjunction with six individual counselling sessions. All participants had a past history of major depression (Covey 2002).

##### Monoamine oxidase inhibitors

###### Moclobemide

Moclobemide was tested for smoking cessation in one placebo‐controlled trial, carried out in France (Berlin 1995). Participants were recruited using advertisements in community healthcare settings. Treatment with 400 mg/day began one week before quit day and continued for two months, reducing to 200 mg/day for a further month. No behavioural counselling was provided. Final follow‐up for smoking cessation (defined as prolonged abstinence) was at 12 months.

###### Selegiline

We included five long‐term trials testing selegiline in this review. They were carried out in the USA (George 2003; Kahn 2012; Killen 2010; Weinberger 2010), and Israel (Biberman 2003). All studies recruited participants from the community.

Almost all studies delivered selegiline as a monotherapy compared to placebo, excluding Biberman 2003, which used a combination of selegiline and nicotine patch compared to placebo.

Three studies used 10 mg/day of oral treatment (Biberman 2003; George 2003; Weinberger 2010), and two used 6 mg/day of patch treatment (Kahn 2012; Killen 2010). The nicotine patches also used in Biberman 2003 delivered 21 mg/day of nicotine for eight weeks. Three studies had treatment durations of nine weeks (George 2003; Kahn 2012; Weinberger 2010), one had a treatment duration of eight weeks (Killen 2010), and one continued therapy for 26 weeks (Biberman 2003). Three of the studies completed follow‐up at six months (George 2003; Kahn 2012; Killen 2010), and two continued follow‐up to 12 months (Biberman 2003; Weinberger 2010).

###### Lazabemide

Berlin 2002 is the only study of lazabemide included in this review. Due to its nature as a dose‐finding, exploratory study, its follow‐up period for smoking cessation was only eight weeks. Therefore, we only consider its data related to harms within this review.

The study was conducted in both France and Belgium; however, the method of participant recruitment is not reported. Participants were given either 50 mg lazabemide, 100 mg lazabemide or placebo. It was halted early due to liver toxicity observed in trials of the medication for other indications.

##### EVT302

Berlin 2012 is the only study of EVT302 included in this review. Its follow‐up for smoking cessation was only eight weeks. Therefore, we only consider its data related to harms within this review.

The study was conducted in Germany, with participants recruited through media advertisements. It compared EVT302 monotherapy (5 mg/day for one week preceding and seven weeks following the target quit date) with placebo. It additionally compared EVT302 combination therapy with nicotine patch (21 mg/day for seven weeks post‐target quit date) versus placebo EVT302 and nicotine patch.

##### Venlafaxine

Cinciripini 2005 is the only study of venlafaxine included in this review. It recruited from the community and compared venlafaxine at a dose of up to 225 mg/day with placebo. All participants also received nicotine patches and nine brief individual counselling sessions; follow‐up was for 12 months.

##### Hypericum (St John’s wort)

Three studies of hypericum are included (Barnes 2006; Parsons 2009; Sood 2010), with Barnes 2006 newly included at this update. These studies took place in the USA (Sood 2010), and the UK (Barnes 2006; Parsons 2009). Participants were recruited from the community (Barnes 2006; Sood 2010), and stop‐smoking clinics (Parsons 2009).

All three studies reported prolonged abstinence at six months. Barnes 2006 compared 300 mg/day to 600 mg/day, starting one week prior to the target quit date and continuing for 12 weeks thereafter. Parsons 2009 compared 14 weeks of 900 mg/day of St John's wort to placebo, starting two weeks prior to target quit date and continuing for 12 weeks thereafter. Sood 2010 compared 900 mg/day, 1800 mg/day, and placebo for 12 weeks.

##### S‐adenosyl‐L‐methionine (SAMe)

Sood 2012 is the only study of SAMe included in this review. It compared 1600 mg/day or 800 mg/day SAMe to placebo for eight weeks, with smoking cessation follow‐up at six months.

#### Ongoing studies

We identified the following four ongoing studies (two from the previous review version: NCT03326128; NCT03342027) that may be relevant for inclusion in the review when complete.

- NCT03326128: compares two doses of bupropion (300 mg/day to 450 mg/day, starting four weeks prior to and following the target quit date).
- NCT03342027: a factorial trial comparing bupropion to placebo, as well as an eight‐session tailored behavioural intervention.
- NCT04604509: varenicline or NRT plus bupropion and counselling compared to varenicline or NRT plus counselling only.
- NCT05205811: compares 300 mg bupropion plus e‐cigarette to placebo plus e‐cigarette.

We present further details of these ongoing studies in the Characteristics of ongoing studies table.

#### Excluded studies

We list our reasons for excluding 105 studies in Characteristics of excluded studies. Reasons for excluding seven studies at full‐text stage for this update specifically (ChiCTR1900020676; Ghorbani Behnam 2019; Li 2019; Ruehle 2021; Sanchez 2019; Schiavon 2018; TCTR20190506002), are also summarised in Figure 1.

### Risk of bias in included studies

Overall, we judged 14 studies to be at low risk of bias (low risk of bias across all domains), 34 at high risk of bias (high risk of bias in at least one domain), and the remaining 76 at unclear risk of bias. Reasons for the judgements made below are detailed in the Characteristics of included studies table, and a summary illustration of the risk of bias profile across studies is shown in Figure 2.

**2:**
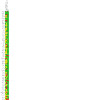
Risk of bias summary: review authors' judgements about each risk of bias item for each included study

#### Allocation

We assessed selection bias through investigating methods of random sequence generation and allocation concealment for each study. We rated 49 studies at low risk for random sequence generation, one at high risk (Moreno‐Coutino 2015), and the remainder at unclear risk. We judged 34 studies to be at low risk for allocation concealment, four at high risk, and the remainder at unclear risk. When assessing both random sequence generation and allocation concealment, we assessed studies to be at unclear risk where there was insufficient methodological information available to be sure whether adequate measures had been taken to avoid selection bias.

#### Blinding

We assessed any risk of bias linked to blinding as one domain. However, we took into account both performance and detection bias when making this judgement. We judged 36 studies to be at low risk of bias for this domain, 23 at high risk, and the remainder at unclear risk. Where studies stated that they were "double‐blind" only, with no explicit clarification of who was blinded, we judged this to be unclear risk.

#### Incomplete outcome data

We judged studies to be at a low risk of attrition bias where the numbers of participants lost to follow‐up were clearly reported, the overall number lost to follow‐up was not more than 50%, and the difference in loss to follow‐up between groups was no greater than 20%. This is in accordance with risk of bias guidance produced by Cochrane Tobacco Addiction for assessing smoking cessation studies. We judged 74 of the studies to be at low risk of bias, 15 at high risk, and the remainder at unclear risk.

#### Other potential sources of bias

We found five studies with other sources of potential bias beyond those domains detailed previously. We judged Hilberink 2010 to be at high risk of bias as it was a cluster‐RCT that was not adjusted for clustering effects. Kumar 2020 also did not adjust for clustering and the baseline characteristics were unbalanced between arms, resulting in a judgment of high risk of bias. Siddiqi 2013 demonstrated substantial heterogeneity of programme effects across the different clusters of their cluster‐RCT. Twenty per cent of participants in the control arm smoked only hookah (no cigarettes) compared with 4% in the intervention arm. We judged that this put the study at high risk of bias. The Weiner 2012 study stated that there was insufficient study drug available to meet demand. It is unclear how this was dealt with and whether it is accounted for in the dropouts reported. We judged this to be an unclear risk of bias. Finally, the Zincir 2013 study stated that there were no adverse events recorded during their study. This seems highly unlikely according to the common definition of adverse events, and there is no detail given of how adverse events were measured in the study. We have therefore judged the study at high risk of bias for this reason. We judged all remaining studies to be at low risk.

### Effects of interventions

See: Table 1; Table 2; Table 3

#### Bupropion versus placebo/no pharmacological treatment

##### Smoking cessation

Pooled data showed bupropion was effective when compared to placebo or no pharmacological treatment to assist smoking cessation. Our meta‐analysis included 50 trials in which bupropion was the sole pharmacotherapy, with 18,577 participants (pooled risk ratio (RR) 1.60, 95% confidence interval (CI) 1.49 to 1.72; I^2^ = 16%; high‐certainty evidence; Analysis 1.1; Table 1). The results were not sensitive to the exclusion of studies we judged to be at high risk of bias overall or studies that received industry support (see Table 4). We excluded one cluster‐RCT of bupropion versus no pharmacotherapy from our meta‐analysis due to substantial heterogeneity of programme effects across clusters. This trial detected no clear evidence of a difference between the bupropion and no pharmacotherapy arms (both groups received behavioural support) for smoking cessation at any follow‐up point (adjusted RR at 6‐month follow‐up: 1.1, 95% CI 0.5 to 2.3; 1299 participants), although there was substantial imprecision, demonstrated by CIs reflecting both potential benefit and harm of bupropion on quit rates (Siddiqi 2013). In addition, Urdapilleta‐Herrera 2013 did not report sufficient information for us to discern the denominators used in their percentage calculations of cessation. They reported that they found no significant differences in quit rates at both 6‐month (bupropion 48% versus placebo 52%) and 12‐month follow‐ups (bupropion 53% versus placebo 47%).

**1:** Sensitivity analyses excluding industry‐supported studies

**Comparison and outcome**	**RR and CI excluding industry‐funded studies**	**RR and CI excluding studies with funding or medication provided by industry**
Analysis 1.1	1.53 [1.37, 1.71]; studies = 26	1.45 [1.26, 1.66]; studies = 15
Analysis 1.2	1.53 [1.37, 1.71]; studies = 26	1.44 [1.25, 1.67]; studies = 14
Analysis 1.3	1.62 [1.47, 1.78]; studies = 28	1.60 [1.43, 1.80]; studies = 18
Analysis 1.4	1.30 [1.18, 1.43]; studies = 10	1.28 [1.15, 1.42]; studies = 9
Analysis 1.5	1.10 [0.69, 1.75]; studies = 5	1.10 [0.69, 1.75]; studies = 5
Analysis 1.6	2.08 [0.93, 4.64]; studies = 4	2.27 [0.46, 11.17]; studies = 2
Analysis 1.7	1.72 [1.46, 2.03]; studies = 10	1.80 [1.40, 2.32]; studies = 7
Analysis 1.8	0.88 [0.61, 1.26]; studies = 11	0.88 [0.61, 1.26]; studies = 11
Analysis 1.9	2.61 [0.11, 60.51]; studies = 6	2.61 [0.11, 60.51]; studies = 6
Analysis 1.10	Not estimable; studies = 1	Not estimable; studies = 1
Analysis 1.11	Not estimable; studies = 4	Not estimable; studies = 4
Analysis 1.12	Not estimable; studies = 5	Not estimable; studies = 5
Analysis 1.13	1.51 [0.44, 5.27]; studies = 6	1.51 [0.44, 5.27]; studies = 6
Analysis 1.14	1.12 [0.84, 1.50]; studies = 10	1.05 [0.77, 1.43]; studies = 8
Analysis 2.1	0.87 [0.61, 1.24]; studies = 13	1.60 [1.43, 1.80]; studies = 7
Analysis 2.2	1.21 [1.03, 1.43]; studies = 3, no difference from original analysis	1.24 [1.00, 1.55]; studies = 2
Analysis 2.3	Not estimable. No difference from original analysis; studies = 1	Not estimable; no difference from original analysis; studies = 1
Analysis 2.4	1.62 [0.72, 3.65]; studies = 2	Not estimable, studies = 0
Analysis 2.5	1.26 [0.60, 2.65]; studies = 1	Not estimable; studies = 0
Analysis 2.6	2.06 [0.20, 21.67]; studies = 2	Not estimable; studies = 1
Analysis 2.7	2.92 [0.12, 71.39], no difference from original analysis; studies = 1	Not estimable, studies = 0
Analysis 2.8	Not estimable. No difference from original analysis; studies = 0	Not estimable. No difference from original analysis; studies = 0
Analysis 2.9	Not estimable. No difference from original analysis; studies = 0	Not estimable. No difference from original analysis; studies = 0
Analysis 2.10	0.68 [0.12, 3.98]; studies = 1	0.68 [0.12, 3.98]; studies = 1
Analysis 2.11	1.04 [0.16, 6.83]; studies = 2	Not estimable; studies = 0
Analysis 3.1	No industry‐funded studies, i.e. no difference from original analysis.	0.93 [0.59, 1.44]; studies = 1
Analysis 3.2	No industry‐funded studies, i.e. no difference from original analysis.	1.11 [1.04, 1.20]; studies = 3
Analysis 3.3	4.64 [1.01, 21.28]; studies = 1	Not estimable; studies = 0
Analysis 3.4	2.10 [0.54, 8.11]; studies = 1	2.10 [0.54, 8.11]; studies = 1
Analysis 3.5	2.10 [0.54, 8.11]; studies = 1	2.10 [0.54, 8.11]; studies = 1
Analysis 3.6	0.94 [0.40, 2.19]; studies = 4	0.71 [0.12, 4.17]; studies = 2
Analysis 3.7	No industry‐funded studies, i.e. no difference from original analysis.	Not estimable; studies = 0
Analysis 3.8	Not estimable; studies = 1	Not estimable; studies = 0
Analysis 3.9	0.34 [0.01, 8.40]; studies = 2	Not estimable; studies = 1
Analysis 3.10	No industry‐funded studies, i.e. no difference from original analysis.	Not estimable; studies = 0
Analysis 3.11	No industry‐funded studies, i.e. no difference from original analysis.	Not estimable; studies = 0
Analysis 3.12	1.01 [0.44, 2.31]; studies = 3	1.07 [0.07, 16.86]; studies = 1

**CI:** confidence interval; **RR:** risk ratio

We found no evidence suggesting that the effect of bupropion on smoking cessation was dependent upon the level of behavioural support offered to people stopping smoking. Three trials directly compared bupropion and placebo in factorial designs varying the behavioural support. There was no evidence from any of the three trials that the efficacy of bupropion differed between the lower and higher levels of behavioural support (Hall 2002; McCarthy 2008), or by the type of counselling approach used (Schmitz 2007). We also carried out a between‐study subgroup analysis of the possible interaction with behavioural support. We did this by classifying studies into low and high intensities of behavioural support (further split into delivery to a group or to individuals), using the criteria set in the Cochrane Review of NRT versus control (Hartmann‐Boyce 2018). Low‐intensity support consisted of less than 30 minutes at the initial consultation, with no more than two further assessment and reinforcement visits. Only two small trials met this criteria (Abdelghany 2022; Myles 2004). We found no evidence of a difference between subgroups (I^2^ = 0%; Analysis 1.2).

One trial directly compared bupropion and placebo in a cohort of participants with mental health disorders to a cohort without (Anthenelli 2016). There was no evidence indicating that the effect of bupropion depended upon whether people had or did not have a psychiatric disorder. We also carried out a between‐study subgroup analysis to assess the potential interaction between cessation rates and mental health disorders. We did this by pooling studies (or subgroups of studies) into groups, depending upon whether the participants were recruited specifically because they had a mental health disorder or they represented the general population (including some studies that excluded people with current mental health disorders). Some of these groups included people with serious mental health disorders, such as people with schizophrenia (Evins 2001; Evins 2005; George 2002), or other disorders, including post‐traumatic stress disorder (PTSD; Hertzberg 2001), and a mix of mental health disorders (Anthenelli 2016). We found no evidence of a differential effect of bupropion on cessation between subgroups (I^2^ = 15%; Analysis 1.3).

###### Depression

Four studies comparing bupropion to placebo/no pharmacological treatment analysed whether there was any interaction between depression and smoking quit rates (Anthenelli 2016 (analysis reported in West 2018); Aubin 2004; Cinciripini 2018; Kalman 2011). We did not find any evidence of this (Table 5). 

**2:** Depression as a moderator of the relationship between antidepressants and smoking cessation

**Study ID**	**Antidepressant**	**Direction of relationship**	**Evidence for interaction**
Anthenelli 2016	Bupropion	None	"Varenicline, bupropion and NRT were all effective in smokers with mental health problems (assessed with a number of variables, e.g. diagnostic history, HADS, use of psychotropic medication), and their relative efficacy was similar to that in smokers without a psychiatric history."
Aubin 2004	Bupropion	None	"A similar subgroup analysis performed according to previous history of depression (evaluated by the MINI questionnaire) also failed to reveal an interaction with bupropion treatment."
Aveyard 2008	Nortriptyline	None	"Participants randomised to nortriptyline plus nicotine replacement therapy for smoking cessation experienced less depression (OR 0.15) and anxiety early in the quit attempt when the risk of return to smoking is at its highest than those randomised to placebo plus nicotine replacement therapy. Contrary to expectations, no evidence was found that this led to greater abstinence."
Cinciripini 2018	Bupropion	None	"Several measures failed to demonstrate significant effects as a function of time, treatment, or the interaction of treatment and time. For example, CES‐D scales including Depressive Affect, Interpersonal Relations, Positive Affect, and Somatic Symptoms, failed to demonstrate any effects of treatment or any treatment by time interactions."
Da Costa 2002	Nortriptyline	Negative	"The best results were obtained with educational intervention, in those patients having no personal history of depression, who received the active drug. A negative history of depression was, however, the most important factor for the success of the treatment."
George 2003	Selegiline	None (history), negative (current)	“There was no significant influence of a past history of major depression on smoking cessation outcomes (B = ‐0.49, SE = 0.90, Wald Statistic = 0.29, df = 1, p = .59), and when past history of major depression was entered into the logistic regression model as a covariate, it did not predict treatment failure with selegiline study medication (medication past history of depression status interaction: B = ‐0.02, SE = 1.03, Wald statistic = 0.00, df = 1, p = .98)." and "Furthermore, bivariate logistic regression analysis confirmed that having depressive symptoms at baseline negatively predicted smoking cessation outcomes with SEL on this continuous abstinence measure (B = 18.9, SE = 0.58, Wald statistic = 1048.9, df = 1, P < .01)."
Hall 2002	Bupropion, nortriptyline	Positive (for bupropion)	“There were higher abstinence rates for bupropion than nortriptyline for participants with a history of depressive disorder"
Kahn 2012	Selegiline	None	"At the final HAM‐D assessment, the selegiline group (n = 90) reported a mean increase of 0.41 points and the placebo group (n = 85) reported a mean increase of 0.21 points. The difference between treatment groups was not statistically significant (t test, p = .65)."
Kalman 2011	Bupropion	None	“Interaction effects between medication and tobacco dependence and medication and depressive symptoms were also nonsignificant.”
Killen 2000	Paroxetine	None	"A stepwise logistic regression analysis was used to examine the association of abstinence at Week 26 with the variables [including depression scores] listed in Table 1. None of these variables were prospectively associated with abstinence."
Saules 2004	Fluoxetine	None	“Examination of pre‐specified subgroups (i.e., gender, race, and history of major depressive disorder) did not reveal significant differences in smoking cessation by group”
Spring 2007	Fluoxetine	None	Fluoxetine initially enhanced cessation for smokers with a history of major depression (P = .02) but subsequently impaired cessation regardless of depressive history.
Stapleton 2013	Bupropion	Positive	“There was some evidence that the relative effectiveness of bupropion and NRT differed according to depression (χ2 = 2.86, P = 0.091), with bupropion appearing more beneficial than NRT in those with a history of depression (29.8 versus 18.5%)."
Wagena 2005	Bupropion, nortriptyline	Positive (for bupropion)	“Results indicated that bupropion SR [sustained release] treatment was efficacious in helping smokers who were classified as depressed in achieving prolonged abstinence from smoking throughout the 26‐week period. The number of depressed participants from the nortriptyline‐treated group was considered too low to study this relationship."

**CES‐D:** Center for Epidemiologic Studies Depression; **df:** degrees of freedom; **HADS:** Hospital Anxiety and Depression scale; **HAM‐D:** Hamilton Depression Rating Scale; **MINI:** Mini‐International Neuropsychiatric Interview; **NRT:** nicotine replacement therapy; **OR:** odds ratio; **SE:** standard error

##### Harms

A meta‐analysis of 21 studies, including 10,931 participants, found evidence that bupropion resulted in more non‐serious adverse events (AEs) than placebo or a non‐pharmacological treatment (RR 1.14, 95% CI 1.11 to 1.18; I^2^ = 62%; Analysis 1.4). There was some evidence of heterogeneity; however, all but one effect estimate showed the same direction of effect (higher AEs in the bupropion arms). There was also evidence of higher rates of psychiatric AEs in the bupropion arms, with much lower levels of heterogeneity (RR 1.25, 95% CI 1.15 to 1.36; I^2^ = 13%; 8 studies, 4494 participants; Analysis 1.5). Looking specifically at anxiety and insomnia, the effects also demonstrated higher rates in the bupropion groups (Analysis 1.6; Analysis 1.7). The Perkins 2013 study did not provide sufficient data to be included in meta‐analyses; however, trialists found that AEs experienced by those in the bupropion and placebo groups were mild, with most participants reporting no AEs at all. Relative to placebo, people using bupropion were less likely to report agitation and more likely to report decreased appetite. The authors deemed these effects to reflect a relief of withdrawal symptoms, and not actual 'adverse effects'.

We found moderate‐certainty evidence from 10,958 participants in 23 RCTs that rates of serious adverse events (SAEs) were slightly higher in the bupropion arms than the placebo/no pharmacological treatment arms; however, there was some imprecision, with 95% CIs also incorporating no difference (RR 1.16, 95% CI 0.90 to 1.48; I^2^ = 0%; Analysis 1.8; Table 1). In eight of the studies, no SAEs were reported and so these studies do not contribute to the pooled estimate.

Considering specific events, meta‐analyses combining seizures (Analysis 1.9), overdoses (Analysis 1.10), and suicide attempts (Analysis 1.11) across studies all had point estimates favouring placebo or no pharmacological treatment but with serious imprecision: 95% CIs also incorporated lower rates in the bupropion groups. For death by suicide, only one event was reported across 14 studies in 8822 participants, which took place in the placebo/no pharmacological treatment group. All‐cause mortality resulted in an effect estimate favouring bupropion, with no statistical heterogeneity (I^2^ = 0%) detected; however, there was substantial imprecision, with 95% CIs incorporating the potential for both benefit and harm (Analysis 1.13). Analyses 1.9 to 1.13 all included studies where zero events were reported and so these studies could not contribute to the pooled estimate.

##### Tolerability

Twenty‐five RCTs, including 12,346 participants, reported dropouts due to treatment for this comparison. Our meta‐analysis provided high‐certainty evidence that there were higher dropout rates due to treatment when using bupropion than when using placebo or no pharmacological treatment (RR 1.44, 95% CI 1.27 to 1.65; I^2^ = 2%; Analysis 1.14; Table 1).

#### Bupropion plus nicotine replacement therapy (NRT) versus NRT alone

##### Smoking cessation

There was moderate unexplained statistical heterogeneity and imprecision when pooling the results of 15 studies comparing bupropion plus NRT to NRT alone for smoking cessation (RR 1.17, 95% CI 0.95 to 1.44; I^2^ = 43%; 4117 participants; low‐certainty evidence; Analysis 2.1; Table 2). Thus, the analysis found no clear evidence of a benefit of using bupropion plus NRT over using NRT alone. Ten of the 15 studies used nicotine patches alone, two studies provided participants with nicotine lozenges (Piper 2009; Smith 2009), two offered a choice of NRT (Hilberink 2010; Stapleton 2013), and one offered gum alone (Abdelghany 2022). Therefore, none appeared to test combination NRT (patch and another form) in addition to bupropion. Splitting the analysis into subgroups by the type of NRT used did not explain the heterogeneity detected (I^2^ = 0% for subgroup differences), nor did the exclusion of studies that did not use a bupropion placebo in the control arm (Smith 2009; Stapleton 2013). In addition, removing the six studies deemed to be at an overall high risk of bias did not change the interpretation of the pooled effect estimate. Sensitivity analyses excluding studies with industry support did not indicate that our findings were sensitive to the inclusion of these studies (see Table 4). Although the direction of the effect estimate changed when we excluded studies funded by the pharmaceutical industry, or studies where the pharmaceutical industry had supplied the medication, 95% CIs still encompassed evidence of benefit as well as harm.

###### Depression

Evins 2008 considered smokers with a diagnosis of unspecified dissociative disorder (major depression, minor depression, or dysthymic disorder), but due to insufficient follow‐up time, the study data only contribute to analyses of harms and not efficacy.

##### Harms

A meta‐analysis of three studies, including 339 participants, found evidence that bupropion and NRT in combination resulted in more non‐serious AEs than NRT alone (RR 1.21, 95% CI 1.03 to 1.43; I^2^ = 0%; Analysis 2.2). There was little evidence reporting on psychiatric effects, with only one very small study reporting on these generally (Analysis 2.3). Three studies involving 1218 participants reported on anxiety specifically. The pooling of these provided some evidence that rates of anxiety were higher in the bupropion plus NRT arm compared with the NRT alone arm. However, the CIs also incorporated the possibility of no difference between treatments, and there was moderate statistical heterogeneity (I^2^ = 47%; Analysis 2.4). Two studies with 556 participants provided evidence that rates of insomnia were higher in the 'bupropion plus NRT' arms (Analysis 2.5).

Evidence from 657 participants in four RCTs resulted in very imprecise evidence due to a very low number of SAEs (n = 5) reported across all study arms, with 95% CIs incorporating potential benefit and harm of the intervention, as well as no difference (RR 1.52, 95% CI 0.26 to 8.89; I^2^ = 0%; low‐certainty evidence; Analysis 2.6; Table 2). Two of the four studies did not contribute to the pooled estimate as no SAEs were reported in the studies.

Considering specific events, only single studies reported on seizures (Analysis 2.7), suicide attempts (Analysis 2.8), and death by suicide (Analysis 2.9). For the former, one person in the bupropion plus NRT arm reported a seizure, and in the latter two cases, no events were reported. Only two studies reported on all‐cause mortality, with a total of five events reported in just one of the studies. This led to an extremely imprecise effect estimate favouring the NRT‐only arm (RR 0.68, 95% CI 0.12 to 3.98; I^2^ = not applicable; Analysis 2.10).

##### Tolerability

Three RCTs, including 737 participants, reported dropouts due to treatment for this comparison. However, one of the studies did not contribute to the pooled analysis due to a lack of events. The resulting point estimate favoured NRT alone, although the lower 95% CI incorporated no difference (RR 1.67, 95% CI 0.95 to 2.92; I^2^ = 0%; low‐certainty evidence; Analysis 2.11; Table 2).

#### Bupropion plus varenicline versus varenicline alone

##### Smoking cessation

Our analysis found moderate‐certainty evidence that the combination of bupropion and varenicline may result in higher smoking cessation rates than varenicline alone (RR 1.21, 95% CI 0.95 to 1.55; I^2^ = 15%; 3 studies, 1057 participants; Analysis 3.1; Table 3). However, the CI also encompassed the possibility of no clinically significant difference in quit rates. Of note, Johns 2017 also investigated this comparison but did not contribute to our meta‐analysis due to insufficient data in the available abstract. The investigators found that, compared to bupropion monotherapy and varenicline monotherapy, bupropion in combination with varenicline produced significant increases in abstinence at six months and presented the following odds ratios that may relate to this comparison: OR 1.52, 95% CI 1.00 to 2.30; OR 1.72, 95% CI 1.06 to 2.67. Either of these ORs would provide further support for the effects demonstrated in our meta‐analysis.

We did not carry out a sensitivity analysis to account for risk of bias as we did not judge any of the studies in the analysis to be at high risk. A sensitivity analysis excluding studies with industry support did not indicate that our findings were sensitive to the inclusion of these studies (see Table 4).

###### Depression

None of the relevant included studies investigated a potential link between depression and quit rates.

##### Harms

A meta‐analysis of four studies, including 1043 participants, found evidence that bupropion plus varenicline resulted in more non‐serious AEs then varenicline alone (RR 1.09, 95% CI 1.02 to 1.17; Analysis 3.2); however, this result should be treated with caution due to substantial statistical heterogeneity (I^2^ = 78%). Two studies reported on psychiatric AEs specifically, with the result again indicating larger numbers in the 'bupropion plus varenicline' arms (RR 1.15, 95% CI 1.03 to 1.30; I^2^ = 7%; 835 participants; Analysis 3.3). Two studies reported on both anxiety (Analysis 3.4) and insomnia (Analysis 3.5). In both cases, there were higher numbers of events in the bupropion plus varenicline arms; however, for anxiety, the lower CI did incorporate the null.

Five RCTs reported on SAEs for this comparison, although only four contributed to the pooled estimate due to a lack of events in one study. Evidence from 1268 participants resulted in imprecise evidence, with the 95% CI incorporating potential benefit and harm of bupropion plus varenicline, as well as no difference (RR 1.23, 95% CI 0.63 to 2.42; I^2^ = 0%; low‐certainty evidence; Analysis 3.6; Table 3). Johns 2017 did not provide sufficient data to include in the meta‐analysis but reported no difference in the frequency of SAEs reported between the bupropion plus varenicline group and the varenicline alone group.

Considering specific serious adverse events, no events were reported in our analyses of seizures or death by suicide (Analysis 3.7; Analysis 3.10). In the cases of overdoses (Analysis 3.8), suicide attempts (Analysis 3.9), and all‐cause mortality (Analysis 3.11), effect estimates were extremely imprecise, with CIs incorporating the potential for benefit and harm as well as no difference. In all three of these latter analyses, some of the studies reported no events.

##### Tolerability

Four RCTs, including 1230 participants, reported dropouts due to treatment for this comparison. The resulting point estimate favoured bupropion plus varenicline, but the CI also incorporated a potential benefit of varenicline alone (RR 0.80, 95% CI 0.45 to 1.45; I^2^ = 0%; low‐certainty evidence; Analysis 3.12; Table 3).

We carried out sensitivity analyses for all of the above harm and tolerability analyses for each comparison, removing studies at overall high risk of bias where this was relevant. In no cases did this change the interpretation of the effect. Additional sensitivity analyses, excluding studies with industry support, did not indicate that our findings were sensitive to the inclusion of these studies (see Table 4).

#### Effects of bupropion only across the above comparisons

We also carried out our analyses investigating harms and tolerability by grouping all of the above comparisons together, as they all isolated the effect of bupropion.

##### Harms

Across comparisons, there was evidence to suggest that taking bupropion increased the incidence of AEs relative to placebo or no pharmacological treatment, and in combination with NRT and varenicline in comparison to NRT and varenicline alone (RR 1.14, 95% CI 1.11 to 1.17; 27 studies, 12,313 participants; Analysis 4.1). However, substantial unexplained heterogeneity was detected between studies (I^2^ = 68%). A meta‐analysis of 10 studies found evidence to suggest bupropion increased the likelihood of developing psychiatric AEs, with CIs excluding no difference (RR 1.23, 95% CI 1.14 to 1.32; 5379 participants; Analysis 4.2). This effect is largely driven by Anthenelli 2016 (with an overall weighting of 80.0%); however, as we judged this study to be at low risk of bias, and the effects are consistent with those detected by the other studies included in the analysis (I^2^ = 34%), this was not deemed to be problematic. A meta‐analysis of 30 studies provided evidence that the use of bupropion may have increased the likelihood of SAEs (RR 1.17, 95% CI 0.93 to 1.47; I^2^ = 0%; 12,883 participants; Analysis 4.3). However, the CI encompassed no difference, as well as a clinically significant increase. There was insufficient evidence to determine whether bupropion use was associated with the likelihood of seizures (RR 2.93, 95% CI 0.74 to 11.54; I^2^ = 0%; 15 studies, 8092 participants; Analysis 4.4), overdoses (RR 1.13, 95% CI 0.21 to 5.99; I^2^ = 0%; 7 studies, 6135 participants; Analysis 4.5), suicide attempts (RR 0.88, 95% CI 0.25 to 3.14; I^2^ = 0%; 13 studies, 8027 participants; Analysis 4.6), death by suicide (RR 0.34, 95% CI 0.01 to 8.26; 16 studies, 10,036 participants; I^2^ = not applicable because only one study reported an event; Analysis 4.7), or all‐cause mortality (RR 0.81, 95% CI 0.42 to 1.58; I^2^ = 0%; 24 studies, 12,861 participants; Analysis 4.8). In all cases, the number of events reported was very low, which resulted in substantial imprecision and CIs encompassing both clinically significant benefit and harm.

However, there was evidence that those randomised to receive bupropion were more likely to report symptoms of anxiety (RR 1.45, 95% CI 1.26 to 1.67; I^2^ = 30%; 15 studies, 9123 participants; Analysis 4.9) and insomnia (RR 1.73, 95% CI 1.59 to 1.88; I^2^ = 8%; 25 studies, 12,132 participants; Analysis 4.10) at follow‐up.

##### Tolerability

There was evidence that the risk of dropouts due to AEs of the treatment was higher in groups receiving bupropion across comparisons (RR 1.42, 95% CI 1.25 to 1.60; I^2^ = 0%; 31 studies, 14,313 participants; Analysis 4.11). Our point estimate suggests that participants taking bupropion had a 25% to 60% increased risk of dropping out relative to controls. However, there were moderate subgroup differences detected (Chi^2^ = 3.97, degrees of freedom (df) = 2 (P = 0.14), I^2^ = 49.6%), with a potential difference in interpretation between the first two subgroups (bupropion versus placebo or no pharmacological treatment; bupropion plus NRT versus NRT alone) and the final subgroup (bupropion plus varenicline versus varenicline alone). There were considerably more trials and participants contributing data to the first subgroup than the second and third, and thus findings of this subgroup analysis must be treated with caution.

For bupropion versus placebo/no pharmacological treatment (RR 1.44, 95% CI 1.27 to 1.65; I^2^ = 2%; 25 studies, 12,346 participants; high‐certainty evidence; Analysis 1.14 and Analysis 4.11; Table 1) and bupropion plus NRT versus NRT alone (RR 1.67, 95% CI 0.95 to 2.92; I^2^ = 0%; 3 studies, 737 participants; low‐certainty evidence; Analysis 2.11 and Analysis 4.11; Table 2), the point estimate favoured placebo/no pharmacological treatment or NRT alone, respectively. In the latter subgroup, the CI crossed the line of no effect; however, this may have been due to higher levels of imprecision in this subgroup. In the bupropion plus varenicline versus varenicline alone subgroup, the point estimate favoured bupropion plus varenicline; however, imprecision was also high in this group, meaning that the CI encompassed both potential benefit and harm (RR 0.80, 95% CI 0.45 to 1.45; I^2^ = 0%; 4 studies, 1230 participants; low‐certainty evidence; Analysis 3.12 and Analysis 4.11; Table 3). 

#### Bupropion versus other pharmacotherapies

##### Smoking cessation

We found evidence to suggest that bupropion may be less effective than varenicline (RR 0.73, 95% CI 0.67 to 0.80; I^2^ = 0%; 9 studies, 7564 participants; Analysis 5.1) and combination NRT (i.e. a combination of nicotine patch and another form of NRT; RR 0.74, 95% CI 0.55 to 0.98; I^2^ = 0%; 2 studies, 720 participants; Analysis 6.1) for smoking cessation. However, there was no clear evidence of a difference in quit rates between bupropion and single‐form NRT (e.g. nicotine patch or gum alone; RR 1.03, 95% CI 0.93 to 1.13; I^2^ = 0%; 12 studies, 7613 participants; Analysis 6.1). In all cases, removing studies deemed to be at an overall high risk of bias did not change the interpretation of the effect estimates.

There was some evidence that bupropion may be more effective than nortriptyline in aiding smoking cessation (RR 1.30, 95% CI 0.93 to 1.82; I^2^ = 0%; 3 studies, 417 participants; Analysis 7.1). However, event rates were low (101 participants), and the result imprecise. This means it is possible that it will change as more evidence becomes available. The result was similar when we removed one study judged to be at high risk of bias from the analysis (Hall 2002).

###### Depression

One post hoc analysis found that bupropion was more effective than NRT in those with a history of depression (Stapleton 2013). See Table 5. Only two trials examined the interaction between depression and quit rates for bupropion and nortriptyline (Hall 2002; Wagena 2005). Both of the within‐study analyses found that participants classified as depressed were more likely to quit using bupropion than nortriptyline (Table 5).

##### Harms

There was evidence that randomisation to bupropion resulted in minimal difference in reporting of AEs when compared to both varenicline (RR 0.98, 95% CI 0.95 to 1.00; I^2^ = 0%; 6 studies, 4332 participants; Analysis 5.2) and NRT (RR 1.02, 95% CI 0.98 to 1.06; I^2^ = 0%; 4 studies, 4276 participants; Analysis 6.2). For SAEs, point estimates suggested more SAEs in the bupropion arms. However, there were far fewer events in these analyses, meaning they were underpowered, and we can have less certainty in their results (for varenicline: RR 1.39, 95% CI 0.94 to 2.04; I^2^ = 0%; 5 studies, 4920 participants; Analysis 5.3; for NRT: RR 1.22, 95% CI 0.83 to 1.80; I^2^ = 19%; 8 studies, 6035 participants; Analysis 6.6). When focusing on psychiatric AEs only, there was no clear evidence of a difference when comparing bupropion to varenicline (RR 1.07, 95% CI 0.99 to 1.16; I^2^ = 0%; 2 studies, 4051 participants; Analysis 5.4). For psychiatric AEs only, heterogeneity was so high when comparing bupropion to NRT that we deemed it inappropriate to present a pooled estimate (I^2^ = 89%; Analysis 6.3). There was insufficient evidence to indicate whether bupropion increased the risk of many of the other harm outcomes assessed (seizures, overdoses, suicide attempts, death by suicide, and all‐cause mortality) when compared to varenicline and NRT due to a paucity of relevant data. This means that when we could calculate estimates, these were extremely imprecise, with CIs encompassing both potential benefit and harm of the intervention (Analysis 5.7; Analysis 5.8; Analysis 5.9; Analysis 5.10; Analysis 5.11; Analysis 6.7; Analysis 6.8; Analysis 6.9; Analysis 6.10; Analysis 6.11).

We also found evidence that participants in the bupropion groups experienced more insomnia and anxiety than people in the varenicline groups (insomnia: RR 1.40, 95% CI 1.22 to 1.60; I^2^ = 9%; 3 studies, 5208 participants; Analysis 5.6; anxiety: RR 1.28, 95% CI 1.07 to 1.53; I^2^ = 0%; 2 studies, 4705 participants; Analysis 5.5) and the NRT groups (insomnia: RR 1.31, 95% CI 1.10 to 1.55; I^2^ = 47%; 2 studies, 4128 participants; Analysis 6.5; anxiety: RR 1.31, 95% CI 1.06 to 1.62; I^2^ = 67%; 2 studies, 4855 participants; Analysis 6.4) at follow‐up. However, we detected moderate heterogeneity for both the insomnia and anxiety outcomes for the comparison to NRT. When we carried out a sensitivity analysis, removing the study judged to be at high risk of bias for the anxiety outcome, the 95% CIs shifted to incorporate no between‐group difference in anxiety (RR 1.23, 95% CI 0.99 to 1.53; I^2^ = 13%; 1 study, 4028 participants).

We were unable to include two studies in the above meta‐analyses. Fatemi 2013 reported that they found no significant differences between bupropion and varenicline in regards to side effects. Varenicline specifically did not exhibit a significant impact on overall depression, psychopathology, suicidality, mania, akathisia, tardive dyskinesia, abnormal dreams, dizziness, insomnia, chest pain, dry mouth, heart rate and blood pressure, pain, confusion, or irritability. Johns 2017 found both bupropion and varenicline to be well tolerated, but participants in the varenicline group reported increased fatigue, digestive symptoms (e.g. nausea, diarrhoea), and sleep‐related concerns (e.g. abnormal dreams, insomnia). They reported no difference in the frequency of SAEs between the bupropion and varenicline groups.

There was insufficient evidence to determine whether bupropion increased the risk of any harms investigated in this review when compared to nortriptyline (Analysis 7.2; Analysis 7.3), or gabapentin – a drug used to treat epilepsy and nerve pain and not licensed for smoking cessation (Analysis 8.1). In the cases of Analysis 7.3 (bupropion versus nortriptyline; SAEs) and Analysis 8.1 (bupropion versus gabapentin; SAEs), it was not possible to generate pooled estimates due to a lack of studies; in the case of Analysis 7.2 (bupropion versus nortriptyline; insomnia outcome), statistical heterogeneity was so high that it was not appropriate to present a pooled estimate (I^2^ = 90%).

##### Tolerability

There was some evidence that bupropion may have led to an increase in trial dropouts due to treatment, when compared to varenicline (RR 1.18, 95% CI 1.00 to 1.39; I^2^ = 11%; 6 studies, 6111 participants; Analysis 5.12) or NRT (RR 1.14, 95% CI 0.95 to 1.38; I^2^ = 33%; 4 studies, 4825 participants; Analysis 6.12). However, in both cases, the findings were limited by imprecision, with the CIs encompassing as many or more dropouts in the comparator, respectively.

There was also insufficient evidence to determine whether bupropion increased the risk of trial dropouts due to treatment, when compared to both nortriptyline (Analysis 7.4) and gabapentin (Analysis 8.2), with imprecise estimates based on minimal data in both cases.

Where possible, for the above harm and tolerability outcomes, we carried out sensitivity analyses, removing studies judged to be at high risk of bias. In the rare cases where this was appropriate, there was no appreciable change in the interpretation of the effect estimates.

#### Bupropion at different doses

##### Smoking cessation

There was no clear evidence to indicate a differential effect between a 150 mg or 300 mg dose of bupropion on the likelihood of smoking cessation. Whilst the pooled estimate was 1.08 in favour of a 300 mg dose, the 95% CI encompassed a potential benefit of either dose (RR 1.08, 95% CI 0.93 to 1.26; I^2^ = 49%; 3 studies, 2042 participants; Analysis 9.1). Removing one study at high risk of bias did not change the clinical interpretation of the analysis.

###### Depression

None of the relevant included studies investigated a potential link between depression and quit rates.

##### Harms

We were unable to draw conclusions about any of the harm outcomes for this comparison. Analyses that could be carried out (SAEs, Analysis 9.4; overdoses, Analysis 9.5; suicide attempts, Analysis 9.6; death by suicide, Analysis 9.7; all‐cause mortality, Analysis 9.8; insomnia, Analysis 9.3; anxiety, Analysis 9.2), suffered from substantial imprecision due to a low number of events (ranging from 0 to 99 across the aforementioned analyses), and in all cases, 95% CIs encompassed one.

##### Tolerability

Our analysis of dropouts due to adverse event data also suffered from imprecision (Analysis 9.9), and we were unable to draw conclusions.

Sensitivity analyses removing studies judged to be at high risk of bias was not appropriate for harm and tolerability outcomes.

#### Bupropion of different durations

##### Smoking cessation

One study looked at the effect of bupropion (300 mg per day) when taken for seven versus 19 weeks (Rovina 2003). This study found higher quit rates in the 19‐week treatment (RR 1.45, 95% CI 1.04 to 2.03; 233 participants; Analysis 10.1).

###### Depression

Rovina 2003 did not investigate a potential link between depression and quit rates.

#### Nortriptyline versus placebo

##### Smoking cessation

Pooling six trials comparing nortriptyline to placebo showed evidence of benefit of nortriptyline over placebo for smoking cessation, with CIs excluding no difference (RR 2.03, 95% CI 1.48 to 2.78; I^2^ = 16%; 6 studies, 975 participants; Analysis 11.1). Removing two studies judged to be at high risk of bias did not influence the result (Hall 2002; Prochazka 1998).

###### Depression

One within‐study comparison found that a past history of depression did not appear to moderate the efficacy of nortriptyline, but subgroup numbers were small (Hall 1998). However, another within‐study analysis found that the most effective factor for ensuring the efficacy of nortriptyline was a negative history of depression (Da Costa 2002).

##### Harms

One study reported on anxiety (Analysis 11.2) and two on insomnia (Analysis 11.3). Both analyses found higher rates of these AEs in the nortriptyline arms than the placebo arms. However, the estimates were imprecise, with CIs incorporating both benefit and harm of the intervention.

One RCT reported on SAEs for this comparison. Of the 103 participants, none reported serious adverse events (Analysis 11.4).

##### Tolerability

Four RCTs, including 537 participants, reported dropouts due to treatment whilst comparing nortriptyline to placebo. The resulting point estimate favoured nortriptyline (RR 1.99, 95% CI 1.18 to 3.36; I^2^ = 23%; Analysis 11.5).

#### Nortriptyline plus nicotine replacement therapy (NRT) versus NRT alone

##### Smoking cessation

Pooling four trials using nortriptyline as an adjunct to nicotine patch therapy (Aveyard 2008; Hall 2004; Prochazka 2004; Richmond 2013), found a potential benefit of combination nortriptyline and NRT for smoking cessation relative to NRT alone (RR 1.21, 95% CI 0.94 to 1.55; I^2^ = 26%; 4 studies, 1644 participants; Analysis 12.1). However, there was imprecision around the effect estimate, with the CIs encompassing both no difference and a clinically significant benefit. The interpretation of the result remained the same when we removed studies judged to be at an overall high risk of bias.

###### Depression

One study comparing nortriptyline plus NRT to NRT alone found no evidence supporting depression as a moderator of abstinence in either the combination nortriptyline and NRT or the placebo arms of the trial (Aveyard 2008).

##### Harms

One study reported on insomnia with 158 participants, resulting in CIs incorporating both benefit and harm of the nortriptyline and NRT versus NRT alone (Analysis 12.2).

No studies reported on SAEs for this comparison.

##### Tolerability

One RCT, including 158 participants, reported dropouts due to treatment for this comparison. The resulting point estimate favoured the NRT‐only arm (RR 10.00, 95% CI 1.31 to 76.28; Analysis 12.3), with substantial imprecision due to very few events across arms (n = 11).

#### Effects of nortriptyline only across the above comparisons

##### Harms

As for bupropion, we combined the above two nortriptyline comparisons to investigate harms and tolerability with increased statistical power. As reported above, only one trial investigated the likelihood of SAEs across comparisons, with no SAEs reported in either trial arm (Haggsträm 2006; Analysis 13.1). The insomnia and anxiety outcomes provided insufficient evidence to draw conclusions for this comparison (Analysis 13.3; Analysis 13.2).

##### Tolerability

Our meta‐analysis including five studies investigating the isolated effect of nortriptyline found evidence that dropout due to treatment was over twice as likely when randomised to nortriptyline (RR 2.40, 95% CI 1.45 to 3.96; I^2^ = 37%; 5 studies, 695 participants; Analysis 13.4). However, the magnitude of the effect should be treated with caution due to imprecision.

#### Other antidepressant monotherapies versus control

##### Smoking cessation

We did not find clear evidence to indicate that selective serotonin reuptake inhibitors (SSRIs) increased the likelihood of smoking cessation relative to control (RR 0.93, 95% CI 0.71 to 1.22; I^2^ = 0%; 4 studies, 1594 participants; Analysis 14.1). However, there was a low number of events across studies (193 participants) which should be taken into account. We subgrouped our meta‐analysis by the type of SSRI used in the trial (two studies on fluoxetine: Niaura 2002; Spring 2007; one study on paroxetine: Killen 2000; one study on sertraline: Covey 2002), and found no clear evidence of a subgroup difference (I^2^ = 0%). This should be treated with caution due to the low number of studies in each group.

We found some indication that monoamine oxidase inhibitors (MAOIs) may increase the likelihood of smoking cessation relative to control (RR 1.29, 95% CI 0.93 to 1.79; I^2^ = 0%; 6 studies, 827 participants; Analysis 16.1). However, event rates were low (193 participants) resulting in imprecision, and CIs encompassed the possibility of harm. There was no effect of removing the one study deemed to be at high risk of bias (George 2003). Our meta‐analysis included one trial of moclobemide (Berlin 1995), and five of selegiline (Biberman 2003; George 2003; Kahn 2012; Killen 2010; Weinberger 2010), and we subgrouped these accordingly. We did not identify any evidence of a subgroup difference (I^2^ = 0%), but this should be treated with caution as only a single study contributed to the moclobemide group.

One trial of venlafaxine did not show conclusive evidence of increased smoking cessation compared to placebo (Cinciripini 2005) (RR 1.22, 95% CI 0.64 to 2.32; 1 study, 147 participants; Analysis 17.1). Although the effect estimate was in favour of venlafaxine, CIs were wide and encompassed both potential benefit and harm.

Two small trials comparing St John’s wort to placebo provided no clear evidence to suggest it was a better smoking cessation treatment than placebo when pooled (Parsons 2009; Sood 2010) (RR 0.81, 95% CI 0.26 to 2.53; I^2^ = 29%; 2 studies, 261 participants; Analysis 18.1). However, there was substantial imprecision.

The one trial assessing S‐adenosyl‐L‐methionine (SAMe) compared to placebo provided no evidence of a benefit for smoking cessation (RR 0.70, CI 0.24 to 2.07; 1 study, 120 participants; Analysis 19.1). However, the number of included participants and the number of events were small.

###### Depression

Of the three studies conducting post hoc analyses of fluoxetine (Saules 2004; Spring 2007) and paroxetine (Killen 2000) to assess the interaction between depression and antidepressant quit rates, none provided evidence to support this interaction (George 2003; Kahn 2012).

The two studies conducting post hoc analyses of selegiline to assess the interaction between depression and antidepressant quit rates also did not provide evidence to support this interaction (George 2003; Kahn 2012). 

##### Harms

There was insufficient evidence to indicate whether SSRIs increased the risk of AEs relative to placebo. Only two trials of fluoxetine investigated this, one of which provided data that could be analysed (NCT00578669; Analysis 14.2). Minami 2014 found that those in the fluoxetine group reported more symptoms the day before the set quit date, in comparison to those in the placebo group. Further, failure to quit on the target quit date could be significantly predicted by levels of side effects.

For the comparison of MAOIs relative to placebo, there was no clear evidence of increased risk of experiencing either AEs (Analysis 16.2), psychiatric AEs (Analysis 16.3), or SAEs (Analysis 16.6). However, the latter two analyses suffered from substantial imprecision and should be treated with caution. Substantial imprecision and heterogeneity also meant that we were unable to draw conclusions regarding insomnia and anxiety (Analysis 16.4; Analysis 16.5).

The one study assessing harm outcomes for St John's wort versus placebo did not provide sufficient evidence to assess whether it increased the likelihood of SAEs or all‐cause mortality specifically (Parsons 2009; Analysis 18.2; Analysis 18.3). A study of SAMe versus placebo did not provide sufficient evidence on AEs or insomnia (Sood 2012; Analysis 19.2; Analysis 19.3).

##### Tolerability

There was also evidence to suggest SSRIs may increase the likelihood of dropout due to treatment (RR 2.59, 95% CI 1.70 to 3.94; I^2^ = 0%; 3 studies, 1270 participants; Analysis 14.3). When the four included studies were subgrouped into two of fluoxetine (Niaura 2002; Spring 2007), and one of sertraline (Covey 2002), there was no evidence of a subgroup difference (I^2^ = 0%).

There was some evidence that there may be an increased risk of discontinuation in the MAOI groups, and this persisted when we removed one study judged to be at high risk of bias. However, there was substantial imprecision in this analysis (Analysis 16.7).

One study each assessed dropout due to treatment for venlafaxine versus placebo (Cinciripini 2005; Analysis 17.2), St John's wort versus placebo (Parsons 2009; Analysis 18.4), and SAMe versus placebo (Sood 2012; Analysis 19.4). These studies did not provide sufficient evidence to draw clear conclusions.

#### Other antidepressant combination therapies versus control

##### Smoking cessation

Three trials evaluated fluoxetine as an adjunct to NRT (Blondal 1999; Brown 2014; Saules 2004), but also did not provide evidence of an increased likelihood of smoking cessation relative to NRT alone when pooled (RR 0.70, 95% CI 0.48 to 1.03; I^2^ = 0%; 3 studies, 466 participants; Analysis 15.1). Again, interpretation did not change when we removed studies judged to be at high risk of bias, and there was evidence of imprecision. In this instance, CIs encompassed the possibility of no difference and a clinically significant harm.

###### Depression

None of these studies investigated depression as a moderator of abstinence.

##### Harms

There was insufficient evidence from one study investigating the effect of selegiline plus NRT versus NRT alone on SAEs (Biberman 2003; Analysis 20.1). Similarly, Berlin 2012 alone provides insufficient data to assess the effects of EVT302 plus NRT versus NRT alone on AEs (Analysis 21.1) and SAEs (Analysis 21.2).

##### Tolerability

There was insufficient information available about dropout due to treatment from the one study comparing selegiline plus NRT versus NRT alone (Biberman 2003; Analysis 20.2), and the one study comparing EVT302 plus NRT versus NRT alone (Berlin 2012; Analysis 21.3), to draw conclusions.

#### Other antidepressants at different doses

##### Smoking cessation

We were unable to evaluate the efficacy of 300 mg versus 600 mg of St John's wort, as the one trial comparing these differences had too small a sample size (28 participants), with no individuals abstinent from smoking at 12 months' follow‐up (Barnes 2006). One study compared the efficacy of 30 mg versus 60 mg of fluoxetine, and found the same quit rates in both groups (RR 1.00, 95% CI 0.63 to 1.59; 656 participants; Analysis 22.1). However, this result should be treated with caution due to imprecision.

###### Depression

These studies did not investigate depression as a moderator of smoking quit rates.

##### Harms

Berlin 2002 only followed up participants to eight weeks, and therefore we did not use efficacy data from this study. However, they reported data on harms. The study compared 100 mg with 200 mg daily doses of lazabemide. No SAEs were recorded during the trial (Analysis 23.1). There was insufficient evidence to conclude whether participants randomised to the higher dose were more likely to suffer from symptoms of insomnia (Analysis 23.3) or anxiety (Analysis 23.2).

Due to the very small sample size of Barnes 2006, there was insufficient evidence to assess the likelihood of AEs in participants receiving a 300 mg daily dose of St. John's wort versus a 600 mg dose (28 participants; Analysis 24.2). Similarly, there was insufficient evidence investigating the effect of a 800 mg daily dose of SAMe versus a 1600 mg daily dose on the risk of AEs (Sood 2012; Analysis 25.1).

##### Tolerability

Niaura 2002 found some evidence that a 60 mg daily dose of fluoxetine compared to a 30 mg dose daily increased the likelihood of trial discontinuation due to treatment (RR 0.64, 95% CI 0.46 to 0.87; 1 study, 656 participants; Analysis 22.2). However, there was insufficient evidence to conclude whether participants randomised to the higher 200 mg dose of lazabemide were more likely to drop out of the trial due to the medication than participants randomised to the lower 100 mg dose (Berlin 2002; Analysis 23.4), or whether participants randomised to a 1600 mg dose of SAMe were more likely to drop out than those randomised to a 800 mg dose (Sood 2012; Analysis 25.2).

## Discussion

### Summary of main results

This updated review summarises and evaluates the evidence investigating the efficacy, harms, and tolerability of different types of antidepressant for smoking cessation. This review includes a total of 124 studies involving 48,832 participants, adding 10 new studies to the previous review version (Howes 2020). A total of 50 trials of 18,577 participants provide a large, high‐certainty evidence base confirming the benefit of bupropion used as a single pharmacotherapy for smoking cessation (Table 1). The pooled estimate suggests that bupropion increased long‐term quitting success by 49% to 72% when compared with placebo or no pharmacological treatment. Treatment effects appeared to be comparable across the range of populations, settings, and types of behavioural support studied, including those with and without a past history of depression. Our review finds insufficient evidence of a benefit of adding bupropion to nicotine replacement therapy (NRT) when compared to NRT alone (low‐certainty evidence; Table 2), although there was some indication that adding bupropion to varenicline treatment may result in higher quit rates than when compared to varenicline alone (moderate‐certainty evidence; Table 3). There is evidence to suggest that bupropion increases the risk of non‐serious adverse events (AEs), including psychiatric AEs, when compared to placebo or no pharmacological treatment. However, rates of SAEs were low and estimates incorporated the potential of no difference in the number of SAEs reported between arms (moderate‐certainty evidence; Table 1). There was, however, high‐certainty evidence that more people dropped out due to treatment when using bupropion than when using placebo or no pharmacological treatment (Table 1). Although non‐serious AEs were also found to be higher in the combination treatment arms (i.e. bupropion plus NRT when compared to NRT alone and bupropion plus varenicline when compared to varenicline alone), results for SAEs and rates of dropouts were inconclusive (low‐certainty evidence in all cases; Table 2; Table 3).

The evidence suggests that people using bupropion may be more likely to quit than those using nortriptyline, but does not clearly suggest evidence of a difference in the efficacy of bupropion versus single‐form NRT. Participants taking bupropion may be less likely to quit than those treated with varenicline, and combined NRT. The evidence relating to the harms of treatment is inconclusive when comparing bupropion to NRT, varenicline, and nortriptyline, due to a paucity of studies, overall participants, and events. While our review did not specifically consider tobacco users with severe mental illness, subgroup analysis of a large included trial indicates the benefits of bupropion, NRT, and varenicline, with no treatment‐related increase in neuropsychiatric adverse events in this cohort (Anthenelli 2016; Evins 2021).

We found evidence that nortriptyline is also an effective agent to aid smoking cessation when compared with placebo, based on a meta‐analysis of six studies, including 975 participants. However, there is no clear evidence that other antidepressants, including selective serotonin reuptake inhibitors (SSRIs), monoamine oxidase inhibitors (MAOIs), venlafaxine, St John's wort, and S‐adenosyl‐L‐methionine (SAMe), are effective as cessation treatments. Therefore, despite SSRIs being commonly used to treat depression, there does not seem to be any justification for continuing to pursue their use for smoking cessation, where other more clearly effective options exist.

Few studies examined whether current or previous depression moderated the effectiveness of antidepressants to aid smoking cessation. Those comparing bupropion to placebo or no pharmacological treatment found no evidence of an interaction between depression and use of bupropion. Studies contributing to other comparisons found varied but uncertain results.

### Overall completeness and applicability of evidence

The searches conducted for this study were broad and identified any studies where a drug was described as being an 'antidepressant' or 'antidepressive'. In cases where we were unsure of whether a medication was classed as an antidepressant, we conducted a brief literature search to clarify whether they had been used in other research as antidepressants, to ensure we included all relevant medications. We also searched trial registers to identify any ongoing or completed but unpublished, registered studies assessing the efficacy and harms of antidepressants for smoking cessation.

Most studies included in this review recruited adult smokers who were typically motivated to quit; four studies recruited young people aged between 12 and 21 years. Of the study populations included in our review, the lowest mean cigarettes smoked per day was 10, and the highest was 44, meaning that most studies included participants with significant tobacco addiction. These results may not apply to populations with few symptoms of tobacco addiction. In addition, few studies specifically recruited participants with mental health disorders; one was weighted particularly heavily in the meta‐analysis (Anthenelli 2016). Anthenelli 2016 recruited a subset of participants with mental health disorders, who were described as 'clinically stable', suggesting that they may not be entirely representative of the wider population diagnosed with a mental health disorder. Further studies are needed amongst those with depression to provide greater confidence in our findings, which suggest that bupropion is as effective for smoking cessation in people with a mental health diagnosis as those without.

### Certainty of the evidence

Of the 124 studies included in this review, we judged 14 to be at low risk of bias for all domains, and 34 to be at high risk in one or more domains. We judged the remaining 76 studies to be at an unclear risk due to a lack of reporting of key information. In these cases, it is impossible to know whether these studies were at any risk of bias or whether the information was simply not reported. To investigate the potential impact on results of studies that we judged to be at high risk of bias, we carried out sensitivity analyses, removing studies judged to be at high risk from analyses and observing the effects on results (where this was possible). In most cases, this had no effect on the clinical interpretation of the analyses.

We assessed the certainty of the evidence by creating three summary of findings tables (Table 1; Table 2; Table 3) and carrying out GRADE ratings for three comparisons (bupropion versus placebo/no pharmacological treatment; bupropion plus NRT versus NRT alone; bupropion plus varenicline versus varenicline alone) (Schünemann 2013). The efficacy of bupropion versus placebo/no pharmacological treatment for smoking cessation generated high‐certainty evidence. We judged combination bupropion and varenicline evidence to be of moderate certainty, whilst we judged the combination bupropion and NRT to be of low‐certainty evidence. We judged the SAE outcome as moderate certainty for bupropion versus placebo/no pharmacological treatment, and low certainty for both bupropion plus NRT versus NRT alone and bupropion plus varenicline versus varenicline alone. The evidence on dropout due to treatment was deemed to be high certainty for bupropion versus placebo/no pharmacological treatment, and again low certainty for both bupropion plus NRT versus NRT alone and bupropion plus varenicline versus varenicline alone. The reasons for downgrading the evidence were imprecision (confidence intervals (CIs) encompassing no difference, as well as the potential for both benefit and harm), and inconsistency (moderate heterogeneity detected in an analysis).

### Potential biases in the review process

We consider the review process used to be robust, and do not believe we have introduced any biases. However, Cochrane guidance to screen all reference lists of included studies and relevant reviews has not been followed for all updates of this review.

For outcome assessment, we followed the standard methods used for Cochrane Tobacco Addiction Group cessation reviews. Our search of the Cochrane Tobacco Addiction Specialised Register allowed us to capture two new ongoing studies (resulting in a total of four ongoing studies). However, there may be unpublished data that our searches did not uncover.

We generated and interpreted funnel plots for all analyses that included 10 or more studies that were also eligible for inclusion in our summary of findings table (i.e. the most clinically important). None of the funnel plots appeared to demonstrate evidence of publication bias (Figure 3; Figure 4; Figure 5). However, in the cases of Figure 4 and Figure 5, relatively few studies contributed to these plots (17 and 15, respectively), so these should be interpreted with caution. We also tested whether the inclusion of studies funded by the pharmaceutical industry or where a pharmaceutical company had supplied the medication for the study impacted the pooled results of our analyses. In no case did there appear to be any clear evidence of this (Table 4).

**3:**
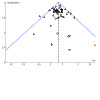
Funnel plot of comparison: 1 Bupropion versus placebo/no pharmacological treatment, outcome: 1.1 Smoking cessation

**4:**
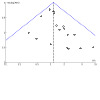
Funnel plot of comparison: 1 Bupropion versus placebo/no pharmacological treatment, outcome: 1.8 Serious adverse events

**5:**
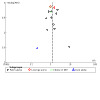
Funnel plot of comparison: 2 Bupropion and NRT versus NRT alone, outcome: 2.1 Smoking cessation

We considered participants lost to follow‐up as smokers, which is the current best practice in this field (West 2005). The Cochrane Tobacco Addiction Group policy is to present effect estimates as risk ratios (RRs), as these are easier to interpret than odds ratios (ORs). However, this means that where there are no events measured in both comparison groups, RRs cannot be calculated, and therefore do not contribute to the meta‐analysis. We considered alternative statistical approaches to deal with this but concluded that other approaches would be more difficult to interpret and that overall conclusions would not change as a result.

### Agreements and disagreements with other studies or reviews

The findings of this review align with the conclusions of other reviews and guidelines in a variety of populations (Cahill 2013; Hughes 2005; McRobbie 2005; Mills 2006, Tsoi 2010). The most recent US Preventative Task Force 2021 recommendation statements for pharmacotherapy state that bupropion is an effective smoking cessation treatment, and that varenicline appears to be more effective than bupropion or NRT (Patnode 2021). In the UK, bupropion, NRT, and varenicline (when available) are recommended for people over the age of 18 (NICE 2021). Open uncontrolled trials and observational studies of bupropion have shown real‐life quit rates comparable to those found in the clinical trials included in this review (Paluck 2006; Wilkes 2005). In addition, our findings regarding the beneficial effect of bupropion for smoking cessation, specifically in smokers living with mental illness, are consistent with a subset from a separate Cochrane Review evaluating smoking cessation treatments exclusively in populations with current or past depression (Van der Meer 2013).

However, our findings on the effectiveness of bupropion as an adjunct to NRT differ from the results of the United States Public Health Service (USPHS) clinical practice guideline (Fiore 2008). Whereas we did not detect clear evidence of a difference in efficacy when bupropion and NRT were used together compared to NRT alone, the USA guideline reported an OR of 1.30 (95% CI 1.0 to 1.80) favouring combination therapy (Fiore 2008, Table 6.28). The difference in meta‐analytic outcomes may be because our analysis included several studies of hard‐to‐treat populations not included in the USPHS analysis. Also, it could be because our analysis was a collation of 15 direct, within‐study randomised comparisons, whereas the USPHS carried out an indirect across‐study comparison of the results from the combination arms of three trials and the patch‐alone arms of 32 studies.

Thomas 2022 and Cahill 2013 used both direct and indirect statistical comparisons to compare the efficacy of bupropion to NRT and varenicline, using network meta‐analysis. The effect estimates generated resulted in similar conclusions to the ones drawn here; namely, bupropion and single‐form NRT resulted in similar quit rates and varenicline and combination NRT resulted in higher quit rates than bupropion.

Similar to our findings, other studies and systematic reviews looking at the SAE profile of bupropion are subject to uncertainty (Cahill 2013; Grandi 2011; Wightman 2010). However, the recent network meta‐analysis, Thomas 2022, found that, unlike NRT and varenicline, only bupropion at a standard dose increased the odds of SAEs compared to placebo. Our review also found moderate‐certainty evidence that SAEs may increase as a result of taking bupropion but this may change as more evidence becomes available. Our review did not find conclusive evidence that bupropion significantly increases the incidence of seizures due to substantial imprecision (RR 2.93, 95% CI 0.74 to 11.54). Due to the very low number of events (n = 7), the point estimate indicated a rate of 0 events per 1000 people taking bupropion, compared to a rate of 1 per 1000 cases presented elsewhere (Cahill 2013).

In contrast to the findings of the very large‐scale EAGLES trial (Anthenelli 2016), we have concluded that bupropion significantly increases psychiatric AEs. Whilst Anthenelli 2016 contributes over 95% of psychiatric AE data to our meta‐analysis, it concluded that bupropion does not significantly increase the incidence of psychiatric AEs. This discrepancy may be the result of including psychiatric AEs of any severity in our relevant meta‐analysis, whereas Anthenelli 2016 used a composite measure of only moderate‐ and severe‐intensity psychiatric events for their primary analysis. We cannot establish whether we would find the same if we were only to include moderate and severe psychiatric events, as study reporting does not allow us to discriminate between these events according to severity.

Taking into account (1) the combined evidence from this review, Cahill 2013, and Thomas 2022, suggesting that varenicline and combination NRT are more efficacious than bupropion; (2) evidence from Livingstone‐Banks 2023 suggesting that psychiatric AEs are not increased by varenicline; and (3) evidence from Hartmann‐Boyce 2018 and Theodoulou 2023 raising a lack of concerns around harms emerging from the use of NRT, varenicline (where available) or combination NRT may be more suitable options for people who wish to use a medication to quit smoking, especially those with mental health disorders.

## Authors' conclusions

**Implications for practice**

- Bupropion and nortriptyline are effective pharmacological treatments for smoking cessation. There is limited evidence that bupropion may be more effective than nortriptyline. Bupropion increases the rate of long‐term quitting by approximately 49% to 72%, and this effect appears to be stable regardless of the amount of behavioural support provided, and whether participants have current or a history of mental health disorders.
- Bupropion may cause an increase in adverse events (AEs), and specifically psychiatric AEs, leading to discontinuation of drug use in some users (approximately 9%). There is also evidence that bupropion may cause an increase in serious adverse events (SAEs; i.e. events that result in hospitalisation, disability, or death). However, estimates include the possibility of no difference as well as a potential 1% increase versus no use of bupropion.
- There is some evidence, limited by imprecision, that combining bupropion with varenicline may result in greater quit rates than using varenicline alone. However, there is less certain evidence of higher quit rates when combining bupropion with nicotine replacement therapy (NRT) relative to NRT alone, although this may change as more evidence becomes available.
- There is some evidence that bupropion is as effective as single‐form nicotine replacement therapy (NRT) for smoking cessation; however, it appears to be less effective than combination NRT (i.e. a patch combined with another form).
- There is a paucity of data investigating the efficacy and harms of antidepressants other than bupropion for smoking cessation. However, there are sufficient data to show that, in the light of the effectiveness of other medications, selective serotonin reuptake inhibitors (SSRIs) offer no worthwhile increase in smoking cessation rates.
- The evidence is insufficient to draw conclusions about whether existing depression modifies the efficacy of antidepressants for smoking cessation.

**Implications for research**

There is high‐certainty evidence that bupropion increases quit rates at six months or longer in adults motivated to quit. We consider that further research is highly unlikely to change our confidence in the efficacy of bupropion in this population. However, further studies could increase our confidence in the likelihood of SAEs and dropouts due to treatment. Any future studies comparing bupropion to placebo should ensure these outcomes are recorded and reported in detail.

- More studies assessing the efficacy and harms of different doses of bupropion, as well as doses higher than 300 mg, would clarify the most effective bupropion dosing strategy.
- More high‐quality studies are needed to assess the efficacy of bupropion when combined with varenicline treatment or NRT treatment.
- More high‐quality studies are needed to assess whether bupropion is particularly efficacious for supporting smoking cessation in people with depression.
- New studies of any antidepressant used as a treatment for smoking cessation should ensure that they measure and report on the number of participants experiencing SAEs and AEs, as well as reporting on the number of dropouts due to treatment. These numbers should be reported separately by study arm, as well as overall. Specifically, studies of bupropion should report on numbers of psychiatric AEs and provide more detail on the severity of these events.

## What's new

**Table:** 

**Date**	**Event**	**Description**
24 May 2023	New search has been performed	Updated search to April 2022.
24 May 2023	New citation required but conclusions have not changed	10 new included studies identified and study data added to existing comparators. Main conclusions remain unchanged with some rewording of secondary conclusions.

## History

Protocol first published: Issue 3, 1997 Review first published: Issue 3, 1997

**Table:** 

**Date**	**Event**	**Description**
4 May 2021	Amended	Minor corrections to figures in Summary of Findings tables (no changes to interpretation)
24 January 2020	New search has been performed	33 new included studies identified and study data added to existing comparators
24 January 2020	New citation required but conclusions have not changed	33 new included studies; additional safety analyses added. Main conclusions remain unchanged
14 June 2016	Amended	Corrected typographical error in Abstract results. Risk ratio for buproprion + NRT (12 trials) changed from 1.9 to 1.19. Now matches meta‐analysis 1.5
8 October 2013	New citation required but conclusions have not changed	Conclusions largely unchanged. Efficacy findings unchanged
8 October 2013	New search has been performed	Updated with 24 new included studies. Studies of S‐Adenosyl‐L‐Methionine and St John's wort included for the first time. Meta‐analyses of serious adverse events added
22 June 2011	Amended	Additional table converted to appendix to correct pdf format
5 October 2009	Amended	Correction to excluded studies table, detail added to Carrão 2007
30 July 2009	New search has been performed	Updated with 13 new included trials including 3 of selegiline, not previously covered. No substantial change to effects; main conclusions not altered
17 June 2008	Amended	Converted to new review format
11 October 2006	New citation required but conclusions have not changed	Seventeen new trials were added to the review for Issue 1, 2007. There were no major changes to the reviewers' conclusions.
16 July 2004	New citation required but conclusions have not changed	New trials of bupropion, nortriptyline and fluoxetine were added for Issue 4, 2004, and additional information on adverse effects was included. There were no major changes to the reviewers' conclusions.
8 January 2003	New citation required but conclusions have not changed	New trials of bupropion and nortriptyline were added to the review in Issue 2, 2003. There were no major changes to the reviewers' conclusions.
19 September 2001	New citation required but conclusions have not changed	Four new studies on bupropion, and one each on nortriptyline and paroxetine were added to the review in Issue 1, 2002. In press data from a trial of fluoxetine are included which differ from unpublished data previously used. The reviewers' conclusions about the efficacy of bupropion and nortriptyline were not changed substantively.
28 August 2000	New citation required and conclusions have changed	Updates the earlier Cochrane Review 'Anxiolytics and antidepressants for smoking cessation'. Anxiolytics are evaluated in a separate review.

## Notes

This review was first published as part of the review 'Anxiolytics and antidepressants for smoking cessation.' From Issue 4, 2000 the classes of drugs were reviewed separately.

## Appendices

### Appendix 1. Cochrane Tobacco Addiction Group (TAG) Specialised Register search strategy

Searched using CRS web on 29 April 2022

#1 (bupropion or zyban):TI,AB,MH,EMT,KY,XKY

#2 nortriptyline:TI,AB,MH,EMT,KY,XKY

#3 (monoamine oxidase inhib*):TI,AB,MH,EMT,KY,XKY

#4 (moclobemide or selegiline or lazabemide):TI,AB,MH,EMT,KY,XKY

#5 (SSRI* or ((selective serotonin re‐?uptake inhibitor*) or (selective serotonin reuptake inhibitor*))):TI,AB,MH,EMT,KY,XKY

#6 (fluoxetine or sertraline or paroxetine or zimelidine):TI,AB,MH,EMT,KY,XKY

#7 (doxepin or imipramine or tryptophan or venlafaxine):TI,AB,MH,EMT,KY,XKY

#8 ((john*?s wort) or hypericum):TI,AB,MH,EMT,KY,XKY

#9 #1 OR #2 OR #3 OR #4 OR #5 OR #6 OR #7 OR #8

(MH, EMT, KY and XKY are keyword fields)

## Data and analyses

**Comparison 1:** Bupropion versus placebo/no pharmacological treatment

Outcome or subgroup title	No. of studies	No. of participants	Statistical method	Effect size
1.1 Smoking cessation	50	18577	Risk Ratio (M‐H, Fixed, 95% CI)	1.60 [1.49, 1.72]
1.2 Smoking cessation ‐ subgroup by level of behavioural support	49	18498	Risk Ratio (M‐H, Fixed, 95% CI)	1.61 [1.50, 1.73]
1.2.1 Multisession group behavioural support	10	2001	Risk Ratio (M‐H, Fixed, 95% CI)	1.76 [1.44, 2.16]
1.2.2 Multisession individual counselling	32	15316	Risk Ratio (M‐H, Fixed, 95% CI)	1.61 [1.49, 1.75]
1.2.3 Low‐intensity support	2	97	Risk Ratio (M‐H, Fixed, 95% CI)	1.48 [0.44, 4.93]
1.2.4 Not specified	5	1084	Risk Ratio (M‐H, Fixed, 95% CI)	1.39 [1.13, 1.71]
1.3 Smoking cessation ‐ subgroup by mental health disorders	49	18498	Risk Ratio (M‐H, Fixed, 95% CI)	1.61 [1.50, 1.73]
1.3.1 Psychiatric conditions	5	2180	Risk Ratio (M‐H, Fixed, 95% CI)	1.67 [1.30, 2.15]
1.3.2 Non‐psychiatric	45	16318	Risk Ratio (M‐H, Fixed, 95% CI)	1.60 [1.49, 1.73]
1.4 Adverse events	21	10931	Risk Ratio (M‐H, Fixed, 95% CI)	1.14 [1.11, 1.18]
1.5 Psychiatric adverse events	8	4494	Risk Ratio (M‐H, Fixed, 95% CI)	1.25 [1.15, 1.36]
1.6 Anxiety	11	7406	Risk Ratio (M‐H, Fixed, 95% CI)	1.42 [1.21, 1.67]
1.7 Insomnia	22	11077	Risk Ratio (M‐H, Fixed, 95% CI)	1.78 [1.62, 1.96]
1.8 Serious adverse events	23	10958	Risk Ratio (M‐H, Fixed, 95% CI)	1.16 [0.90, 1.48]
1.9 Seizures	13	7344	Risk Ratio (M‐H, Fixed, 95% CI)	2.93 [0.64, 13.37]
1.10 Overdoses	5	5585	Risk Ratio (M‐H, Fixed, 95% CI)	2.15 [0.23, 19.86]
1.11 Suicide attempts	10	6484	Risk Ratio (M‐H, Fixed, 95% CI)	1.62 [0.29, 8.92]
1.12 Death by suicide	14	8822	Risk Ratio (M‐H, Fixed, 95% CI)	0.34 [0.01, 8.26]
1.13 All‐cause mortality	21	11403	Risk Ratio (M‐H, Fixed, 95% CI)	0.89 [0.42, 1.87]
1.14 Dropouts due to treatment	25	12346	Risk Ratio (M‐H, Fixed, 95% CI)	1.44 [1.27, 1.65]

**Comparison 2:** Bupropion plus nicotine replacement therapy (NRT) versus NRT alone

Outcome or subgroup title	No. of studies	No. of participants	Statistical method	Effect size
2.1 Smoking cessation	15	4117	Risk Ratio (M‐H, Random, 95% CI)	1.17 [0.95, 1.44]
2.1.1 Patch alone	10	1835	Risk Ratio (M‐H, Random, 95% CI)	1.25 [0.87, 1.81]
2.1.2 Lozenge alone	2	1051	Risk Ratio (M‐H, Random, 95% CI)	1.21 [0.81, 1.81]
2.1.3 Choice of NRT	2	1181	Risk Ratio (M‐H, Random, 95% CI)	0.98 [0.76, 1.26]
2.1.4 Gum alone	1	50	Risk Ratio (M‐H, Random, 95% CI)	0.20 [0.01, 3.97]
2.2 Adverse events	3	339	Risk Ratio (M‐H, Fixed, 95% CI)	1.21 [1.03, 1.43]
2.3 Psychiatric adverse events	1		Risk Ratio (M‐H, Fixed, 95% CI)	Totals not selected
2.4 Anxiety	3	1218	Risk Ratio (M‐H, Fixed, 95% CI)	1.58 [0.97, 2.56]
2.5 Insomnia	2	556	Risk Ratio (M‐H, Fixed, 95% CI)	1.55 [1.24, 1.93]
2.6 Serious adverse events	4	657	Risk Ratio (M‐H, Fixed, 95% CI)	1.52 [0.26, 8.89]
2.7 Seizures	1	527	Risk Ratio (M‐H, Fixed, 95% CI)	2.92 [0.12, 71.39]
2.8 Suicide attempts	1	0	Risk Ratio (M‐H, Fixed, 95% CI)	Not estimable
2.9 Death by suicide	1	0	Risk Ratio (M‐H, Fixed, 95% CI)	Not estimable
2.10 All‐cause mortality	2	731	Risk Ratio (M‐H, Fixed, 95% CI)	0.68 [0.12, 3.98]
2.11 Dropouts due to treatment	3	737	Risk Ratio (M‐H, Fixed, 95% CI)	1.67 [0.95, 2.92]

**Comparison 3:** Bupropion plus varenicline versus varenicline alone

Outcome or subgroup title	No. of studies	No. of participants	Statistical method	Effect size
3.1 Smoking cessation	3	1057	Risk Ratio (M‐H, Random, 95% CI)	1.21 [0.95, 1.55]
3.2 Adverse events	4	1043	Risk Ratio (M‐H, Fixed, 95% CI)	1.09 [1.02, 1.17]
3.3 Psychiatric adverse events	2	835	Risk Ratio (M‐H, Fixed, 95% CI)	1.15 [1.03, 1.30]
3.4 Anxiety	2	499	Risk Ratio (M‐H, Fixed, 95% CI)	1.55 [1.01, 2.38]
3.5 Insomnia	2	499	Risk Ratio (M‐H, Fixed, 95% CI)	1.45 [1.14, 1.84]
3.6 Serious adverse events	5	1268	Risk Ratio (M‐H, Fixed, 95% CI)	1.23 [0.63, 2.42]
3.7 Seizures	1	0	Risk Ratio (M‐H, Fixed, 95% CI)	Not estimable
3.8 Overdoses	2	550	Risk Ratio (M‐H, Fixed, 95% CI)	0.34 [0.01, 8.27]
3.9 Suicide attempts	3	1056	Risk Ratio (M‐H, Fixed, 95% CI)	0.34 [0.04, 3.27]
3.10 Death by suicide	2	0	Risk Ratio (M‐H, Fixed, 95% CI)	Not estimable
3.11 All‐cause mortality	2	727	Risk Ratio (M‐H, Fixed, 95% CI)	0.34 [0.01, 8.40]
3.12 Dropouts due to treatment	4	1230	Risk Ratio (M‐H, Fixed, 95% CI)	0.80 [0.45, 1.45]

**Comparison 4:** Harms analyses: effects of bupropion only across comparisons

Outcome or subgroup title	No. of studies	No. of participants	Statistical method	Effect size
4.1 Adverse events	27	12313	Risk Ratio (M‐H, Fixed, 95% CI)	1.14 [1.11, 1.17]
4.1.1 Bupropion versus control	21	10931	Risk Ratio (M‐H, Fixed, 95% CI)	1.14 [1.11, 1.18]
4.1.2 Bupropion plus NRT versus NRT	3	339	Risk Ratio (M‐H, Fixed, 95% CI)	1.21 [1.03, 1.43]
4.1.3 Bupropion plus varenicline versus varenicline	4	1043	Risk Ratio (M‐H, Fixed, 95% CI)	1.09 [1.02, 1.17]
4.2 Psychiatric adverse events	10	5379	Risk Ratio (M‐H, Fixed, 95% CI)	1.23 [1.14, 1.32]
4.2.1 Bupropion versus control	8	4494	Risk Ratio (M‐H, Fixed, 95% CI)	1.25 [1.15, 1.36]
4.2.2 Bupropion plus NRT versus NRT	1	50	Risk Ratio (M‐H, Fixed, 95% CI)	0.50 [0.05, 5.17]
4.2.3 Bupropion plus varenicline versus varenicline	2	835	Risk Ratio (M‐H, Fixed, 95% CI)	1.15 [1.03, 1.30]
4.3 Serious adverse events	30	12883	Risk Ratio (M‐H, Fixed, 95% CI)	1.17 [0.93, 1.47]
4.3.1 Bupropion versus control	23	10958	Risk Ratio (M‐H, Fixed, 95% CI)	1.16 [0.90, 1.48]
4.3.2 Bupropion plus NRT versus NRT	4	657	Risk Ratio (M‐H, Fixed, 95% CI)	1.52 [0.26, 8.89]
4.3.3 Bupropion plus varenicline versus varenicline	5	1268	Risk Ratio (M‐H, Fixed, 95% CI)	1.23 [0.63, 2.42]
4.4 Seizures	15	8092	Risk Ratio (M‐H, Fixed, 95% CI)	2.93 [0.74, 11.54]
4.4.1 Bupropion versus control	13	7344	Risk Ratio (M‐H, Fixed, 95% CI)	2.93 [0.64, 13.37]
4.4.2 Buproion plus NRT versus NRT	1	527	Risk Ratio (M‐H, Fixed, 95% CI)	2.92 [0.12, 71.39]
4.4.3 Bupropion plus varenicline versus varenicline alone	1	0	Risk Ratio (M‐H, Fixed, 95% CI)	Not estimable
4.5 Overdoses	7	6135	Risk Ratio (M‐H, Fixed, 95% CI)	1.13 [0.21, 5.99]
4.5.1 Bupropion versus control	5	5585	Risk Ratio (M‐H, Fixed, 95% CI)	2.15 [0.23, 19.86]
4.5.3 Bupropion plus varenicline versus varenicline alone	2	550	Risk Ratio (M‐H, Fixed, 95% CI)	0.34 [0.01, 8.27]
4.6 Suicide attempts	13	8027	Risk Ratio (M‐H, Fixed, 95% CI)	0.88 [0.25, 3.14]
4.6.1 Bupropion versus control	10	6484	Risk Ratio (M‐H, Fixed, 95% CI)	1.62 [0.29, 8.92]
4.6.2 Buproion plus NRT versus NRT	1	0	Risk Ratio (M‐H, Fixed, 95% CI)	Not estimable
4.6.3 Bupropion plus varenicline versus varenicline alone	3	1056	Risk Ratio (M‐H, Fixed, 95% CI)	0.34 [0.04, 3.27]
4.7 Death by suicide	16	10036	Risk Ratio (M‐H, Fixed, 95% CI)	0.34 [0.01, 8.26]
4.7.1 Bupropion versus control	14	8822	Risk Ratio (M‐H, Fixed, 95% CI)	0.34 [0.01, 8.26]
4.7.2 Buproion plus NRT versus NRT	1	0	Risk Ratio (M‐H, Fixed, 95% CI)	Not estimable
4.7.3 Bupropion plus varenicline versus varenicline alone	2	0	Risk Ratio (M‐H, Fixed, 95% CI)	Not estimable
4.8 All‐cause mortality	24	12861	Risk Ratio (M‐H, Fixed, 95% CI)	0.81 [0.42, 1.58]
4.8.1 Bupropion versus control	21	11403	Risk Ratio (M‐H, Fixed, 95% CI)	0.89 [0.42, 1.87]
4.8.2 Buproion plus NRT versus NRT	2	731	Risk Ratio (M‐H, Fixed, 95% CI)	0.68 [0.12, 3.98]
4.8.3 Bupropion plus varenicline versus varenicline alone	2	727	Risk Ratio (M‐H, Fixed, 95% CI)	0.34 [0.01, 8.40]
4.9 Anxiety	15	9123	Risk Ratio (M‐H, Fixed, 95% CI)	1.45 [1.26, 1.67]
4.9.1 Bupropion versus control	11	7406	Risk Ratio (M‐H, Fixed, 95% CI)	1.42 [1.21, 1.67]
4.9.2 Buproion + NRT versus NRT	3	1218	Risk Ratio (M‐H, Fixed, 95% CI)	1.58 [0.97, 2.56]
4.9.3 Bupropion plus varenicline versus varenicline alone	2	499	Risk Ratio (M‐H, Fixed, 95% CI)	1.55 [1.01, 2.38]
4.10 Insomnia	25	12132	Risk Ratio (M‐H, Fixed, 95% CI)	1.73 [1.59, 1.88]
4.10.1 Bupropion versus control	22	11077	Risk Ratio (M‐H, Fixed, 95% CI)	1.78 [1.62, 1.96]
4.10.2 Buproion + NRT versus NRT	2	556	Risk Ratio (M‐H, Fixed, 95% CI)	1.55 [1.24, 1.93]
4.10.3 Bupropion plus varenicline versus varenicline alone	2	499	Risk Ratio (M‐H, Fixed, 95% CI)	1.45 [1.14, 1.84]
4.11 Dropouts due to treatment	31	14313	Risk Ratio (M‐H, Fixed, 95% CI)	1.42 [1.25, 1.60]
4.11.1 Bupropion versus control	25	12346	Risk Ratio (M‐H, Fixed, 95% CI)	1.44 [1.27, 1.65]
4.11.2 Bupropion + NRT versus NRT	3	737	Risk Ratio (M‐H, Fixed, 95% CI)	1.67 [0.95, 2.92]
4.11.3 Bupropion + varenicline versus varenicline	4	1230	Risk Ratio (M‐H, Fixed, 95% CI)	0.80 [0.45, 1.45]

**Comparison 5:** Bupropion versus varenicline

Outcome or subgroup title	No. of studies	No. of participants	Statistical method	Effect size
5.1 Smoking cessation	9	7564	Risk Ratio (M‐H, Fixed, 95% CI)	0.73 [0.67, 0.80]
5.2 Adverse events	6	5958	Risk Ratio (M‐H, Fixed, 95% CI)	0.98 [0.95, 1.00]
5.3 Serious adverse events	5	4920	Risk Ratio (M‐H, Fixed, 95% CI)	1.39 [0.94, 2.04]
5.4 Psychiatric adverse events	2	4051	Risk Ratio (M‐H, Fixed, 95% CI)	1.07 [0.99, 1.16]
5.5 Anxiety	2	4705	Risk Ratio (M‐H, Fixed, 95% CI)	1.28 [1.07, 1.53]
5.6 Insomnia	3	5208	Risk Ratio (M‐H, Fixed, 95% CI)	1.40 [1.22, 1.60]
5.7 Seizures	4	5389	Risk Ratio (M‐H, Fixed, 95% CI)	7.16 [0.92, 55.42]
5.8 Overdoses	2	4210	Risk Ratio (M‐H, Fixed, 95% CI)	0.92 [0.14, 6.25]
5.9 Suicide attempts	3	4239	Risk Ratio (M‐H, Fixed, 95% CI)	3.01 [0.31, 28.96]
5.10 Death by suicide	5	0	Risk Ratio (M‐H, Fixed, 95% CI)	Not estimable
5.11 All‐cause mortality	5	6074	Risk Ratio (M‐H, Fixed, 95% CI)	3.01 [0.31, 28.96]
5.12 Dropouts due to treatment	6	6111	Risk Ratio (M‐H, Fixed, 95% CI)	1.18 [1.00, 1.39]

**Comparison 6:** Bupropion versus nicotine replacement therapy (NRT)

Outcome or subgroup title	No. of studies	No. of participants	Statistical method	Effect size
6.1 Smoking cessation	14		Risk Ratio (M‐H, Fixed, 95% CI)	Subtotals only
6.1.1 Combination NRT (patch + lozenge)	2	720	Risk Ratio (M‐H, Fixed, 95% CI)	0.74 [0.55, 0.98]
6.1.2 Single form NRT	12	7613	Risk Ratio (M‐H, Fixed, 95% CI)	1.03 [0.93, 1.13]
6.1.3 Choice of NRT	2	361	Risk Ratio (M‐H, Fixed, 95% CI)	1.00 [0.79, 1.28]
6.2 Adverse events	4	4276	Risk Ratio (M‐H, Fixed, 95% CI)	1.02 [0.98, 1.06]
6.3 Psychiatric adverse events	3		Risk Ratio (M‐H, Fixed, 95% CI)	Totals not selected
6.4 Anxiety	2	4855	Risk Ratio (M‐H, Fixed, 95% CI)	1.31 [1.06, 1.62]
6.5 Insomnia	2	4128	Risk Ratio (M‐H, Fixed, 95% CI)	1.31 [1.10, 1.55]
6.6 Serious adverse events	8	6035	Risk Ratio (M‐H, Fixed, 95% CI)	1.22 [0.83, 1.80]
6.7 Seizures	1	4028	Risk Ratio (M‐H, Fixed, 95% CI)	0.34 [0.01, 8.24]
6.8 Overdoses	1	4028	Risk Ratio (M‐H, Fixed, 95% CI)	3.02 [0.12, 74.19]
6.9 Suicide attempts	2	4514	Risk Ratio (M‐H, Fixed, 95% CI)	1.68 [0.22, 12.75]
6.10 Death by suicide	2	0	Risk Ratio (M‐H, Fixed, 95% CI)	Not estimable
6.11 All‐cause mortality	3	5313	Risk Ratio (M‐H, Fixed, 95% CI)	1.36 [0.38, 4.84]
6.12 Dropouts due to treatment	4	4825	Risk Ratio (M‐H, Fixed, 95% CI)	1.14 [0.95, 1.38]

**Comparison 7:** Bupropion versus nortriptyline

Outcome or subgroup title	No. of studies	No. of participants	Statistical method	Effect size
7.1 Smoking cessation	3	417	Risk Ratio (M‐H, Fixed, 95% CI)	1.30 [0.93, 1.82]
7.2 Insomnia	2		Risk Ratio (M‐H, Fixed, 95% CI)	Totals not selected
7.3 Serious adverse events	1	0	Risk Ratio (M‐H, Fixed, 95% CI)	Not estimable
7.4 Dropouts due to treatment	2	240	Risk Ratio (M‐H, Fixed, 95% CI)	0.83 [0.47, 1.44]

**Comparison 8:** Bupropion versus gabapentin

Outcome or subgroup title	No. of studies	No. of participants	Statistical method	Effect size
8.1 Serious adverse events	1	0	Risk Ratio (M‐H, Fixed, 95% CI)	Not estimable
8.2 Dropouts due to treatment	1		Risk Ratio (M‐H, Fixed, 95% CI)	Totals not selected

**Comparison 9:** Bupropion (different doses)

Outcome or subgroup title	No. of studies	No. of participants	Statistical method	Effect size
9.1 Smoking cessation	3	2042	Risk Ratio (M‐H, Fixed, 95% CI)	1.08 [0.93, 1.26]
9.2 Anxiety	1		Risk Ratio (M‐H, Fixed, 95% CI)	Totals not selected
9.3 Insomnia	1		Risk Ratio (M‐H, Fixed, 95% CI)	Totals not selected
9.4 Serious adverse events	2	518	Risk Ratio (M‐H, Fixed, 95% CI)	1.33 [0.30, 5.94]
9.5 Overdoses	1		Risk Ratio (M‐H, Fixed, 95% CI)	Totals not selected
9.6 Suicide attempts	2	518	Risk Ratio (M‐H, Fixed, 95% CI)	0.34 [0.01, 8.17]
9.7 Death by suicide	2	0	Risk Ratio (M‐H, Fixed, 95% CI)	Not estimable
9.8 All‐cause mortality	2	518	Risk Ratio (M‐H, Fixed, 95% CI)	2.94 [0.12, 71.68]
9.9 Dropouts due to treatment	1		Risk Ratio (M‐H, Fixed, 95% CI)	Totals not selected

**Comparison 10:** Bupropion (different durations)

Outcome or subgroup title	No. of studies	No. of participants	Statistical method	Effect size
10.1 Smoking cessation	1	233	Risk Ratio (M‐H, Fixed, 95% CI)	1.45 [1.04, 2.03]

**Comparison 11:** Nortriptyline versus placebo

Outcome or subgroup title	No. of studies	No. of participants	Statistical method	Effect size
11.1 Smoking cessation	6	975	Risk Ratio (M‐H, Fixed, 95% CI)	2.03 [1.48, 2.78]
11.2 Anxiety	1		Risk Ratio (M‐H, Fixed, 95% CI)	Totals not selected
11.3 Insomnia	2	247	Risk Ratio (M‐H, Fixed, 95% CI)	0.58 [0.28, 1.21]
11.4 Serious adverse events	1		Risk Ratio (M‐H, Fixed, 95% CI)	Totals not selected
11.5 Dropouts due to treatment	4	537	Risk Ratio (M‐H, Fixed, 95% CI)	1.99 [1.18, 3.36]

**Comparison 12:** Nortriptyline plus nicotine replacement therapy (NRT) versus NRT alone

Outcome or subgroup title	No. of studies	No. of participants	Statistical method	Effect size
12.1 Smoking cessation	4	1644	Risk Ratio (M‐H, Fixed, 95% CI)	1.21 [0.94, 1.55]
12.2 Insomnia	1	158	Risk Ratio (M‐H, Fixed, 95% CI)	1.00 [0.30, 3.32]
12.3 Dropouts due to treatment	1		Risk Ratio (M‐H, Fixed, 95% CI)	Totals not selected

**Comparison 13:** Harms analyses: effects of nortriptyline only across comparisons

Outcome or subgroup title	No. of studies	No. of participants	Statistical method	Effect size
13.1 Serious adverse events	1		Risk Ratio (M‐H, Fixed, 95% CI)	Totals not selected
13.1.1 Nortriptyline versus placebo	1		Risk Ratio (M‐H, Fixed, 95% CI)	Totals not selected
13.2 Anxiety	1		Risk Ratio (M‐H, Fixed, 95% CI)	Totals not selected
13.2.1 Nortriptyline versus placebo	1		Risk Ratio (M‐H, Fixed, 95% CI)	Totals not selected
13.3 Insomnia	3	405	Risk Ratio (M‐H, Fixed, 95% CI)	0.67 [0.36, 1.25]
13.3.1 Nortriptyline versus placebo	2	247	Risk Ratio (M‐H, Fixed, 95% CI)	0.58 [0.28, 1.21]
13.3.2 Nortriptyline plus nicotine replacement therapy (NRT) versus NRT alone	1	158	Risk Ratio (M‐H, Fixed, 95% CI)	1.00 [0.30, 3.32]
13.4 Dropouts due to treatment	5	695	Risk Ratio (M‐H, Fixed, 95% CI)	2.40 [1.45, 3.96]
13.4.1 Nortriptyline versus placebo	4	537	Risk Ratio (M‐H, Fixed, 95% CI)	1.99 [1.18, 3.36]
13.4.2 Nortriptyline plus nicotine replacement therapy (NRT) versus NRT alone	1	158	Risk Ratio (M‐H, Fixed, 95% CI)	10.00 [1.31, 76.28]

**Comparison 14:** Selective serotonin reuptake inhibitors (SSRIs) versus placebo

Outcome or subgroup title	No. of studies	No. of participants	Statistical method	Effect size
14.1 Smoking cessation	4	1594	Risk Ratio (M‐H, Fixed, 95% CI)	0.93 [0.71, 1.22]
14.1.1 Fluoxetine	2	1236	Risk Ratio (M‐H, Fixed, 95% CI)	0.92 [0.65, 1.30]
14.1.2 Paroxetine	1	224	Risk Ratio (M‐H, Fixed, 95% CI)	1.08 [0.64, 1.82]
14.1.3 Sertraline	1	134	Risk Ratio (M‐H, Fixed, 95% CI)	0.71 [0.30, 1.64]
14.2 Adverse events	1		Risk Ratio (M‐H, Fixed, 95% CI)	Totals not selected
14.2.1 Fluoxetine	1		Risk Ratio (M‐H, Fixed, 95% CI)	Totals not selected
14.3 Dropouts due to treatment	3	1270	Risk Ratio (M‐H, Fixed, 95% CI)	2.59 [1.70, 3.94]
14.3.1 Fluoxetine	2	1136	Risk Ratio (M‐H, Fixed, 95% CI)	2.72 [1.75, 4.23]
14.3.2 Sertraline	1	134	Risk Ratio (M‐H, Fixed, 95% CI)	1.29 [0.30, 5.56]

**Comparison 15:** Selective serotonin reuptake inhibitor (SSRI) plus NRT versus NRT alone

Outcome or subgroup title	No. of studies	No. of participants	Statistical method	Effect size
15.1 Smoking cessation	3	466	Risk Ratio (M‐H, Fixed, 95% CI)	0.70 [0.48, 1.03]
15.1.1 Fluoxetine	3	466	Risk Ratio (M‐H, Fixed, 95% CI)	0.70 [0.48, 1.03]

**Comparison 16:** Monoamine oxidase inhibitor (MAOI) versus placebo

Outcome or subgroup title	No. of studies	No. of participants	Statistical method	Effect size
16.1 Smoking cessation	6	827	Risk Ratio (M‐H, Fixed, 95% CI)	1.29 [0.93, 1.79]
16.1.1 Moclobemide	1	88	Risk Ratio (M‐H, Fixed, 95% CI)	1.57 [0.67, 3.68]
16.1.2 Selegiline	5	739	Risk Ratio (M‐H, Fixed, 95% CI)	1.25 [0.88, 1.78]
16.2 Adverse events	2	391	Risk Ratio (M‐H, Fixed, 95% CI)	1.02 [0.93, 1.12]
16.2.1 Selegeline	1	101	Risk Ratio (M‐H, Fixed, 95% CI)	1.02 [0.91, 1.16]
16.2.2 EVT302	1	290	Risk Ratio (M‐H, Fixed, 95% CI)	1.02 [0.90, 1.15]
16.3 Psychiatric adverse events	1		Risk Ratio (M‐H, Fixed, 95% CI)	Subtotals only
16.3.1 Selegeline	1	5	Risk Ratio (M‐H, Fixed, 95% CI)	0.27 [0.02, 3.74]
16.4 Anxiety	2	427	Risk Ratio (M‐H, Fixed, 95% CI)	1.03 [0.48, 2.22]
16.4.1 Selegeline	1	101	Risk Ratio (M‐H, Fixed, 95% CI)	0.98 [0.42, 2.27]
16.4.2 Lazabemide	1	326	Risk Ratio (M‐H, Fixed, 95% CI)	1.25 [0.19, 8.32]
16.5 Insomnia	5	752	Risk Ratio (M‐H, Fixed, 95% CI)	1.50 [1.15, 1.97]
16.5.1 Moclobemide	1	87	Risk Ratio (M‐H, Fixed, 95% CI)	5.21 [1.64, 16.61]
16.5.2 Selegeline	3	339	Risk Ratio (M‐H, Fixed, 95% CI)	1.20 [0.91, 1.60]
16.5.3 Lazabemide	1	326	Risk Ratio (M‐H, Fixed, 95% CI)	2.66 [0.78, 9.00]
16.6 Serious adverse events	4	804	Risk Ratio (M‐H, Fixed, 95% CI)	1.17 [0.37, 3.68]
16.6.1 Moclobemide	1	0	Risk Ratio (M‐H, Fixed, 95% CI)	Not estimable
16.6.2 Selegeline	1	0	Risk Ratio (M‐H, Fixed, 95% CI)	Not estimable
16.6.3 Lazabemide	1	326	Risk Ratio (M‐H, Fixed, 95% CI)	0.53 [0.12, 2.32]
16.6.4 EVT302	1	290	Risk Ratio (M‐H, Fixed, 95% CI)	7.00 [0.36, 134.32]
16.7 Dropouts due to treatment	5	910	Risk Ratio (M‐H, Fixed, 95% CI)	1.75 [1.07, 2.86]
16.7.1 Moclobemide	1	87	Risk Ratio (M‐H, Fixed, 95% CI)	1.95 [0.38, 10.12]
16.7.2 Selegeline	2	203	Risk Ratio (M‐H, Fixed, 95% CI)	1.90 [0.94, 3.85]
16.7.3 Lazabemide	1	330	Risk Ratio (M‐H, Fixed, 95% CI)	1.58 [0.69, 3.62]
16.7.4 EVT302	1	290	Risk Ratio (M‐H, Fixed, 95% CI)	1.50 [0.25, 8.84]

**Comparison 17:** Venlafaxine versus placebo

Outcome or subgroup title	No. of studies	No. of participants	Statistical method	Effect size
17.1 Smoking cessation	1		Risk Ratio (M‐H, Fixed, 95% CI)	Subtotals only
17.2 Dropouts due to treatment	1		Risk Ratio (M‐H, Fixed, 95% CI)	Totals not selected

**Comparison 18:** Hypericum (St John's wort) versus placebo

Outcome or subgroup title	No. of studies	No. of participants	Statistical method	Effect size
18.1 Smoking cessation	2	261	Risk Ratio (M‐H, Fixed, 95% CI)	0.81 [0.26, 2.53]
18.2 Serious adverse events	1	143	Risk Ratio (M‐H, Fixed, 95% CI)	2.37 [0.36, 15.57]
18.3 All‐cause mortality	1	143	Risk Ratio (M‐H, Fixed, 95% CI)	3.08 [0.13, 73.24]
18.4 Dropouts due to treatment	1	141	Risk Ratio (M‐H, Fixed, 95% CI)	1.20 [0.36, 3.96]

**Comparison 19:** S‐adenosyl‐L‐methionine (SAMe) versus placebo

Outcome or subgroup title	No. of studies	No. of participants	Statistical method	Effect size
19.1 Smoking cessation	1		Risk Ratio (M‐H, Fixed, 95% CI)	Totals not selected
19.2 Adverse events	1	120	Risk Ratio (M‐H, Fixed, 95% CI)	1.58 [0.69, 3.65]
19.3 Insomnia	1	120	Risk Ratio (M‐H, Fixed, 95% CI)	1.54 [0.07, 36.11]
19.4 Dropouts due to treatment	1	120	Risk Ratio (M‐H, Fixed, 95% CI)	2.05 [0.24, 17.76]

**Comparison 20:** Selegiline plus nicotine replacement therapy (NRT) versus NRT alone

Outcome or subgroup title	No. of studies	No. of participants	Statistical method	Effect size
20.1 Serious adverse events	1	0	Risk Ratio (M‐H, Fixed, 95% CI)	Not estimable
20.2 Dropouts due to treatment	1	109	Risk Ratio (M‐H, Fixed, 95% CI)	1.42 [0.42, 4.75]

**Comparison 21:** EVT302 plus nicotine replacement therapy (NRT) versus NRT alone

Outcome or subgroup title	No. of studies	No. of participants	Statistical method	Effect size
21.1 Adverse events	1	122	Risk Ratio (M‐H, Fixed, 95% CI)	1.06 [0.90, 1.25]
21.2 Serious adverse events	1	122	Risk Ratio (M‐H, Fixed, 95% CI)	3.00 [0.12, 72.23]
21.3 Dropouts due to treatment	1	122	Risk Ratio (M‐H, Fixed, 95% CI)	3.00 [0.12, 72.23]

**Comparison 22:** Fluoxetine (30 mg versus 60 mg)

Outcome or subgroup title	No. of studies	No. of participants	Statistical method	Effect size
22.1 Smoking cessation	1		Risk Ratio (M‐H, Fixed, 95% CI)	Subtotals only
22.2 Dropouts due to treatment	1		Risk Ratio (M‐H, Fixed, 95% CI)	Totals not selected

**Comparison 23:** Lazabemide (100 mg versus 200 mg)

Outcome or subgroup title	No. of studies	No. of participants	Statistical method	Effect size
23.1 Serious adverse events	1		Risk Ratio (M‐H, Fixed, 95% CI)	Totals not selected
23.2 Anxiety	1		Risk Ratio (M‐H, Fixed, 95% CI)	Totals not selected
23.3 Insomnia	1		Risk Ratio (M‐H, Fixed, 95% CI)	Totals not selected
23.4 Dropouts due to treatment	1		Risk Ratio (M‐H, Fixed, 95% CI)	Totals not selected

**Comparison 24:** Hypericum (St John's wort) (300 mg versus 600 mg)

Outcome or subgroup title	No. of studies	No. of participants	Statistical method	Effect size
24.1 Smoking cessation	1		Risk Ratio (M‐H, Fixed, 95% CI)	Subtotals only
24.2 Adverse events	1		Risk Ratio (M‐H, Fixed, 95% CI)	Totals not selected

**Comparison 25:** S‐adenosyl‐L‐methionine (SAMe) (800 mg versus 1600 mg)

Outcome or subgroup title	No. of studies	No. of participants	Statistical method	Effect size
25.1 Adverse events	1		Risk Ratio (M‐H, Fixed, 95% CI)	Totals not selected
25.2 Dropouts due to treatment	1		Risk Ratio (M‐H, Fixed, 95% CI)	Totals not selected

## Characteristics of studies

### Characteristics of included studies [ordered by study ID]

**Table:** Abdelghany 2022

***Study characteristics***		
Methods	Study design: RCT Country: Egypt Setting: Chest medicine outpatient clinic Recruitment method: patients from the clinic	
Participants	100 smokers randomised; 3% female, average age 33; average cigarettes per day unknown, median FTND 6‐8	
Interventions	Placebo, 12 weeks Nicotine gum, 2 or 4 mg, 12 weeks Bupropion, 300 mg, 12 weeks Nicotine gum (2 or 4 mg) and Bupropion (300 mg), 12 weeks Common components: personalised counselling sessions were offered to all participants in the first visit lasting for about 10 to 15 minutes; subsequent weekly follow‐up visits were encouraged	
Outcomes	Smoking cessation: point prevalence at 26 weeks. Validated by CO < 6 ppm Adverse events: measured for 26 weeks	
Funding Source	Quote: "Nil."	
Author conflicts of interest	Quote: "There are no conflicts of interest."	
Notes		
***Risk of bias***		
**Bias**	**Authors' judgement**	**Support for judgement**
Random sequence generation (selection bias)	Low risk	Quote: "Simple randomization by using procedures such as coin‐tossing or dice‐rolling"
Allocation concealment (selection bias)	Low risk	Quote: "Sealed opaque envelopes... Participants were randomized (via sealed envelopes) and allocated to one of 4 groups"
Blinding (performance bias and detection bias) All outcomes	Unclear risk	The record claims that the study is placebo‐controlled, however only a bupropion placebo is described. The study is only single‐blind. Detection bias is minimised as cessation was biochemically verified.
Incomplete outcome data (attrition bias) All outcomes	High risk	Overall loss to follow‐up was less than 50%, but the difference between the nicotine gum and bupropion arms was more than 20%.
Other bias	Low risk	None detected

**Table:** Ahluwalia 2002

***Study characteristics***		
Methods	Study design: RCT Country: USA Setting: community‐based healthcare centre Recruitment method: community volunteers	
Participants	600 African American smokers randomised; 70% female, average age 44; average cigarettes per day 17; 27% had possible clinical depression CES‐D > 16	
Interventions	Bupropion, 300 mg/day for 7 weeks Placebo Common components: 8 sessions of in‐person or telephone counselling and self‐help guide	
Outcomes	Smoking cessation: prolonged abstinence at 26 weeks. Validated by CO ≤ 10 ppm, discrepancies resolved with cotinine ≤ 20 mg Adverse events: measured for 26 weeks	
Funding Source	National Cancer Institute. GlaxoSmithKline provided study medication.	
Author conflicts of interest	Dr Ahluwalia has served as a consultant for GlaxoSmithKline and Pharmacia Consumer. GlaxonSmithKline provided study medication but played no role in the design, conduct of the study, or interpretation and analysis of the data.	
Notes	Continuous abstinence rates shown in Figure 3 of paper. Figures obtained from authors	
***Risk of bias***		
**Bias**	**Authors' judgement**	**Support for judgement**
Random sequence generation (selection bias)	Low risk	Quote: "Randomization codes were generated in blocks of 50 and sent to the pharmaceutical company..."
Allocation concealment (selection bias)	Low risk	Quote: " ... [the pharmaceutical company]... packaged the treatment and then shipped the blinded drug to the investigator." Shows blinded drugs were provided to investigator
Blinding (performance bias and detection bias) All outcomes	Low risk	Quote: "Blinding was successful. At the end of treatment, 58% (150/259) of participants correctly guessed that they received bupropion SR [sustained release], and 41% (104/253) correctly guessed they received placebo."
Incomplete outcome data (attrition bias) All outcomes	Low risk	Approximately 32% lost to follow‐up in each group; included as smokers in meta‐analysis
Other bias	Low risk	None detected

**Table:** Anthenelli 2016

***Study characteristics***		
Methods	Study design: RCT Countries: USA, Australia, Canada, Denmark, Finland, Germany, New Zealand, South Africa, Spain, Bulgaria, Russian Federation, Slovakia, Argentina, Brazil, Chile, and Mexico Setting: clinical trial centres, academic centres, and outpatient clinics treating patients with and without psychiatric disorders Recruitment method: from the investigators' own clinics; through newspaper, radio, and television advertising; fliers and posters	
Participants	8144 participants; 56% female; average age 46.5; average cigarettes per day 21, mean FTND 5.8 Specialist population: participants were made up of two cohorts: a psychiatric cohort (N = 4074) and a non‐psychiatric cohort (N = 3984). Participants were included in the psychiatric cohort if they met Diagnostic and Statistical Manual of Mental Disorders, Fourth Edition, Text Revision (DSM‐IV‐TR) diagnostic criteria for mood disorders, including: major depressive disorder or bipolar disorder; anxiety disorders including panic disorder, with or without agoraphobia, post‐traumatic stress disorder, obsessive‐compulsive disorder, social phobia, and generalised anxiety disorder; psychotic disorders including schizophrenia and schizoaffective disorders; or borderline personality disorder. Participants in the non‐psychiatric cohort had no confirmed history of DSM‐IV‐TR Axis I or II disorders.	
Interventions	Bupropion sustained release and placebo varenicline and placebo nicotine patch. 150 mg twice a day for 12 weeks Varenicline and placebo bupropion sustained release and placebo nicotine patch. 1 mg twice a day for 12 weeks Transdermal nicotine patch and placebo varenicline and placebo bupropion sustained release. 21 mg per day with taper for 12 weeks Placebo bupropion sustained release and placebo varenicline and placebo nicotine patch. For 12 weeks. Common components: smoking cessation counselling consisting of 10 minute sessions at each of the 15 clinic visits, totalling 2 hours and 30 minutes	
Outcomes	Smoking cessation: continuous abstinence from week 9 to week 24 post‐quit date (validated by CO ≤ 10 ppm) Adverse events: measured within 12‐week treatment period, or for 30 days thereafter	
Funding Source	Pfizer and GlaxoSmithKline	
Author conflicts of interest	RMA reports receiving grants from Pfizer and Alkermes, and providing consulting and advisory board services to Pfizer, Arena Pharmaceuticals, and Cerecor. RMA's writing of this manuscript was supported, in part, by National Institute on Alcohol Abuse and Alcoholism grant numbers U01 AA013641 and R01 AA019720; National Institute on Drug Abuse/Veterans Affairs Co‐operative Studies numbers 1031 and 1032; and Veterans Affairs Merit Award number NEUA‐003‐08S. NLB reports providing consulting and advisory board services to Pfizer and GlaxoSmithKline, and having been a paid expert witness in litigation against tobacco companies. RW reports receiving grants from Pfizer, Johnson & Johnson, and GlaxoSmithKline, and receiving personal fees for advisory board services from Pfizer and GlaxoSmithKline. RW's salary is funded by Cancer Research UK. AEE reports receiving grants from Pfizer and Forum Pharmaceuticals, and receiving personal fees for advisory board services from Pfizer and Reckitt Benckiser. AEE's writing of the manuscript was supported by a National Institute on Drug Abuse Career Award in Patient‐Oriented Research, number K24 DA030443. LSA, TM, DL, and CR are employees and stockholders of Pfizer. JA is an employee of GlaxoSmithKline and stockholder of that company. AK is a PAREXEL employee working on behalf of GlaxoSmithKline.	
Notes		
***Risk of bias***		
**Bias**	**Authors' judgement**	**Support for judgement**
Random sequence generation (selection bias)	Low risk	Quote: "A randomisation administrator, independent from the clinical study team, prepared the computer‐generated randomisation schedule used to assign participants to treatment using a block size of 8 (1:1:1:1 ratio) for each of the 20 diagnosis by region combinations."
Allocation concealment (selection bias)	Low risk	Quote: "Investigators obtained participant identification numbers via a web‐based or telephone call‐in drug management system. Study product kit codes did not allow deciphering of randomised treatment or block size. As such, participants, investigators, and research personnel were masked to treatment assignments."
Blinding (performance bias and detection bias) All outcomes	Low risk	Quote: “The triple dummy design feature required participants to take study medications as masked tablets dispensed in separate varenicline and bupropion pill bottles each with matching placebo along with either applying active or placebo patches on a daily basis.” Quote: “Investigators obtained participant identification numbers via a web‐based or telephone call‐in drug management system. Study product kit codes did not allow deciphering of randomised treatment or block size. As such, participants, investigators, and research personnel were masked to treatment assignments.”
Incomplete outcome data (attrition bias) All outcomes	Low risk	439/2037 (21.6%) of the varenicline group, 448/2034 (22.0%) of the bupropion group, 481/2038 (23.6%) of the patch group, and 483/2035 (23.7%) of the placebo group were lost to follow‐up. Therefore, loss to follow‐up was less than 50% and similar across study arms.
Other bias	Low risk	None detected

**Table:** Aubin 2004

***Study characteristics***		
Methods	Study design: RCT Country: France Setting: 74 cessation outpatient clinics Recruitment: volunteers	
Participants	504 participants randomised: 56% female, average age 41, average cigarettes per day: not stated	
Interventions	Bupropion 300 mg for 7 weeks Placebo Common components: motivational support at clinic visits at baseline, weeks 3, 7, and 12, and 3 phone calls TQD, 2 to 3 days later, weeks 5 and 18	
Outcomes	Abstinence at week 26 (continuous from week 4) Validation: CO < 10 ppm	
Funding Source	GlaxoSmithKline	
Author conflicts of interest	The lead author (HJ Aubin) is a paid consultant of GSK.	
Notes	First included as Lebargy 2003 based on abstract	
***Risk of bias***		
**Bias**	**Authors' judgement**	**Support for judgement**
Random sequence generation (selection bias)	Low risk	Quote: "The computerized randomization schedule, prepared by the sponsor, was inaccessible to the investigator who was provided with a specific set of sequential treatment numbers."
Allocation concealment (selection bias)	Low risk	Quote: "The computerized randomization schedule, prepared by the sponsor, was inaccessible to the investigator who was provided with a specific set of sequential treatment numbers."
Blinding (performance bias and detection bias) All outcomes	Low risk	Quote: "Double‐blind" "Blinding was assured by matching the placebo to the bupropion tablets..."
Incomplete outcome data (attrition bias) All outcomes	Low risk	26% of the placebo and 27% of the bupropion groups lost; included as smokers in meta‐analysis
Other bias	Low risk	None detected

**Table:** Aveyard 2008

***Study characteristics***		
Methods	Study design: RCT Country: UK Setting: National Health Service stop smoking clinics Recruitment: people attending clinics	
Participants	901 smokers, ≥ 10/day; 46% female, average age 43, average cigarettes per day 21	
Interventions	Nortriptyline 75 mg/day, for 8 weeks including tapering (max dose for 6 weeks) Placebo capsules All participants received free NRT and had behavioural support, the majority attending group sessions run by cessation specialists	
Outcomes	Smoking cessation: prolonged abstinence at 12 months from day 15 post‐quit (validated by CO at 4 weeks, saliva cotinine (collected by post) at 6 months and 12 months)	
Funding Source	Cancer Research UK and National Institute for Health Research. Medication provided by King Pharmaceuticals	
Author conflicts of interest	PA has done consultancy work for the pharmaceutical and biotechnology industry that has led to payments to him and his institution. This includes work for companies providing smoking cessation treatment, including NRT. MM has received consultancy income from the European Network for Smoking Prevention and has provided scientific consultancy services through the University of Oxford ISIS Innovation to the National Audit Office and G‐Nostics.	
Notes		
***Risk of bias***		
**Bias**	**Authors' judgement**	**Support for judgement**
Random sequence generation (selection bias)	Low risk	Quote: "An independent statistician generated the randomisation schedule in Stata. We used block randomisation, with randomly ordered block sizes of two, four, and six, stratified by stop smoking adviser."
Allocation concealment (selection bias)	Low risk	Study nurses recruited participants, and the study administrator (who had not met the participants) allocated participants in sequence against the list for each adviser. Only the administrator and the pharmacist knew the allocation.
Blinding (performance bias and detection bias) All outcomes	Low risk	Quote: "Advisers, participants, and study staff... were blind to allocation... tablets were encapsulated, and identical powder filled capsules provided the placebos."
Incomplete outcome data (attrition bias) All outcomes	Low risk	12% intervention, 17% control lost at 12 months, included as smokers in meta‐analysis. Authors note that majority of losses were already smoking.
Other bias	Low risk	None detected

**Table:** Barnes 2006

***Study characteristics***		
Methods	Study design: RCT Country: UK Setting: private consulting room in a community pharmacy Recruitment method: advertisements were placed in newspapers regional to the pharmacy; information leaflets were placed in the pharmacy, along with a window display on smoking cessation which mentioned the study; local radio interviews were given	
Participants	28 participants randomised; 17% female; average age 42.8; average cigarettes per day 15.5; FTND: 26 participants < 8 and 2 participants ≥ 8	
Interventions	St John's wort (SJW), 300 mg per day St John's wort, 300 mg twice per day Common components: one hour of general smoking cessation advice and motivational support	
Outcomes	Smoking cessation: 12 months continuous abstinence following quit date (validated by CO)	
Funding Source	Lichtwer Pharma (UK) Ltd	
Author conflicts of interest	Lead author received funding by fellowship from Lichtwer Pharma UK Ltd	
Notes		
***Risk of bias***		
**Bias**	**Authors' judgement**	**Support for judgement**
Random sequence generation (selection bias)	Low risk	Quote: "A computer‐generated randomisation list of random treatment assignments ('A' or 'B', corresponding to lower and higher dosages of SJW, respectively) in blocks of 4 without stratification was prepared in advance."
Allocation concealment (selection bias)	High risk	Quote: “Participants enrolled into the study were assigned to the next consecutive treatment.” As the pharmacist was unblinded, they would therefore have been aware of the allocation of the participants.
Blinding (performance bias and detection bias) All outcomes	High risk	Quote: “This was a prospective, open, uncontrolled, pharmacy‐based, pilot study.”
Incomplete outcome data (attrition bias) All outcomes	High risk	11/15 in the once daily arm and 10/13 in the twice daily arm were lost to follow‐up. Therefore, loss to follow‐up is greater than 50% in each trial arm.
Other bias	Low risk	None detected

**Table:** Benli 2017

***Study characteristics***		
Methods	Study design: RCT Country: Turkey Setting: a smoking cessation clinic Recruitment method: participants applied to the smoking cessation clinic directly by calling the Turkish Ministry of Health's 'stop smoking' helpline and making an appointment.	
Participants	An unspecified number of participants were randomised. 405 participants were analysed. 17.5% female; average age 35.2; average cigarettes per day 23; mean FTND 6.3	
Interventions	Bupropion. Provided for 3 months Varenicline. Provided for 3 months Common components: behavioural therapy support with a biopsychosocial approach	
Outcomes	Smoking cessation: 7‐day ppa at 12 months. Validated by a CO level ≤ 5 ppm	
Funding Source	No funding	
Author conflicts of interest	The authors declare that they have no competing interests.	
Notes		
***Risk of bias***		
**Bias**	**Authors' judgement**	**Support for judgement**
Random sequence generation (selection bias)	Unclear risk	Quote: “Patients who were to receive the medication were randomly determined by the medication support center in order to provide a constant distribution rate of varenicline and bupropion and so that physicians would not be aware of the medication distribution.” Comment: no further detail is provided.
Allocation concealment (selection bias)	Unclear risk	Quote: “Patients who were to receive the medication were randomly determined by the medication support center in order to provide a constant distribution rate of varenicline and bupropion and so that physicians would not be aware of the medication distribution.” Comment: no further detail is provided.
Blinding (performance bias and detection bias) All outcomes	Unclear risk	Quote: “Patients who were to receive the medication were randomly determined by the medication support center in order to provide a constant distribution rate of varenicline and bupropion and so that physicians would not be aware of the medication distribution.” Comment: some attempt appears to have been made to blind physicians to group assignment; however, no further detail is given, so it is unclear whether participants and outcome assessors were blinded.
Incomplete outcome data (attrition bias) All outcomes	High risk	Only those followed up at 12 months are included in analysis
Other bias	Low risk	None detected

**Table:** Berlin 1995

***Study characteristics***		
Methods	Study design: RCT Country: France Setting: clinic Recruitment: by adverts in general practices or from occupational medicine departments	
Participants	88 smokers randomised; no current major depression or anxiety disorders; 57% had history of MDD	
Interventions	Moclobemide, 400 mg/day for 1 week pre‐ and 2 months post‐TQD, 200 mg for 3rd month Placebo No behavioural intervention or counselling	
Outcomes	Smoking cessation: prolonged abstinence at 1 year (validated at all visits up to 6 months by plasma cotinine ≤ 20 ng/mL. 1‐year abstinence based on telephone self‐report by 6 month quitters) Adverse events: measured until 91 days post‐quit	
Funding Source	Roche	
Author conflicts of interest	None specified	
Notes	There were no serious adverse reactions. Insomnia was more common in drug (36%) than placebo (7%) group. There were 4 dropouts for adverse effects/relapse in drug group and 2 in placebo group.	
***Risk of bias***		
**Bias**	**Authors' judgement**	**Support for judgement**
Random sequence generation (selection bias)	Unclear risk	Randomisation method not described
Allocation concealment (selection bias)	Unclear risk	Double‐blind, but blinding at allocation not explicit
Blinding (performance bias and detection bias) All outcomes	Unclear risk	Quote: "Double‐blind" but further detail not provided
Incomplete outcome data (attrition bias) All outcomes	Unclear risk	Quote: "Relapses and subjects lost from follow‐up were considered treatment failures." Number lost to follow‐up not reported
Other bias	Low risk	N/A

**Table:** Berlin 2002

***Study characteristics***		
Methods	Study design: RCT Countries: France and Belgium Setting: general practices and anti‐smoking clinics	
Participants	330 participants randomised; 43.9% female; average age 39.9; average cigarettes per day 24.7; mean FTND 6.2	
Interventions	Lazabemide, 50 mg twice daily for 8 weeks Lazabemide, 100 mg twice daily for 8 weeks Placebo, twice daily for 8 weeks Common components: brief cognitive behavioral intervention at each visit, totalling 2 hours	
Outcomes	Smoking cessation: follow‐up was 8 weeks, too short to be included in this review Adverse events: measured over a period of 8 weeks	
Funding Source	F Hoffmann‐La Roche	
Author conflicts of interest	None detailed	
Notes		
***Risk of bias***		
**Bias**	**Authors' judgement**	**Support for judgement**
Random sequence generation (selection bias)	Low risk	Quote: “subjects were assigned a treatment number according to the computer‐generated randomization table”
Allocation concealment (selection bias)	Unclear risk	Quote: “Eligible subjects were assigned a treatment number according to the computer‐generated randomization table.” Comment: no further information is provided, therefore who was blinded and how is unclear
Blinding (performance bias and detection bias) All outcomes	Unclear risk	“This was a randomized, placebo‐controlled, double‐blind, parallel‐group, multicenter proof‐of‐concept study” Comment: no further information is provided
Incomplete outcome data (attrition bias) All outcomes	High risk	60% in placebo (68/114), 62% in 100 mg/day lazabemide (67/108), and 54% in 200 mg/day lazabemide (58/108) were lost to follow‐up. Therefore, loss to follow‐up is above 50% in all groups.
Other bias	Low risk	None detected

**Table:** Berlin 2012

***Study characteristics***		
Methods	Study design: RCT Country: Germany Setting: investigation centres Recruitment method: media advertisements	
Participants	412 participants randomised; 37.4% female; average age 35; average cigarettes per day 19; mean FTND 5.4	
Interventions	EVT302, 1 x 5 mg tablet per day for 8 weeks (1 week pre‐quit and 7 weeks post‐quit) Placebo EVT302, 1 x 5 mg per day for 8 weeks (1 week pre‐quit and 7 weeks post‐quit) Placebo EVT302 and nicotine patch. Placebo EVT302 dosing was 1 x 5 mg per day for 8 weeks (1 week pre‐quit and 7 weeks post‐quit). Nicotine patch (21 mg/24 hours) was given for 7 weeks post‐quit. Common components: educational booklet on smoking cessation and a 10‐minute counselling session at each visit, totalling 1 hour and 50 minutes	
Outcomes	Smoking cessation: follow‐up was 12 weeks, too short to be included in this review Adverse events: recorded over 8 weeks	
Funding Source	Evotec NeuroSciences GmbH	
Author conflicts of interest	"Ivan Berlin has received consultancy payments and travel funding from Pfizer Ltd and Sanofi Aventis in the last 5 years. He received a consultancy payment from Evotec Ltd for preparing the current study's research protocol. Ian M Hunneyball, Doris Greiling, Stephen Jones and Hermann Fuder were employees of Evotec. Hans‐Detlev Stahl is an employee of ClinPharm International GmbH Prufzentrum Leipzig."	
Notes		
***Risk of bias***		
**Bias**	**Authors' judgement**	**Support for judgement**
Random sequence generation (selection bias)	Unclear risk	Quote: “Randomisation was performed by an independent statistician. A block size of 20 was used with each block containing medication assignments in a 7:7:3:3 ratio for EVT302 5 mg/day or placebo and EVT302 5 mg/day or placebo on top of NP [nicotine pill]. No stratification was used. Medication numbers were generated for a total of 25 blocks. The randomisation list was uploaded into the [interactive voice recognition system (IVRS)] allowing the centralised use of randomisation.” No detail of how sequences were generated
Allocation concealment (selection bias)	Low risk	Quote: "A central randomisation with an interactive voice recognition system (IVRS) was used which indicated the treatment to deliver upon the investigators’ call."
Blinding (performance bias and detection bias) All outcomes	Unclear risk	Study stated as being double‐blinded, although no further information is given beyond this. Nicotine pill is unblinded, however
Incomplete outcome data (attrition bias) All outcomes	Low risk	Dropout rates are all below 50% ‐ EVT302: 16 (10%); placebo: 14 (11%); EVT302 + nicotine pill: 5 (8%); placebo + nicotine pill: 2 (3%)
Other bias	Low risk	None detected

**Table:** Biberman 2003

***Study characteristics***		
Methods	Study design: RCT Country: Israel Setting: 3 community‐based clinics Recruitment: mailing to members of public health service provider	
Participants	109 smokers randomised; 38% females, average age 42, average cigarettes per day 27 to 30	
Interventions	Selegiline, 10 mg/day for 26 weeks, nicotine patch 21 mg for 8 weeks including tapering Placebo and nicotine patch Common components: behavioural support from trained family physician; weekly then fortnightly visits for 12 weeks	
Outcomes	Abstinence at 52 weeks, continuous with validation at each visit Validation: negative for urine nicotine, cotinine, 3‐hydroxycotinine (Niccheck)	
Funding Source	None specified	
Author conflicts of interest	None specified	
Notes	No serious AEs, no significant differences in AEs, 2 selegiline discontinuations	
***Risk of bias***		
**Bias**	**Authors' judgement**	**Support for judgement**
Random sequence generation (selection bias)	Low risk	Quote: "Four hundred dice‐throwing generated a randomized sequence code; 199 containers prepacked with selegiline and 201 containers prepacked with placebo were numbered accordingly." Comment: judged adequate
Allocation concealment (selection bias)	Low risk	Quote: "The code was sealed, kept secretly and was revealed for the first time when the last participant finished the 12 months of follow‐up. The first participant who joined the trial after the initial visit run‐in phase received the first bottle from the container set number 001, the second participant from set number 002 and so on. The trial coordinator arranged participant’s allocation." Comment: judged adequate
Blinding (performance bias and detection bias) All outcomes	Low risk	Quote: "Double‐blind" (see above) "No discontinuation difference for selegiline or placebo was observed among the groups, which implies masking success."
Incomplete outcome data (attrition bias) All outcomes	Low risk	19 lost to follow‐up, included as smokers in meta‐analysis
Other bias	Low risk	None detected

**Table:** Blondal 1999

***Study characteristics***		
Methods	Study design: RCT Country: Iceland Setting: cessation clinic Recruitment: community volunteers	
Participants	100 smokers randomised; 62% female; average age 41; average cigarettes per day 28	
Interventions	Nicotine inhaler and fluoxetine. Nicotine inhaler given for 3 months, with option of continuing for 3 months more. Fluoxetine dosing was 10 mg/day initiated 16 days before TQD, increased to 20 mg/day on day 6 Nicotine inhaler and placebo Common components: 5 x 1‐hour group behaviour therapy. Advised to use 6 to 12 inhalers/day for up to 6 months	
Outcomes	Smoking cessation: abstinence at 1 year (sustained from quit day). Validated by CO < 10 ppm at all assessments (6 weeks, 3 months, 6 months, 12 months) Adverse events: measured for 16 days	
Funding Source	Oddur Olafsson Fund, Pharmacia and Upjohn Consumer Health Care. Delta Pharmaceutical Company provided fluoxetine and placebo and fluoxetine analyses. Helsingborg, Sweden provided a grant, nicotine inhalers, and nicotine analyses.	
Author conflicts of interest	None specified	
Notes		
***Risk of bias***		
**Bias**	**Authors' judgement**	**Support for judgement**
Random sequence generation (selection bias)	Low risk	Computer‐generated randomisation; part of the randomisation procedure was performed by the manufacturer at another location where the code was also kept until it was broken in May 1997.
Allocation concealment (selection bias)	Low risk	Randomisation codes applied to pill boxes which were then allocated sequentially. "This part of the randomization procedure was performed by the manufacturer at another location where the code was also kept."
Blinding (performance bias and detection bias) All outcomes	Low risk	Quote: "Double‐blind." "...pill boxes, with either fluoxetine or an identical appearing placebo containing the same ingredients except fluoxetine, were labelled with numbers ranging from 100 to 210."
Incomplete outcome data (attrition bias) All outcomes	Unclear risk	Low numbers lost to follow‐up but reported results exclude 5 withdrawals: 3 from fluoxetine group due to adverse effects ‐ nervousness and anxiety; 1 from fluoxetine due to pregnancy; 1 from placebo who had purchased fluoxetine
Other bias	Low risk	None detected

**Table:** Brown 2007

***Study characteristics***		
Methods	Study design: 2 x 2 factorial RCT Country: USA Setting: 2 clinical sites (Butler Hospital, Miriam Hospital) Recruitment: community volunteers	
Participants	524 participants randomised; 48% female; average age 44; average cigarettes per day 25	
Interventions	Bupropion 300 mg/day for 12 weeks Placebo 2 x 2 factorial design. Alternative psychosocial treatments were standard cessation therapy or plus CBT for depression. Both had 12 x 90‐minute group sessions twice weekly, followed by weekly sessions and then monthly sessions over a total of 12 weeks. TQD 5th session. These arms were combined in analyses.	
Outcomes	Smoking cessation: abstinence at 12 months (sustained at 4 visits). Validated by CO ≤ 10 ppm, saliva cotinine ≤ 15 ng/mL Adverse events: measured for 12 weeks	
Funding Source	National Institutes of Health	
Author conflicts of interest	None specified	
Notes	First included as Brown 2006, part unpublished data. Some genotyping studies combine these participants with those reported in Collins 2004	
***Risk of bias***		
**Bias**	**Authors' judgement**	**Support for judgement**
Random sequence generation (selection bias)	Unclear risk	Quote: "Participants were randomly assigned to one of two treatment sites, where they were to receive one of two manualized group treatments ... Participants were then randomly assigned to receive one of two medication conditions, bupropion or placebo, using the urn randomization technique."
Allocation concealment (selection bias)	Unclear risk	Quote: "Whereas we were able to balance the drug and placebo conditions on an individual basis, behavioral treatments were randomized by group and thus were more susceptible to fluctuations in recruitment and to the availability at both sites of pairings of a senior and a junior therapist trained in CBTD". Knowledge of behavioural assignment was probably not concealed but seems unlikely to have led to individual selection bias.
Blinding (performance bias and detection bias) All outcomes	Low risk	Quote: "Double‐blind." Psychological condition unlikely to be blinded but unlikely to affect comparisons included in this review. "All participants and study staff were blind to medication condition."
Incomplete outcome data (attrition bias) All outcomes	Low risk	81% provided complete outcome data at all follow‐ups, not related to treatment condition. All participants included in ITT analyses
Other bias	Low risk	None detected

**Table:** Brown 2014

***Study characteristics***		
Methods	Study design: RCT Country: USA Setting: clinic Recruitment: via newspaper, radio, and television advertisements	
Participants	216 smokers with elevated depressive symptoms (CES‐D score ≥ 6) randomised; 38.4% female, average age 45.9; average cigarettes per day 21; mean FTND 5.6	
Interventions	Fluoxetine and nicotine patch, 10 weeks of 20 mg (beginning 2 weeks prior to TQD) Fluoxetine and nicotine patch, 16 weeks of 20 mg fluoxetine (beginning 8 weeks prior to TQD) Nicotine patch Common components: nicotine patch for 8 weeks starting on TQD (21 mg/day for 4 weeks, 14 mg/day for 2 weeks, 7 mg/day for last 2 weeks), 5 sessions of brief behavioural smoking cessation treatment (in person and by phone over 4 weeks, 20 to 30 minutes each), totalling 140 minutes	
Outcomes	Smoking cessation: continuous abstinence at 12 months. Validated by salivary cotinine < 10 ng/mL Adverse events: measured for 52 weeks	
Funding Source	American Cancer Society	
Author conflicts of interest	LHP reports receiving grant/research support from Medtronic, Neuronetics, HRSA, and NeoSync; serving on an advisory panel for Abbott; and serving as a consultant for Wiley, Springer, Qatar National Research Fund, and Abbott	
Notes	Significantly higher abstinence in 16‐week arm than in 10‐week arm; results presented separately in meta‐analysis with control divided. N abstinent not reported, extrapolated from percentages provided	
***Risk of bias***		
**Bias**	**Authors' judgement**	**Support for judgement**
Random sequence generation (selection bias)	Low risk	Quote: “randomly assigned to one of the three treatment conditions using urn randomization”
Allocation concealment (selection bias)	High risk	Quote: "Open‐label"
Blinding (performance bias and detection bias) All outcomes	High risk	Quote: "Open‐label"
Incomplete outcome data (attrition bias) All outcomes	Low risk	Over 90% followed up at 12 months. Similar rates across arms
Other bias	Low risk	None detected

**Table:** Cinciripini 2005

***Study characteristics***		
Methods	Study design: RCT Country: USA Setting: clinic Recruitment: community volunteers	
Participants	135 smokers randomised; 50% female, average age 46, average cigarettes per day 27	
Interventions	Venlafaxine, titrated to max. of 225 mg/day from 3 weeks before quit day for 21 weeks, including 2 weeks tapering Placebo Common components: 6 weeks, 22 mg nicotine patch, and 9 x 15‐minute behavioural counselling	
Outcomes	Smoking cessation: ppa at 12 months. Validated by CO ≤ 10 ppm and/or saliva cotinine < 15 ng/µL Adverse events: measured for unspecified period	
Funding Source	National Institutes for Health and National Institute for Drug Abuse. Medication provided free of charge by Wyeth Ayerst Laboratories.	
Author conflicts of interest	None specified	
Notes	First included as Cinciripini 1999 based on abstract	
***Risk of bias***		
**Bias**	**Authors' judgement**	**Support for judgement**
Random sequence generation (selection bias)	Unclear risk	Randomisation method not described. Stratification by depression history
Allocation concealment (selection bias)	Low risk	Randomisation by pharmacy, all study personnel with direct patient contact were blind
Blinding (performance bias and detection bias) All outcomes	Low risk	Quote: "Double‐blind... Blinding of the study staff to the medication was maintained using prenumbered pill containers, assigned to each participant at randomization by the pharmacy. All study personnel with direct patient contact were blind to group assignment."
Incomplete outcome data (attrition bias) All outcomes	Unclear risk	Majority of participants followed up (65 intervention; 63 control), participants lost to follow‐up counted as smokers in meta‐analysis
Other bias	Low risk	None detected

**Table:** Cinciripini 2013

***Study characteristics***		
Methods	Study design: RCT Country: USA Setting: clinic Recruitment: community volunteers	
Participants	294 participants randomised; 39% female; average age 44; average cigarettes per day 20; mean FTND 4.5	
Interventions	Bupropion, 12 weeks, started 12 to 19 days before TQD (150 mg/day on days 1 to 3, 300 mg/day thereafter) Varenicline, 12 weeks on same schedule (0.5 mg/day on days 1 to 3, 1.0 mg/day, days 4 to 7, 2.0 mg/day thereafter) Placebo, same schedule as above Common components: 10 individual counselling sessions (6 in person, 4 via phone, 240 minutes total)	
Outcomes	Smoking cessation: continuous abstinence after 2‐week grace period at 6 months (validated by CO < 10 ppm or salivary cotinine < 15 ng/mL) Adverse events: measured for 12 weeks	
Funding Source	National Institute on Drug Abuse, National Cancer Institute	
Author conflicts of interest	Dr Cinciripini served on the scientific advisory board of Pfizer, conducted educational talks sponsored by Pfizer on smoking cessation (2006‐2008), and has received grant support from Pfizer.	
Notes	In less than 1% of the total cases, participants who did not attend a follow‐up were coded as abstinent because they were abstinent at the following data point. All other losses to follow‐up counted as smokers. Author provided further detail on AE measurements via email.	
***Risk of bias***		
**Bias**	**Authors' judgement**	**Support for judgement**
Random sequence generation (selection bias)	Unclear risk	Quote: “Adaptive randomization”; no further detail provided
Allocation concealment (selection bias)	Unclear risk	Not reported
Blinding (performance bias and detection bias) All outcomes	Unclear risk	Quote: “Blinded” but no further information provided
Incomplete outcome data (attrition bias) All outcomes	Low risk	73% followed up at 6 months, similar rates across arms, all lost to follow‐up known to be smokers
Other bias	Low risk	None detected

**Table:** Cinciripini 2018

***Study characteristics***		
Methods	Study design: RCT Country: USA Setting: hospital‐based outpatient clinic specialising in cancer prevention Recruitment method: paid and unpaid media advertising	
Participants	385 participants randomised; 41.5% female; average age 49.0; average cigarettes per day 19.7; mean FTND 2.1	
Interventions	Bupropion and varenicline, 150 mg of bupropion per day for days 1 to 3, then 150 mg twice daily thereafter. 0.5 mg of varenicline per day for days 1 to 3, then 0.5 mg twice daily for days 4 to 7, then 1 mg twice daily thereafter Varenicline, dose and schedule given as in bupropion and varenicline intervention. Matching placebo for bupropion Matching placebo Common components: in‐person and phone counselling, totalling 215 minutes	
Outcomes	Smoking cessation: prolonged abstinence at 12 months, with relapse defined as smoking on 7 or more consecutive days or smoking at least one cigarette over 2 consecutive weeks within that same time interval (validated by CO < 4 ppm) Adverse events: measured for 12 months	
Funding Source	The project was supported by the United States National Institutes of Health (NIH) grant R01DA024709 (Principle Investigator PMC) and by the University of Texas MD Anderson’s Cancer Center Support Grant CA016672, funded by the National Cancer Institute (NCI). Pfizer (New York, NY) provided the active and matching placebo varenicline capsules.	
Author conflicts of interest	PMC served on the scientific advisory board of Pfizer Pharmaceuticals, conducted educational talks sponsored by Pfizer on smoking cessation (2006–08) and has received grant support and medication support from Pfizer. MKH participated in two multisite Pfizer‐funded trials and received varenicline from Pfizer to conduct four NIH‐funded trials.	
Notes		
***Risk of bias***		
**Bias**	**Authors' judgement**	**Support for judgement**
Random sequence generation (selection bias)	Low risk	Quote: “an algorithm developed and managed by study data managers, whose role was limited to data quality and integrity management”.
Allocation concealment (selection bias)	Unclear risk	No details as to how randomly‐generated sequence was transferred and implemented to staff delivering medication to participants.
Blinding (performance bias and detection bias) All outcomes	Low risk	Quote: “Participants, medical and research staff who interacted with participants and the study investigators were blinded to group assignment.”
Incomplete outcome data (attrition bias) All outcomes	Low risk	Dropout rates are as follows: 20/56 placebo; 48/166 varenicline; 38/163 combination. Dropout rates are below 50% in each arm.
Other bias	Low risk	None detected

**Table:** Collins 2004

***Study characteristics***		
Methods	Study design: RCT Country: USA Setting: 2 clinical research sites Recruitment: community volunteers	
Participants	555 participants randomised; excluded people with a history of psychiatric disorder, including MDD; 57% female, average age 46, average cigarettes per day 21	
Interventions	Bupropion. 300 mg/day for 10 weeks beginning 2 weeks before TQD Placebo Common components: 7 sessions group behavioural counselling	
Outcomes	Smoking cessation: prolonged abstinence at 6 months (from week 2, 7 consecutive days of smoking defined as relapse). Validated by saliva cotinine ≤ 15 ng/mL Adverse events: measured for unspecified period	
Funding Source	National Cancer Institute, National Institute on Drug Abuse, National Center for Research Resources. Treatment provided free of charge by GlaxoSmithKline.	
Author conflicts of interest	None specified	
Notes	Replaces Lerman 2002 which reported subset of data. Denominators supplied by 1st author, excludes 114 who withdrew before intervention. Some study details from Lerman 2006. Some genotyping studies combine these participants with those reported in Brown 2007.	
***Risk of bias***		
**Bias**	**Authors' judgement**	**Support for judgement**
Random sequence generation (selection bias)	Low risk	Quote: "Randomization was determined by a computer‐generated randomization scheme operated by a senior data manager; stratification was carried out by study site" (Lerman 2006)
Allocation concealment (selection bias)	Low risk	Centrally generated and allocation concealed from counsellors and assessors
Blinding (performance bias and detection bias) All outcomes	Unclear risk	Placebo used but blinding procedure not described in detail
Incomplete outcome data (attrition bias) All outcomes	Low risk	6% lost to follow‐up at 6‐month follow‐up; included as smokers in meta‐analysis
Other bias	Low risk	None detected

**Table:** Covey 2002

***Study characteristics***		
Methods	Study design: RCT Country: USA Setting: clinic Recruitment: volunteers	
Participants	134 smokers with a history of past MDD were randomised; 65% female; average age 44.5	
Interventions	Sertraline, starting dose 50 mg/day, 200 mg/day by week 4 quit day. 9‐day taper. Total duration 10 weeks + 9‐day taper, including 1‐week placebo washout prior to randomisation Placebo Common components: 9 x 45‐minute individual counselling sessions at clinic visits	
Outcomes	Smoking cessation: 7‐day ppa 6 months after end of treatment. Validated by serum cotinine < 25 ng/mL Adverse events: measured for 35 weeks	
Funding Source	Pfizer, Inc and National Institute on Drug Abuse	
Author conflicts of interest	"Pfizer, Inc., provided support for conducting the study.”	
Notes		
***Risk of bias***		
**Bias**	**Authors' judgement**	**Support for judgement**
Random sequence generation (selection bias)	Unclear risk	Randomisation method not described
Allocation concealment (selection bias)	Unclear risk	Not specified
Blinding (performance bias and detection bias) All outcomes	Low risk	Quote: "double‐blind" "Medications were provided in prepared bottles that were numbered according to the randomization schedule and dispensed at each visit. All study staff at the clinic site were blinded to treatment assignment."
Incomplete outcome data (attrition bias) All outcomes	Unclear risk	Quote: "The subjects lost to follow‐up after random assignment were considered treatment failures." Total participants lost to follow‐up at 6 months not reported
Other bias	Low risk	None detected

**Table:** Cox 2012

***Study characteristics***		
Methods	Study design: RCT Country: USA Setting: urban community‐based clinic Recruitment: volunteers, via healthcare settings and via community	
Participants	540 African American light smokers (≤ 10 cigarettes per day for ≥ 2 years, smoked on ≥ 25 days in past month); 66% female; average age 47; average cigarettes per day 8; average FTND 3.2	
Interventions	Bupropion, 300 mg for 7 weeks (150 mg once daily for 3 days, then 150 mg twice daily for remainder) Placebo, same schedule as bupropion Common components: up to 6 one‐to‐one 15‐ to 20‐minute individual counselling sessions, self‐help guide at start	
Outcomes	Smoking cessation: 7‐day ppa at 6 months. Validated by salivary cotinine < 15 ng/mL Adverse events: measured for 16 weeks	
Funding Source	National Cancer Institute, National Institutes of Health, National Institute for Minority Health and Disparities	
Author conflicts of interest	Dr JS Ahluwalia serves as a consultant to Pfizer Pharmaceuticals, Inc; Dr NL Benowitz serves as a consultant to Pfizer Pharmaceuticals, Inc, and has been a paid expert witness in litigation against tobacco companies; Dr RF Tyndale holds shares in Nicogen Research, Inc, a company that is focused on novel smoking cessation treatment approaches	
Notes	SAEs only reported at week 3 (none reported), not included in SAE analysis	
***Risk of bias***		
**Bias**	**Authors' judgement**	**Support for judgement**
Random sequence generation (selection bias)	Low risk	Computer‐generated random numbers table
Allocation concealment (selection bias)	Unclear risk	Not reported
Blinding (performance bias and detection bias) All outcomes	Unclear risk	Quote: "Both participants and investigators were blinded to the pharmacotherapy condition." No further information provided; unclear if counsellors blinded to treatment condition
Incomplete outcome data (attrition bias) All outcomes	Low risk	30% lost to follow‐up at 6 months; no difference between groups
Other bias	Low risk	None detected

**Table:** Da Costa 2002

***Study characteristics***		
Methods	Study design: RCT Country: Brazil Setting: cessation clinic Recruitment: volunteers to a smokers' support group	
Participants	144 smokers randomised; "predominantly female"; age and cigarettes per day not described	
Interventions	Nortriptyline, max. 75 mg/day for 6 weeks including titration period, begun 1 week before start of behaviour therapy Placebo Common components: 6‐weekly group CBT	
Outcomes	Smoking cessation: prolonged abstinence at 6 months after end of treatment (validation method not specified) Adverse events: measured for unspecified period	
Funding Source	None specified	
Author conflicts of interest	None specified	
Notes		
***Risk of bias***		
**Bias**	**Authors' judgement**	**Support for judgement**
Random sequence generation (selection bias)	Low risk	Quote: "Each patient chose a blind number from a box ...' Comment: probably adequate
Allocation concealment (selection bias)	Unclear risk	Quote: "... with each number corresponding to a “medication kit” that was externally undistinguishable. Patients and professionals participating in this study were blindfolded for this distribution." Comment: potentially adequate but difference in numbers in each group not accounted for
Blinding (performance bias and detection bias) All outcomes	Unclear risk	Quote: "Double‐blind" but insufficient detail provided
Incomplete outcome data (attrition bias) All outcomes	Unclear risk	Number lost in each group not clear
Other bias	Low risk	None detected

**Table:** Dalsgarð 2004

***Study characteristics***		
Methods	Study design: RCT Country: Denmark Setting: 5 hospitals Recruitment: hospital staff	
Participants	335 smokers, including physicians, nurses, other hospital service and administration staff; 75% female; average age 43; average cigarettes per day 19	
Interventions	Bupropion, 300 mg/day for 7 weeks Placebo Common components: motivational support around TQD, at weeks 3 and 7, and at 12‐week follow‐up	
Outcomes	Smoking cessation: prolonged abstinence at 6 months (starting from week 4). Validated by CO < 10 ppm	
Funding Source	GlaxoSmithKline	
Author conflicts of interest	None specified	
Notes		
***Risk of bias***		
**Bias**	**Authors' judgement**	**Support for judgement**
Random sequence generation (selection bias)	Low risk	Randomisation was computer‐generated and blinded
Allocation concealment (selection bias)	Low risk	Allocation was double‐blinded and bupropion and placebo tablets were identical in form and number.
Blinding (performance bias and detection bias) All outcomes	Unclear risk	Quote: "Double‐blind" Comment: clear that participants were blinded but unclear if all staff were blinded
Incomplete outcome data (attrition bias) All outcomes	Low risk	32% of the bupropion group and 43% of the placebo group discontinued treatment, included in analysis
Other bias	Low risk	None detected

**Table:** Ebbert 2014

***Study characteristics***		
Methods	Study design: RCT Country: USA Setting: Mayo Clinic in Rochester, Minnesota and University of Minnesota	
Participants	506 participants randomised; 47% female; average age 42.0; average cigarettes per day 19.6; mean FTND 5.3	
Interventions	Bupropion SR and varenicline. Bupropion SR was taken once daily (150 mg) for days 1 to 3, then twice daily (total of 300 mg/day) for 12 weeks. Varenicline was taken once daily (0.5 mg) for 3 days, then 0.5 mg twice daily (total of 1 mg/day) for days 4 to 7, and finally to the maintenance dose of 1 mg twice daily (total, 2 mg/day) for 11 weeks. Varenicline and placebo. Varenicline was taken according to the above dosing and schedule with matching placebo in place of bupropion. Common components: brief behavioral counselling at each clinic visit, totalling 110 minutes	
Outcomes	Smoking cessation: prolonged abstinence (no smoking from 2 weeks after the target quit date) at 52 weeks. Validated by CO Adverse events: measured for 52 weeks	
Funding Source	The clinical trial was supported by National Institutes of Health (NIH) grant CA 138417 (primary investigator, Dr Ebbert). Medication (varenicline) was provided by Pfizer	
Author conflicts of interest	"All authors have completed and submitted the ICMJE Form for Disclosure of Potential Conflicts of Interest. Dr Ebbert reports serving as an investigator for clinical trials funded by Pfizer, receipt of consultancy fees from GlaxoSmithKline, research support from Pfizer, and research support from Orexigen and JHP Pharmaceuticals outside of the current study. Dr Hatsukami reports receipt of research support from Nabi Biopharmaceuticals outside of the current study. Dr Hays reports serving as an investigator for clinical trials funded by Pfizer. Dr Hurt reports receipt of consulting fees from Pfizer, an unrestricted grant from Pfizer Medical Education Group, and provision of expert testimony in Florida tobacco litigation cases."	
Notes		
***Risk of bias***		
**Bias**	**Authors' judgement**	**Support for judgement**
Random sequence generation (selection bias)	Low risk	Quote: “computer‐generated randomization sequence with variable‐sized blocks ranging from 2 to 8 stratified by study site”.
Allocation concealment (selection bias)	Low risk	Central pharmacy was used to allocate interventions
Blinding (performance bias and detection bias) All outcomes	Low risk	Quote: “Study medication was labeled and dispensed according to participant identification, ensuring that treatment assignment remained concealed from the participant, investigators, and all study personnel having participant contact.”
Incomplete outcome data (attrition bias) All outcomes	Low risk	Dropout rates were as follows: 40/249 varenicline + bupropion; 42/257 varenicline + placebo Dropout rate was below 50% in all trial arms
Other bias	Low risk	None detected

**Table:** Eisenberg 2013

***Study characteristics***		
Methods	Study design: RCT Country: Canada Setting: 38 hospitals Recruitment: hospital patients with acute myocardial infarction	
Participants	392 smokers of at least 10 cigarettes per day, hospitalised with enzyme‐positive acute myocardial infarction. 16% female; average age 54; average cigarettes per day 23; average FTND not specified	
Interventions	Bupropion, 300 mg/day for 9 weeks (150 mg for 3 days, then 150 mg twice daily for remainder) Placebo, same schedule as bupropion Common components: 7 one‐to‐one counselling sessions by research nurses at baseline and all follow‐ups of < 20 minutes (average 5) – mix of phone and in‐person	
Outcomes	Smoking cessation: 12 months continuous abstinence (7 days ppa also reported). Validated by CO ≤ 10 ppm Adverse events: non‐SAEs measured for 9 weeks. SAEs measured for 12 months	
Funding Source	Canadian Institutes of Health Research and Heart and Stroke Foundation of Quebec	
Author conflicts of interest	Drs Eisenberg and Gervais reported that they served as paid consultants for Pfizer Canada Inc.'s Varenicline Advisory Board. Dr Gervais reported that he received funds from Pfizer Canada Inc. for lectures, including service on speaker bureaus, development of educational presentations, and travel/accommodations/meeting expenses. Dr Eisenberg received funding from Pfizer Canada Inc. to perform the Evaluation of Varenicline (Champix) in Smoking Cessation for Patients Post‐Acute Coronary Syndrome [EVITA] Trial; NCT00794573.	
Notes	Participants not allowed to smoke whilst hospitalised. SAEs reported over 12 months, so not included in analysis. Number of participants who quit extracted from percentages provided; denominators do not include 9 deaths in bupropion and 6 deaths in placebo group, all deemed not to be related to study medication. Adherence to treatment: 72.3% bupropion, 82% placebo took at least 1 pill per day	
***Risk of bias***		
**Bias**	**Authors' judgement**	**Support for judgement**
Random sequence generation (selection bias)	Low risk	Quote: "Randomization was done via an internet website using random blocks of 2 and 4 and was stratified by center to ensure that similar numbers of patients were randomized to the 2 arms of the study at each study center"
Allocation concealment (selection bias)	Low risk	Allocation performed centrally, see above
Blinding (performance bias and detection bias) All outcomes	Unclear risk	Quote: "Double‐blind." "All clinical end points were adjudicated by members of the Endpoints Evaluation Committee who were blinded to treatment assignment." Comment: no further information provided
Incomplete outcome data (attrition bias) All outcomes	Low risk	77% followed up at 12 months, similar across arms
Other bias	Low risk	None detected

**Table:** Elsasser 2002

***Study characteristics***		
Methods	Study design: RCT Country: USA Setting: community‐based Recruitment method: recruited from the community	
Participants	17 participants randomised; 41.2% female; average age 16.5; average cigarettes per day not specified; mean FTND not specified All participants were between 14 and 19 years old	
Interventions	Bupropion SR, 150 mg twice daily for an unspecified duration Matched placebo, same dose and duration as bupropion SR All participants received an unknown number and duration of behavioural modification sessions.	
Outcomes	Smoking cessation: prolonged abstinence between weeks 8 to 12 ‐ too short a follow‐up for consideration for this outcome as part of our review Adverse events: measured for 12 weeks	
Funding Source	Funding received from GlaxoWellcome	
Author conflicts of interest	None specified	
Notes	This study was reported in an abstract with 12‐week data, and so is not included in the analyses. However, the abstract states that this was a 52‐week study.	
***Risk of bias***		
**Bias**	**Authors' judgement**	**Support for judgement**
Random sequence generation (selection bias)	Unclear risk	Quote: “randomized, double‐blind, placebo‐controlled trial” Comment: no further information given
Allocation concealment (selection bias)	Unclear risk	Quote: “randomized, double‐blind, placebo‐controlled trial” Comment: no further information given
Blinding (performance bias and detection bias) All outcomes	Unclear risk	Quote: “randomized, double‐blind, placebo‐controlled trial” Comment: no further information given
Incomplete outcome data (attrition bias) All outcomes	High risk	Dropout rates were as follows: 2/9 (22.2%) in the placebo; 4/8 (50%) of the bupropion group. Therefore difference in dropout rate was higher than 20% between the two groups.
Other bias	Low risk	None detected

**Table:** Evins 2001

***Study characteristics***		
Methods	Study design: RCT Country: USA Setting: outpatient clinic Recruitment: volunteers	
Participants	18 smokers with stable schizophrenia (excluding 1 dropout prior to medication); 39% female; average age 45.5/42.7; average cigarettes per day 34	
Interventions	Bupropion. 300 mg/day for 3 months. TQD after week 3 Placebo Common components: 9 x 1‐hour weekly group CBT	
Outcomes	Smoking cessation: prolonged abstinence at 6 months. Validated by CO < 9 ppm or serum cotinine < 14 ng/mL Adverse events: measured for 24 weeks	
Funding Source	National Association for Research on Schizophrenia and Affective Disorders. Medication provided by GlaxoWellcome Inc	
Author conflicts of interest	None specified	
Notes	2‐year follow‐up also reported. 3 additional quitters, not used in meta‐analysis since additional therapy used	
***Risk of bias***		
**Bias**	**Authors' judgement**	**Support for judgement**
Random sequence generation (selection bias)	Unclear risk	Randomisation method not described
Allocation concealment (selection bias)	Unclear risk	Not specified
Blinding (performance bias and detection bias) All outcomes	Unclear risk	Quote: "Subjects were randomly assigned to 12 weeks of double‐blind bupropion SR, 150 mg/day, or an identical appearing placebo tablet added to their usual medication regimen." Comment: unclear if all staff members were blinded
Incomplete outcome data (attrition bias) All outcomes	Low risk	Quote: "Nineteen subjects were enrolled and 18 subjects completed the 6‐month smoking cessation trial."
Other bias	Low risk	None detected

**Table:** Evins 2005

***Study characteristics***		
Methods	Study design: RCT Country: USA Setting: clinic Recruitment: volunteers	
Participants	56 smokers with schizophrenia (excluding 6 dropouts prior to medication); 27% female; average age 45, average cigarettes per day 37/26	
Interventions	Bupropion, 300 mg/day for 3 months Placebo Common components: 12 sessions of group CBT	
Outcomes	Smoking cessation: 7 day ppa at 6 months. Validated by CO < 9 ppm Adverse events: measured for unspecified period	
Funding Source	National Association for Research on Schizophrenia and Affective Disorders. Medication provided by GlaxoSmithKline	
Author conflicts of interest	None specified	
Notes	There was a significant treatment effect at EOT.	
***Risk of bias***		
**Bias**	**Authors' judgement**	**Support for judgement**
Random sequence generation (selection bias)	Unclear risk	Randomisation method not stated
Allocation concealment (selection bias)	Unclear risk	Allocation concealment not described
Blinding (performance bias and detection bias) All outcomes	Unclear risk	Quote: "Double‐blind" with "identical placebo tablets." No further information provided
Incomplete outcome data (attrition bias) All outcomes	Low risk	Only people taking at least one dose of study medication included in analyses in paper. 5 in each group lost to follow‐up and included as smokers
Other bias	Low risk	None detected

**Table:** Evins 2007

***Study characteristics***		
Methods	Study design: RCT Country: USA Setting: community mental health centre Recruitment: outpatients	
Participants	51 smokers (≥ 10 cigarettes per day) with schizophrenia; average age 44; average cigarettes per day 28/25	
Interventions	Bupropion, 300 mg/day for 3 months, nicotine patch, 21 mg for 8 weeks including tapering, 2 mg nicotine gum Placebo and NRT, same schedule as bupropion 1 Common components: 12 sessions of group CBT, TQD week 4	
Outcomes	Smoking cessation: abstinence at 12 months from TQD. Validated by CO ≤ 8 ppm Adverse events: measured for unspecified period	
Funding Source	Massachusetts Department of Mental Health. Medication provided by GlaxoSmithKline	
Author conflicts of interest	None specified	
Notes	Used in bupropion plus NRT versus NRT comparison	
***Risk of bias***		
**Bias**	**Authors' judgement**	**Support for judgement**
Random sequence generation (selection bias)	Unclear risk	Randomisation method not described
Allocation concealment (selection bias)	Unclear risk	Allocation concealment not described
Blinding (performance bias and detection bias) All outcomes	Low risk	Quote: "Participants and investigators remained blind to the treatment condition (bupropion or placebo) throughout the follow‐up period." "Assessment of treatment assignment was at the level of chance for both participants and staff at Weeks 4 and 12 for both treatment assignments."
Incomplete outcome data (attrition bias) All outcomes	Low risk	20% of the bupropion group and 18% of the placebo group were lost to follow‐up at week 12; included as smokers in meta‐analysis. All other participants followed up at 12 months
Other bias	Low risk	None detected

**Table:** Evins 2008

***Study characteristics***		
Methods	Study design: RCT Country: USA Setting: no information available Recruitment method (quote): "Participants were recruited by advertisement and physician referral"	
Participants	49 smokers living with unipolar depressive disorder (major depression, minor depression, or dysthymic disorder) randomised; 49% female, average age 43; average cigarettes per day 25	
Interventions	Transdermal nicotine patches plus placebo pill. Patches: 21 mg for weeks 2 to 6, and 14 mg/day for weeks 7 to 8, and 7 mg/day for weeks 9 to 10 Transdermal nicotine patches plus bupropion SR. Patches: 21 mg for weeks 2 to 6, and 14 mg/day for weeks 7 to 8, and 7 mg/day for weeks 9 to 10. Bupropion: 150 mg for first 3 days then 150 mg twice daily Common components: Quote "All subjects received 13 sessions of group CBT, 8 weeks of NRT, and 12 weeks of either bupropion SR or identical placebo during the 13‐week acute treatment phase."	
Outcomes	Smoking cessation: investigators intended to measure cessation at 12‐month follow‐up; however, due to high loss to follow‐up, they do not report this. Therefore, cessation data are only reported to 13‐week follow‐up and are not included in this review, in‐line with our inclusion criteria. Adverse events: measured for 13 weeks, although these are not reported in the paper, other than in the form of dropouts due to adverse events.	
Funding Source	Quote: "This work was supported by NIDA 5R01DA011512 (Dr Fava) and NIDA 1K23DA00510‐01 (Dr Evins). GlaxoSmithKline provided sustained release bupropion and identical placebo."	
Author conflicts of interest	Quote: "Dr Evins has received research support from GlaxoSmithKline, Janssen Pharmaceutica, Pfizer, and the Bowman Family Foundation. Ms Culhane has no disclosures to declare. Dr Alpert has received research support from Abbott Laboratories, Alkermes, Lichtwer Pharma GmgH, Lorex Pharmaceuticals, Aspect Medical Systems, AstraZeneca, Bristol‐Myers Squibb Company, Cephalon, Eli Lilly & Company, Forest Pharmaceuticals Inc, GlaxoSmithKline, J & J Pharmaceuticals, Novartis, Organon Inc, PamLab, LLC, Pfizer Inc, Pharmavite, Roche, Sanofi/Synthelabo, Solvay Pharmaceuticals, Inc, and Wyeth‐Ayerst Laboratories. He has received speakers’ honoraria from Eli Lilly & Company, Janssen, and Organon. He has advisory/consultant relationships with Eli Lilly & Company, PamLab, LLC, and Pharmavite LLC. Dr Farabaugh has active grants with NIH‐NIMH and The JED Foundation. Dr Fava has received research support from Abbott Laboratories, Alkermes, Aspect Medical Systems, AstraZeneca, Bristol‐Myers Squibb Company, Cephalon, Eli Lilly & Company, Forest Pharmaceuticals Inc, GlaxoSmithkline, J & J Pharmaceuticals, Lichtwer Pharma GmbH, Lorex Pharmaceuticals, Novartis, Organon Inc, PamLab, LLC, Pfizer Inc, Pharmavite, Roche, Sanofi/Synthelabo, Solvay Pharmaceuticals Inc, and Wyeth‐Ayerst Laboratories. Dr Fava has advisory/consulting relationships with Amarin, Aspect Medical Systems, Astra‐Zeneca, Auspex Pharmaceuticals, Bayer AG, Best Practice Project Manage‐ ment Inc, Biovail Pharmaceuticals Inc, BrainCells Inc, Bristol‐Myers Squibb Company, Cephalon, CNS Response, Compellis, Cypress Pharmaceuticals, Dov Pharmaceuticals, Eli Lilly & Company, EPIX Pharmaceuticals, Fabre‐Kramer Pharmaceuticals Inc, Forest Pharmaceuticals Inc, GlaxoSmithkline, Grunenthal GmBH, Janssen Pharmaceutica, Jazz Pharmaceuticals, J & J Pharmaceuticals, Knoll Pharmaceutical Company, Lundbeck, MedAvante Inc, Merck, Neuronetics, Novartis, Nutrition 21, Organon Inc, PamLab, LLC, Pfizer Inc, PharmaStar, Pharmavite, Precision Human Biolaboratory, Roche, Sanofi/Synthelabo, Sepracor, Solvay Pharmaceuticals Inc, Somaxon, Somerset Pharmaceuticals, Takeda, and Wyeth‐Ayerst Laboratories. Dr Fava has received speakers’ honoraria from AstraZeneca, Boehringer‐Ingelheim, Bristol‐Myers Squibb Company, Cephalon, Eli Lilly & Company, Forest Pharmaceuticals Inc, GlaxoSmithkline, Novartis, Organon Inc, Pfizer Inc, PharmaStar, and Wyeth‐Ayerst Laboratories. Dr Fava has equity holdings with Compellis, MedAvante. Dr Fava has not received royalty/patent, and other income."	
Notes		
***Risk of bias***		
**Bias**	**Authors' judgement**	**Support for judgement**
Random sequence generation (selection bias)	Unclear risk	Quote: "The study used a block randomization based on the following components: level of nicotine dependence (high vs low as determined by the Fagerstrom Test of Nicotine Dependence (FTND) score of 7 or higher for high level of dependence), history of failed prior attempts to quit with an adequate trial of NRT and/or CBT by self‐report (yes or no), and either current or past UDD as determined by a research psychiatrist". No further information on how the sequence was generated.
Allocation concealment (selection bias)	Unclear risk	No information available
Blinding (performance bias and detection bias) All outcomes	Low risk	Placebo‐controlled trial; abstinence biochemically validated
Incomplete outcome data (attrition bias) All outcomes	High risk	Quote: "Because of the high rate of dropout in the 12‐month follow‐up period of this trial, we were unable to assess long‐term abstinence rates or the risk of relapse to MDD associated with tobacco abstinence in this sample."
Other bias	Low risk	None detected

**Table:** Fatemi 2013

***Study characteristics***		
Methods	Study design: RCT Country: USA Setting: not specified Recruitment method: not specified	
Participants	24 participants randomised; percentage female unspecified; average age not specified; average cigarettes per day not specified, mean FTND not specified All participants had been diagnosed with schizophrenia or schizoaffective disorder.	
Interventions	Varenicline, 1 mg twice daily for 12 weeks Buproprion SR, 150 mg twice daily for 12 weeks Matched placebo Common components: 20 minutes of antismoking counselling at each visit, totalling 80 minutes	
Outcomes	Smoking cessation: definition not specified Adverse events: measured for 12 weeks	
Funding Source	Grant support received from the National Institute on Drug Abuse (grant # R01DA024674‐01A1) to SHF. Pfizer provided free samples of varenicline and placebo and had no role in design or conduct of this study. Watson Laboratories provided free samples of Bupropion SR.	
Author conflicts of interest	None specified	
Notes		
***Risk of bias***		
**Bias**	**Authors' judgement**	**Support for judgement**
Random sequence generation (selection bias)	Unclear risk	No relevant information provided
Allocation concealment (selection bias)	Unclear risk	No relevant information provided
Blinding (performance bias and detection bias) All outcomes	Unclear risk	No relevant information provided
Incomplete outcome data (attrition bias) All outcomes	Unclear risk	Dropout rate was 41%, but difference between groups not detailed
Other bias	Low risk	None detected

**Table:** Ferry 1992

***Study characteristics***		
Methods	Study design: RCT Country: USA Setting: clinic Recruitment: not specified	
Participants	42 male smokers	
Interventions	Bupropion, 300 mg/day for 3 months Placebo Common components: group smoking cessation and relapse prevention counselling	
Outcomes	Smoking cessation: sustained abstinence at 6 months from end of treatment. Validated by saliva cotinine Adverse events: measured for unspecified period	
Funding Source	None specified	
Author conflicts of interest	None specified	
Notes	Abstract with no further details	
***Risk of bias***		
**Bias**	**Authors' judgement**	**Support for judgement**
Random sequence generation (selection bias)	Unclear risk	Randomisation method not described
Allocation concealment (selection bias)	Unclear risk	Allocation concealment not described
Blinding (performance bias and detection bias) All outcomes	Unclear risk	Quote: "Double‐blind"; no further detail provided
Incomplete outcome data (attrition bias) All outcomes	Unclear risk	No details given
Other bias	Low risk	None detected

**Table:** Ferry 1994

***Study characteristics***		
Methods	Study design: RCT Country: USA Setting: Veterans Medical Centre Recruitment: not specified	
Participants	190 smokers	
Interventions	Bupropion, 100 mg x 3/day for 12 weeks Placebo Common components: group smoking cessation and relapse prevention counselling; TQD within first 4 weeks	
Outcomes	Smoking cessation: prolonged abstinence at 12 months (from day 29). Validated by saliva cotinine ≤ 15 ng/mL at 6 months and 12 months Adverse events: measured for unspecified period	
Funding Source	None specified	
Author conflicts of interest	None specified	
Notes	Abstract with long‐term abstinence data supplied by author	
***Risk of bias***		
**Bias**	**Authors' judgement**	**Support for judgement**
Random sequence generation (selection bias)	Unclear risk	Randomisation method not described
Allocation concealment (selection bias)	Unclear risk	Allocation concealment not described
Blinding (performance bias and detection bias) All outcomes	Unclear risk	Quote: "Double‐blind," no further detail provided
Incomplete outcome data (attrition bias) All outcomes	Unclear risk	72% followed‐up intervention, 61% followed‐up control. "The most conservative approach to analysis would reclassify all of these individuals as smokers due to protocol violation."
Other bias	Low risk	None detected

**Table:** Fossati 2007

***Study characteristics***		
Methods	Study design: RCT Country: Italy Setting: primary care clinics Recruitment: patients of 71 general practitioners	
Participants	593 smokers randomised; 40% female; average age 49; average cigarettes per day 22	
Interventions	Bupropion, 300 mg/day for 7 weeks Placebo Common components: GP visits at enrolment and 4, 7, 26, and 52 weeks, phone calls 1 day pre‐TQD, 3 days post‐TQD, 10 weeks post‐enrolment. Classified as low intensity	
Outcomes	Smoking cessation: abstinence at 12 months (continuous from week 4). Validated by CO ≤ 10 ppm at each visit Adverse events: measured for 52 weeks	
Funding Source	Mario Negri Institute and GlaxoSmithKline	
Author conflicts of interest	Dr Apolone has received consulting and lecture fees from GlaxoSmithKline	
Notes		
***Risk of bias***		
**Bias**	**Authors' judgement**	**Support for judgement**
Random sequence generation (selection bias)	Unclear risk	Method of sequence generation not specified
Allocation concealment (selection bias)	Unclear risk	Stated to be double‐blind, but not explicit that GPs blinded to randomisation code
Blinding (performance bias and detection bias) All outcomes	Unclear risk	Quote: "Double‐blind"; further detail not provided
Incomplete outcome data (attrition bias) All outcomes	Low risk	15% bupropion and 17% placebo did not attend 12‐month follow‐up; included as smokers in meta‐analysis
Other bias	Low risk	None detected

**Table:** Gariti 2009

***Study characteristics***		
Methods	Study design: 2 x 2 factorial RCT Country: USA Setting: university Recruitment: self‐referral from community	
Participants	260 light smokers (6 to 15 cigarettes per day) motivated to quit; 57% female, average age 54; average cigarettes per day 11; average FTND 4	
Interventions	Bupropion SR and placebo patch. Bupropion for 9 weeks. Patch for 8 weeks. 10 weeks individualised counselling sessions Bupropion SR and placebo patch. Bupropion for 9 weeks. Patch for 8 weeks. Four 5‐ to 10‐minute counselling sessions Bupropion SR and nicotine patch. Bupropion for 9 weeks. Patch for 8 weeks. 10 weeks individualised counselling sessions Bupropion SR and nicotine patch. Bupropion for 9 weeks. Patch for 8 weeks. Four 5‐ to 10‐minute counselling sessions	
Outcomes	Smoking cessation: 7‐day ppa at 12 months. Validated by CO < 10 ppm; urinary cotinine < 200 ng/mL Adverse events: measured for unspecified period	
Funding Source	National Institute on Drug Abuse	
Author conflicts of interest	None specified	
Notes	Used in direct comparison of bupropion and NRT only, pooling 1 + 2 versus 3 + 4	
***Risk of bias***		
**Bias**	**Authors' judgement**	**Support for judgement**
Random sequence generation (selection bias)	Low risk	Computerised ‘urn randomization’
Allocation concealment (selection bias)	Unclear risk	Not specified
Blinding (performance bias and detection bias) All outcomes	Low risk	Quote: "double‐blind, double‐dummy" for medication component. "Neither the nurses nor the participants knew which of the two formulations contained the active formulation."
Incomplete outcome data (attrition bias) All outcomes	Low risk	Missing data included as smokers in meta‐analysis. Similar losses to follow‐up across both groups
Other bias	Low risk	None detected

**Table:** George 2002

***Study characteristics***		
Methods	Study design: RCT Country: USA Setting: mental health clinic Recruitment: outpatients	
Participants	32 smokers with schizophrenia motivated to quit; 44% female; average age 41/45; average cigarettes per day 24	
Interventions	Bupropion, 300 mg/day for 9 weeks. TQD 3 weeks Placebo Common components: 10 x 60‐minute weekly group therapy	
Outcomes	Smoking cessation: 7 day ppa at 6 months. Validated by expired CO < 10 ppm Adverse events: measured for unspecified period	
Funding Source	National institute on Drug Abuse, US Department of Veterans Affairs, National Alliance for Research on Schizophrenia and Depression. Medication provided by GlaxoSmithKline	
Author conflicts of interest	None specified	
Notes		
***Risk of bias***		
**Bias**	**Authors' judgement**	**Support for judgement**
Random sequence generation (selection bias)	Unclear risk	Randomisation method not described
Allocation concealment (selection bias)	Unclear risk	Not described
Blinding (performance bias and detection bias) All outcomes	Unclear risk	Quote: "Both subjects and research staff were blinded to study medication assignment. Study medications were prepared by research pharmacists at CMHC, using encapsulation of SR bupropion tablets with blue 00 opaque capsules; placebo capsules contained only a dextrose matrix."
Incomplete outcome data (attrition bias) All outcomes	Unclear risk	Quote: "Subjects who were lost during the trial or at 6‐month follow‐up were counted as smokers." Number followed‐up at 6 months not reported
Other bias	Low risk	None detected

**Table:** George 2003

***Study characteristics***		
Methods	Study design: RCT Country: USA Setting: outpatient smoking research clinic Recruitment: community volunteers	
Participants	40 smokers; 63% female; average age 49; average cigarettes per day 23	
Interventions	Selegiline. 10 mg/day for 9 weeks (5 mg/day in week 1 and week 9) Placebo	
Outcomes	Smoking cessation: 7 day ppa at 6 months. Validated by CO < 10 ppm Adverse events: measured for unspecified period	
Funding Source	None specified	
Author conflicts of interest	None specified	
Notes	"The main side effects of SEL were anorexia, gastrointestinal symptoms, and insomnia. None of the differences in adverse event ratings were significant in the SEL compared with the PLA group, and the drug was well tolerated compared with the placebo group. Reports of anxiety/agitation in both the SEL and PLA groups during the trial were high." Funding: National Institute on Drug Abuse, US Department of Veteran Affairs, National Alliance for Research on Schizophrenia and Depression	
***Risk of bias***		
**Bias**	**Authors' judgement**	**Support for judgement**
Random sequence generation (selection bias)	Unclear risk	Randomisation method not described
Allocation concealment (selection bias)	Unclear risk	Method not described
Blinding (performance bias and detection bias) All outcomes	Low risk	Double‐blind, adequacy of blinding tested in research staff; results suggested blinding was adequate
Incomplete outcome data (attrition bias) All outcomes	High risk	29/40 not assessed at 6 months. Greater loss to follow‐up in placebo, exact data not reported
Other bias	Low risk	None detected

**Table:** George 2008

***Study characteristics***		
Methods	Study design: RCT Country: USA Setting: mental health centre Recruitment: outpatients	
Participants	58 smokers with schizophrenia or schizoaffective disorder (excludes 1 receiving no study medication); 40% female; average age 40; average cigarettes per day ~23	
Interventions	Bupropion, 300 mg/day for 9 weeks, begun 7 days pre‐TQD Placebo Common components: nicotine patch (21 mg/24 hours) for 8 weeks from TQD and group behaviour therapy 10‐weekly sessions	
Outcomes	Smoking cessation: ppa at 6 months. Validated by CO < 10 ppm Adverse events: measured for unspecified period	
Funding Source	National Institute on Drug Abuse, National Alliance for Research on Schizophrenia and Depression	
Author conflicts of interest	None specified	
Notes	Bupropion as adjunct to NRT	
***Risk of bias***		
**Bias**	**Authors' judgement**	**Support for judgement**
Random sequence generation (selection bias)	Unclear risk	Randomisation method not described
Allocation concealment (selection bias)	Unclear risk	Not described
Blinding (performance bias and detection bias) All outcomes	Unclear risk	Quote: "Double blind" but no additional details given
Incomplete outcome data (attrition bias) All outcomes	Low risk	6/29 intervention and 10/29 control did not complete trial; included as smokers in meta‐analysis
Other bias	Low risk	None detected

**Table:** Gilbert 2019

***Study characteristics***		
Methods	Study design: RCT Country: USA Setting: from the community Recruitment method: newspaper ads and community and university postings	
Participants	105 participants randomised; 42% female; average age 26.4; average cigarettes per day 17.9, mean FTND 4.2	
Interventions	Bupropion SR and placebo nicotine patch. 150 mg pill once daily for 3 days, then twice daily for 56 days, then once daily for three days. Placebo nicotine patch schedule given below Nicotine patch and placebo bupropion. Beginning on first day of cessation: 21 mg for 24 days, 14 mg for 14 days, then 7 mg for 7 days. Placebo bupropion schedule as given above Matched placebos, according to the schedules given above Common components: an abbreviated form of the American Lung Association smoking cessation program	
Outcomes	Smoking cessation: prolonged abstinence at 12 months. Validation method not specified Adverse events: measured for 62 days	
Funding Source	Supported by the National Institute on Drug Abuse (NIDA) Grant R01 DA012289 awarded to David G Gilbert	
Author conflicts of interest	None specified	
Notes		
***Risk of bias***		
**Bias**	**Authors' judgement**	**Support for judgement**
Random sequence generation (selection bias)	Low risk	Quote: randomised by “urn technique without replacement approach via a 28:28:28:16 ratio to one of four groups.”
Allocation concealment (selection bias)	Unclear risk	No relevant information given
Blinding (performance bias and detection bias) All outcomes	Low risk	Quote: “Researchers and participants in the quit groups were blind to pill and patch type.”
Incomplete outcome data (attrition bias) All outcomes	Low risk	Dropout rates: 0/34 – bupropion; 0/38 – nicotine patch; 0/35 – placebo
Other bias	Low risk	None detected

**Table:** Gonzales 2001

***Study characteristics***		
Methods	Study design: RCT Country: USA Setting: 16 clinical trial centres Recruitment: volunteers who had previously failed to quit using bupropion	
Participants	450 smokers who had previously used bupropion for at least 2 weeks without adverse effects and failed to quit; 55% female in placebo arm, 48% female in bupropion arm; average age 45; average cigarettes per day not specified	
Interventions	Bupropion, 300 mg/day for 12 weeks, begun 7 days pre‐TQD Placebo Common components: brief individual counselling at visits weeks 1 through 7, 9, 12, plus telephone counselling at 4 months and 5 months	
Outcomes	Smoking cessation: prolonged abstinence 12 months, starting from week 4. Validated by CO ≤ 10 ppm at each visit Adverse events: measured for unspecified duration	
Funding Source	GlaxoWellcome Inc	
Author conflicts of interest	None specified	
Notes	6‐month data published. 12‐month data presented in a poster used since 2003 update	
***Risk of bias***		
**Bias**	**Authors' judgement**	**Support for judgement**
Random sequence generation (selection bias)	Low risk	Quote: "Participants who satisfied the inclusion criteria were randomized to the treatment phase and received either bupropion SR ... or matching placebo. Eligible participants were assigned a protocol‐specific treatment number on the basis of a randomization code provided by GlaxoWellcome."
Allocation concealment (selection bias)	Unclear risk	Allocation concealment method not described
Blinding (performance bias and detection bias) All outcomes	Low risk	Quote: "Even though participants and the site staff were blinded to the drug assignments and the site staff did not encourage participants to speculate on their assignments, the lower placebo abstinence rates in the current study may be attributable to the previous experiences of participants with bupropion in their previous cessation attempts." However, little difference in completion between two arms, suggesting blinding may have been successful.
Incomplete outcome data (attrition bias) All outcomes	Unclear risk	Quote: "...all participants who stopped participating in the study during the treatment phase were considered to be smokers." Number of participants followed‐up at 12 months unclear
Other bias	Low risk	None detected

**Table:** Gonzales 2006

***Study characteristics***		
Methods	Study design: RCT Country: USA Setting: 19 clinical trial centres Recruitment: community volunteers	
Participants	673 participants, with prior exposure to bupropion excluded; 46% female; average age 42; average cigarettes per day 21	
Interventions	Bupropion, 300 mg/day for 12 weeks, begun 7 days pre‐TQD Varenicline, 2 mg/day Placebo Common components: brief (< 10‐minute) standardised individual counselling at 12 weekly visits during drug phase and 11 clinic/phone visits during follow‐up, problem solving and relapse prevention	
Outcomes	Smoking cessation: sustained abstinence at 1 year (starting from week 4). Validated by CO ≤ 10 ppm at each visit Adverse events: measured for 13 weeks	
Funding Source	Pfizer, Inc	
Author conflicts of interest	"Dr Gonzales reports having received research contracts from Pfizer, Sanofi‐Aventis, GlaxoSmithKline, and Nabi Biopharmaceuticals; consulting fees and honoraria from Pfizer, Sanofi‐Aventis, and GlaxoSmithKline; and owning 5 shares of Pfizer stock. Dr Rennard reports having had or currently having a number of relationships with companies who provide product and/or services relevant to outpatient management of COPD. These relationships include serving as a consultant for Adams, Almirall, Altana, Array Biopharma, AstraZeneca, Aventis, Biolipox, Centocor, Dey, Critical Therapeutics, GlaxoSmithKline, Johnson & Johnson, Merck, Novartis, Ono Pharma, Otsuka, RJ Reynolds, Roche, Sankyo, Schering‐Plough, Scios, and Wyeth; advising regarding clinical trials for Altana, Astra‐Zeneca, Aventis, Centocor, GlaxoSmithKline, Novartis, Pfizer, and Philip Morris; and speaking at continuing medical education programs and performing funded research both at basic and clinical levels for Altana, Astra‐Zeneca, Boehringer Ingelheim, GlaxoSmithKline, and Novartis. Dr Nides reports having received research grants, consulting fees, and honoraria from Pfizer, Sanofi‐Aventis, and GlaxoSmithKline. Dr Oncken reports having received research grants, consulting fees, and honoraria from Pfizer; receiving, at no cost, nicotine replacement and placebo products from GlaxoSmithKline for smoking cessation studies; and receiving honoraria from Pri‐Med. Drs Azoulay, Watsky, Gong, Williams, and Reeves and Mr Billing report owning Pfizer stock or having stock options in Pfizer.	
Notes	Bupropion was an active control for varenicline. Bupropion versus placebo and bupropion versus varenicline comparisons contribute to review	
***Risk of bias***		
**Bias**	**Authors' judgement**	**Support for judgement**
Random sequence generation (selection bias)	Low risk	Quote: "predefined ... computer‐generated randomization sequence", 1:1:1, using block size of 6, stratified by centre
Allocation concealment (selection bias)	Low risk	Central allocation
Blinding (performance bias and detection bias) All outcomes	Low risk	Quote: "Participants and investigators were blinded to drug treatment assignments[, and] ... were not encouraged to guess their treatment assignment".
Incomplete outcome data (attrition bias) All outcomes	Low risk	Loss to follow‐up similar across conditions: 44% bupropion, 39.5% varenicline, 46% placebo, all included in analyses
Other bias	Low risk	None detected

**Table:** Grant 2007

***Study characteristics***		
Methods	Study design: RCT Country: USA Setting: 2 substance use disorder clinics Recruitment: alcoholics in residential or outpatient treatment programmes	
Participants	58 alcoholic smokers; 16% female; average age 40; average cigarettes per day 25	
Interventions	Bupropion, 300 mg for 60 days plus nicotine patch 21 mg for 8 weeks including tapering Placebo and nicotine patch Common components: 1‐hour cessation group (and 4‐weekly assessment visits)	
Outcomes	Smoking cessation: 7 day ppa at 6 months. No biochemical validation, collaterals contacted, inconsistent, adjusted rates not reported Adverse events: measured for 4 weeks	
Funding Source	National Institute on Alcohol Abuse and Alocholism	
Author conflicts of interest	None specified	
Notes		
***Risk of bias***		
**Bias**	**Authors' judgement**	**Support for judgement**
Random sequence generation (selection bias)	Unclear risk	Randomisation method not described
Allocation concealment (selection bias)	Unclear risk	Not described
Blinding (performance bias and detection bias) All outcomes	Unclear risk	Quote: "Double‐blind" but unclear who was blinded; no further information provided
Incomplete outcome data (attrition bias) All outcomes	Low risk	Higher loss in bupropion (40%) than placebo (21%) but still within 20% range of each other. ITT analysis
Other bias	Low risk	None detected

**Table:** Gray 2011

***Study characteristics***		
Methods	Study design: RCT Country: USA Setting: university research clinic or high school health clinic Recruitment method: through local secondary schools, colleges, universities, and community media advertisements	
Participants	All participants were between 12 and 21 years 134 participants randomised; 41.8% female; average age 18.5; average cigarettes per day 10.8; mean FTND 4.2	
Interventions	Bupropion and contingency management. 150 mg once daily for three days, then 150 mg twice daily for remainder of 6‐week treatment period. Contingency management consisted of monetary compensation for biologically verified abstinence at visits. Abstinence at the first visit was 10 USD (dollars), with subsequent consecutive abstinent visits escalating by USD 3 (USD 13, USD 16, USD 19, and so on). If a participant relapsed, he or she was not eligible for contingent compensation at that visit, and the contingent reward for the next abstinent visit was reset to USD 10 (with eligibility to escalate by USD 3 at subsequent abstinent visits). Thus, the maximum amount of compensation throughout the 6‐week treatment period was USD 275. Bupropion and non‐contingency management. Bupropion given according to schedule above. Non‐contingency management consisted of fixed compensation (USD 10 per visit) for attending the twice‐weekly treatment visits. Matched placebo and contingency management Matched placebo and non‐contingency management All participants received smoking cessation booklets and were eligible for a weekly bonus payment of USD 5 throughout active treatment for completion of study materials, including daily smoking diaries. In addition, all participants received USD 30 for completing the initial assessment visit, USD 20 for completing the initial medication management visit, and USD 20 for completing the final post‐treatment follow‐up visit.	
Outcomes	Smoking cessation: 12 weeks ‐ too short a follow‐up for this outcome to be considered in this review Adverse events: measured for 6 weeks	
Funding Source	Funding was provided by the National Institute on DrugAbuse, Grants R01 DA17460 (HPU, KMG), K12DA000357 (KMG), K23 DA020482 (MJC), and R25DA020537 (ALL); by the Eunice Kennedy Shriver National Institute of Child Health and Human Development, Grant K12HD055885 (KJH); and by the US Public Health Service, Grant M01 RR01070 (Medical University of South Carolina Clinical and Translational Research Center)	
Author conflicts of interest	"Dr Gray has received research support from Pfizer, Inc. (medication and placebo supply for research funded by the National Institute on Drug Abuse). Dr Hartwell has received grant support through Global Research Awards for Nicotine Dependence, an independent competitive grants program supported by Pfizer Inc. Dr Hiott is a past speakers' bureau member of Bristol‐Myers Squibb and Abbott Labs. Dr Deas has been an advisory board and speakers' bureau member of Eli Lilly and Company. Dr Upadhyaya is a past consultant and/or advisory board member of Eli Lilly and Company and Shire Pharmaceuticals. Dr Upadhyaya is an ex‐stockholder of New River Pharmaceutical Company, is a past speakers' bureau member of Shire Pharmaceuticals and Pfizer, Inc., and has received research support from Cephalon, Inc., Eli Lilly and Company, and Pfizer Inc. Dr Upadhyaya recently became an employee of, and is a holder of stock in, EliLilly and Company. The other investigators deny any potential conflicts of interest."	
Notes		
***Risk of bias***		
**Bias**	**Authors' judgement**	**Support for judgement**
Random sequence generation (selection bias)	Unclear risk	No relevant information given
Allocation concealment (selection bias)	Unclear risk	No relevant information given
Blinding (performance bias and detection bias) All outcomes	Unclear risk	Quote: “double blinded; encapsulated by the university Investigational Drug Service so that the active and placebo medication appeared identical”. No further information given
Incomplete outcome data (attrition bias) All outcomes	Low risk	Dropout rates: 12/37 (32.4%) in bupropion and contingency management; 13/36 (36.1%) in bupropion and non‐contingency management; 14/29 (48.3%) in placebo and contingency management; 10/32 (31.3%) in placebo and non‐contingency management Loss to follow‐up was less than 50% and similar across groups.
Other bias	Low risk	None detected

**Table:** Gray 2012

***Study characteristics***		
Methods	Study design: RCT Country: USA Setting: community Recruitment method: community media advertisement (e.g. flyers, newspapers, advertisements)	
Participants	29 participants randomised; 51.8% female; average age 18.9; average cigarettes per day 15.6; mean FTND 6.7 Adolescent smokers, aged 15 to 20 years	
Interventions	Bupropion XL plus placebo. 150 mg once daily for 7 days, then 300 mg daily thereafter. Placebo capsules were used at times when no active medication was scheduled. Varenicline plus placebo. Participants ≥ 55 kg received 0.5 mg daily for 3 days, 0.5 mg twice daily for 4 days, and then 1 mg twice daily thereafter. Those < 55 kg received 0.5 mg daily for 7 days and then 0.5 mg twice daily thereafter All participants received quit smoking brochures and brief individual cessation counselling, totalling 90 minutes.	
Outcomes	Smoking cessation: 12 weeks ‐ too short a follow‐up for this outcome to be considered in this review Adverse events: measured for 12 weeks	
Funding Source	Medical University of South Carolina Hollings Cancer Center Pilot Research Program and the National Institutes of Health (K12DA000357, K23DA020482, R25DA020537, and UL1RR029882)	
Author conflicts of interest	"Dr Upadhyaya is an employee and stockholder of Eli Lilly and Company. The other authors do not have potential conflicts to declare."	
Notes		
***Risk of bias***		
**Bias**	**Authors' judgement**	**Support for judgement**
Random sequence generation (selection bias)	Unclear risk	No relevant information given
Allocation concealment (selection bias)	Unclear risk	No relevant information given
Blinding (performance bias and detection bias) All outcomes	Low risk	Quote: “The university investigational drug service encased medications in identical‐appearing capsules and dispensed them in weekly blister packs with specific instructions on day/ time for each dose.”
Incomplete outcome data (attrition bias) All outcomes	Unclear risk	The paper gives study retention figures, but does not specify whether they were lost to follow‐up.
Other bias	Low risk	None detected

**Table:** Górecka 2003

***Study characteristics***		
Methods	Study design: RCT Country: Poland Setting: smokers' clinic Recruitment: smokers with a diagnosis of COPD and failure to stop smoking with advice alone	
Participants	70 smokers with COPD 43% female; average age 56; average cigarettes per day 24	
Interventions	Bupropion, 300 mg/day for 7 weeks Nicotine patch, 15 mg/day for 8 weeks Common components: support at clinic visits at baseline, 2 weeks, EOT	
Outcomes	Smoking cessation: sustained abstinence at 1 year. Validated by CO < 10 ppm Adverse events: period of measurement unspecified	
Funding Source	None specified	
Author conflicts of interest	None specified	
Notes		
***Risk of bias***		
**Bias**	**Authors' judgement**	**Support for judgement**
Random sequence generation (selection bias)	Unclear risk	Randomisation method not described
Allocation concealment (selection bias)	Unclear risk	Allocation concealment not described
Blinding (performance bias and detection bias) All outcomes	High risk	Not described but presumably no blinding, as participants will have known assignment based on patch versus pill
Incomplete outcome data (attrition bias) All outcomes	Unclear risk	Not described
Other bias	Low risk	None detected

**Table:** Haggsträm 2006

***Study characteristics***		
Methods	Study design: RCT Country: Brazil Setting: smoking cessation clinic Recruitment: community volunteers	
Participants	156 smokers; FTND > 4; 70% female in placebo and nortriptyline arms, 59% in bupropion arm; average age 44; average cigarettes per day not specified	
Interventions	Bupropion, 300 mg/day for 60 days, placebo nortriptyline, TQD during week 2 Nortripytyline, 75 mg/day for 60 days, placebo bupropion Double placebo Common components: 6 x 15‐minute individual CBT, weekly then bi‐weekly	
Outcomes	Smoking cessation: continuous abstinence at 6 months (starting from TQD). Validated by CO ≤ 10 ppm at 3 months and 6 months Adverse events: measured for 26 weeks	
Funding Source	None specified	
Author conflicts of interest	None specified	
Notes		
***Risk of bias***		
**Bias**	**Authors' judgement**	**Support for judgement**
Random sequence generation (selection bias)	Unclear risk	Randomisation method not described
Allocation concealment (selection bias)	Unclear risk	Allocation concealment not described
Blinding (performance bias and detection bias) All outcomes	Low risk	Double‐blind, double‐dummy. "Both investigators and patients were blind to the treatment"
Incomplete outcome data (attrition bias) All outcomes	Unclear risk	Numbers lost to follow‐up not reported; all included as smokers in meta‐analysis
Other bias	Low risk	None detected

**Table:** Hall 1998

***Study characteristics***		
Methods	Study design: 2 x 2 factorial RCT Country: USA Setting: clinic Recruitment: community volunteers	
Participants	199 smokers, 33% had history of major depressive disorder (MDD); 55% female; average age 40; average cigarettes per day 21 to 25	
Interventions	Nortriptyline, titrated to therapeutic levels ‐ usually 75 mg/day to 100 mg/day, 12 weeks Placebo 2 x 2 factorial design. Alternative psychological Rxs were 10 sessions of CBT or 5 sessions of health education control. These arms were combined in analyses.	
Outcomes	Smoking cessation: prolonged abstinence at 1 year post‐EOT. Validated by CO at weeks 12, 24, 39, and 64 Adverse events: measured for 6 weeks	
Funding Source	National Instutute on Drug Abuse and Veterans Administration	
Author conflicts of interest	None specified	
Notes	There were no significant main or intervention effects based on whether participants had MDD or not and so these groups of participants were pooled.	
***Risk of bias***		
**Bias**	**Authors' judgement**	**Support for judgement**
Random sequence generation (selection bias)	Low risk	Computer randomisation, after stratification on history of MDD and number of cigarettes smoked
Allocation concealment (selection bias)	Low risk	Allocation generated at enrolment
Blinding (performance bias and detection bias) All outcomes	Unclear risk	Quote: "Medication was placebo controlled and double blind. Placebo and active drug were identical in appearance." However, no detail on who was blinded.
Incomplete outcome data (attrition bias) All outcomes	Low risk	30% did not complete treatment in placebo group and 17% in active group. Participants lost to follow‐up were assumed to be still smoking as is standard.
Other bias	Low risk	None detected

**Table:** Hall 2002

***Study characteristics***		
Methods	Study design: 3 x 2 factorial RCT Country: USA Setting: cessation research centre Recruitment: community volunteers	
Participants	220 smokers; 40% to 47% female; average age 37 to 43; average cigarettes per day 20 to 23	
Interventions	Bupropion, 300 mg/day, 12 weeks Nortriptyline, titrated to therapeutic levels, 12 weeks Placebo 3 x 2 factorial design. Alternative psychological interventions were (1) Medical Management (MM) (physician advice, S‐H, 10 to 20 minutes 1st visit, 5 minutes at 2, 6, and 11 weeks) or (2) Psychosocial Intervention (PI) (as for MM plus 5 x 90‐minute group sessions at 4, 5, 7, and 11 weeks)	
Outcomes	Smoking cessation: prolonged abstinence at 1 year (47 weeks post‐quit date). Validated by CO ≤ 10 ppm, urine cotinine ≤ 60 ng/mL Adverse events: measured for unspecified period	
Funding Source	National Institute on Drug Abuse, National Cancer Institute	
Author conflicts of interest	None specified	
Notes	No significant interaction between pharmacotherapy and behaviour therapy, so behavioural therapy arms combined in main analysis. Bupropion and nortriptyline compared to placebo and head‐to‐head. Levels of support compared for bupropion only, ppa rates used. Not included in behavioural support subgroup.	
***Risk of bias***		
**Bias**	**Authors' judgement**	**Support for judgement**
Random sequence generation (selection bias)	Unclear risk	Quote: "Participants were stratified by number of cigarettes smoked, sex and history of depression vs no history, and randomly assigned to 1 of the 6 experimental cells."
Allocation concealment (selection bias)	Low risk	Quote: "We encapsulated both drugs to maintain the patency of the bupropion formulation and to provide a blinded drug. All participants received capsules that were identical in number and appearance" but blinding of allocation not explicit.
Blinding (performance bias and detection bias) All outcomes	High risk	Double‐blind but participants informed about adverse effects of each drug and 87% of participants taking active drug guessed that they were (compared to 67% placebo group). Bupropion participants no more likely than nortriptyline participants to correctly identify which drug they had received.
Incomplete outcome data (attrition bias) All outcomes	Low risk	19% lost to follow‐up at 52 weeks. No significant difference across conditions. Included as smokers in meta‐analyses
Other bias	Low risk	None detected

**Table:** Hall 2004

***Study characteristics***		
Methods	Study design: 2 x 2 factorial RCT Country: USA Setting: clinic Recruitment: community volunteers	
Participants	160 smokers; 41% female; average age ~38; average cigarettes per day ~19	
Interventions	Nortriptyline, titrated to 50 ng/mL to 150 ng/mL (~75 mg to 100 mg) for 12 weeks, quit date week 5 Placebo 2 x 2 factorial design. Nortriptyline versus placebo and brief versus extended treatment. Brief treatment: nicotine patch for 8 weeks from quit date, and 5 group counselling sessions, total 7.5 hours Extended treatment: first 12 weeks as for brief treatment, then same dose continued to week 52 then tapered. Individual counselling every 4 weeks, total 3 hours to 4.5 hours. Phone counselling, total 40 to 80 minutes	
Outcomes	Smoking cessation: repeated 7 day ppa at 24 weeks, 36 weeks, 52 weeks. Validated by CO ≤ 10 ppm and urine cotinine ≤ 50 ng/mL at each point Adverse events: measured for 12 weeks	
Funding Source	National Institute on Drug Abuse	
Author conflicts of interest	None specified	
Notes	Factorial design, brief and extended treatment entered in meta‐analysis separately. In the active extended treatment arm, participants were still receiving nortriptyline at the time of final follow‐up.	
***Risk of bias***		
**Bias**	**Authors' judgement**	**Support for judgement**
Random sequence generation (selection bias)	Unclear risk	Randomisation stratified on cigarettes per day, prior NRT use, MDD history; method not specified
Allocation concealment (selection bias)	Unclear risk	Allocation concealment not described
Blinding (performance bias and detection bias) All outcomes	High risk	Quote: "participants given active drug were more likely to guess that they had received active drug (63%) than the placebo participants were to believe they were taking active drug (37%)"
Incomplete outcome data (attrition bias) All outcomes	Low risk	9% lost at week 52; included as smokers in meta‐analysis
Other bias	Low risk	None detected

**Table:** Hertzberg 2001

***Study characteristics***		
Methods	Study design: RCT Country: USA Setting: Veterans Affairs Medical Centre (VAMC) Recruitment: VAMC outpatient volunteers	
Participants	15 male veterans with post‐traumatic stress disorder; average age 50; average cigarettes per day 33	
Interventions	Bupropion, 300 mg/day, 12 weeks begun at least 1 week before TQD Placebo Common components: individual counselling pre‐quit, weeks 1, 2, 4, 8, 12	
Outcomes	Smoking cessation: prolonged abstinence at 6 months. Validated at weeks 2 and 8 by CO ≤ 10 ppm Adverse events: measured for 12 weeks	
Funding Source	GlaxoWellcome Inc, National Cancer Institute	
Author conflicts of interest	None specified	
Notes	2 of the successful quitters were taking bupropion at 6 months, prescribed after end of study	
***Risk of bias***		
**Bias**	**Authors' judgement**	**Support for judgement**
Random sequence generation (selection bias)	Unclear risk	Randomisation method not described
Allocation concealment (selection bias)	Unclear risk	Allocation concealment not described
Blinding (performance bias and detection bias) All outcomes	Unclear risk	Double‐blind, no further information provided
Incomplete outcome data (attrition bias) All outcomes	High risk	Uneven attrition between arms; very high percentage lost to follow‐up in placebo group. 30% of the participants receiving bupropion SR did not complete the full 12‐week trial; 80% of the placebo group failed to complete the trial and were considered to have resumed smoking.
Other bias	Low risk	None detected

**Table:** Hilberink 2010

***Study characteristics***		
Methods	Study design: cluster‐RCT Country: the Netherlands Setting: general practice Recruitment method: "A convenience sample was recruited in nine Dutch districts from general practices using one of four widely used general practice electronic record systems"	
Participants	667 smokers living with COPD randomised; 50% female, average age 59.6; average cigarettes per day 16.9	
Interventions	CN: intervention program with counselling and advice plus NRT CNB: intervention program with counselling and advice plus NRT plus additional bupropion Common components: "Individual support at the practice location (three visits)... The counseling activities were identical in both interventions."	
Outcomes	Smoking cessation: 52 week biochemically verified point prevalence abstinence; quote: "Self‐reported quitters were invited to produce a urine sample at the practice location. The sample was biochemically verified by cotinine levels (measured by radioimmunoassay). Patients with no 12‐month data, patients with more than 50 ng/mLin their urine and patients not providing a sample were considered to smoke."	
Funding Source	ZonMw: The Netherlands Organisation for Health Research and Development The Netherlands Asthma Foundation Pharmacia GlaxoSmithKline	
Author conflicts of interest	Quote: "None of the authors had a conflict of interest."	
Notes	Additional 'usual care' arm not relevant for this review.	
***Risk of bias***		
**Bias**	**Authors' judgement**	**Support for judgement**
Random sequence generation (selection bias)	Unclear risk	No information available
Allocation concealment (selection bias)	Unclear risk	No information available
Blinding (performance bias and detection bias) All outcomes	High risk	Not placebo‐controlled
Incomplete outcome data (attrition bias) All outcomes	Low risk	Attrition low and similar between arms
Other bias	High risk	This was a cluster‐RCT and analysis was not adjusted for clustering

**Table:** Hoch 2006

***Study characteristics***		
Methods	Study design: RCT Country: Germany Setting: 167 primary care settings in Munich and Dresden	
Participants	467 smokers randomised	
Interventions	CBT condition: 4 to 5 individual counselling sessions plus a cognitive‐behavioral self‐help manual were added Bupropion plus CBT NRT plus CBT (No further information on dose or duration of pharmacotherapy)	
Outcomes	Smoking cessation: prolonged abstinence at 52 weeks Adverse events: measured for 52 weeks	
Funding Source	Quote: "This study is sponsored by a grant of the German Federal Ministry of Education and Research (01 EB 0440‐0441, 01 EB 0142). During the preparatory phase of the study we received further support of GlaxoSmithKline GmbH & Co. KG (Munich), GlaxoSmithKline Consumer Health Care GmbH & Co KG (Bruhl) and Pharmacia GmbH (Erlangen). They provided the mandatory treatment manuals for use of their products."	
Author conflicts of interest	Not reported	
Notes	Only abstract and short study report available. There was an additional minimal intervention arm, not relevant to review as it is not matched in intensity for behavioural support.	
***Risk of bias***		
**Bias**	**Authors' judgement**	**Support for judgement**
Random sequence generation (selection bias)	Unclear risk	No information provided
Allocation concealment (selection bias)	Unclear risk	No information provided
Blinding (performance bias and detection bias) All outcomes	High risk	Unblinded
Incomplete outcome data (attrition bias) All outcomes	Low risk	Quote: "Overall, the retention rate across groups was 54.0%, with no significant between‐group differences"
Other bias	Low risk	None detected

**Table:** Holt 2005

***Study characteristics***		
Methods	Study design: RCT Country: New Zealand Setting: cessation clinic Recruitment: Maori community volunteers aged 16 to 70	
Participants	134 smokers; 72% female; average age 42/38	
Interventions	Bupropion, 300 mg/day for 7 weeks Placebo Common components: counselling at 3 clinic visits during medication and 3 monthly follow‐ups, motivational phone call 1 day before and 2 days after TQD	
Outcomes	Smoking cessation: continuous abstinence at 12 months. Validated by CO at each visit Adverse events: measured for 12 months	
Funding Source	GlaxoSmithKline	
Author conflicts of interest	P3 Research, the Wellington School of Medicine and Health Sciences, and the Medical Research Institute of New Zealand have all received research grants from GlaxoSmithKline and Novartis. SH and RB have received fees for consulting and reimbursement for attending symposia from GlaxoSmithKline and Novartis.	
Notes		
***Risk of bias***		
**Bias**	**Authors' judgement**	**Support for judgement**
Random sequence generation (selection bias)	Low risk	Randomisation using a computer‐generated code
Allocation concealment (selection bias)	Unclear risk	Not described
Blinding (performance bias and detection bias) All outcomes	Low risk	Quote: "Neither the study team nor the participant was aware of which treatment had been allocated until the end of the 12 month study period."
Incomplete outcome data (attrition bias) All outcomes	High risk	High and uneven loss to follow‐up, with less than half of placebo group followed up at 12 months. 36% lost in bupropion group and 52% in placebo at 12 months. "Participants who were lost to follow up were categorised as smokers ... often this was confirmed by family members or friends."
Other bias	Low risk	None detected

**Table:** Hurt 1997

***Study characteristics***		
Methods	Study design: RCT Country: USA Setting: multicentre Recruitment: community volunteers	
Participants	615 smokers; 55% female; average age 44; average cigarettes per day 27	
Interventions	Bupropion,100 mg/day for 7 weeks Bupropion, 150 mg/day Bupropion, 300 mg/day Placebo Common components: physician advice, S‐H materials, and brief individual counselling by study assistant at each visit	
Outcomes	Smoking cessation: prolonged abstinence at 12 months (starting from day 22). Validated by CO ≤ 10 ppm Adverse events: measured for 52 weeks	
Funding Source	GlaxoWellcome	
Author conflicts of interest	None specified	
Notes	300 mg compared with placebo in main analysis There was no evidence that history of major depression or alcoholism interacted with treatment condition or was associated with poorer outcomes. Prolonged abstinence rates at 12 months as supplied by GlaxoWellcome: 300 mg 21; 150 mg 23; placebo 15	
***Risk of bias***		
**Bias**	**Authors' judgement**	**Support for judgement**
Random sequence generation (selection bias)	Unclear risk	Randomised, stratified by site, method not described
Allocation concealment (selection bias)	Unclear risk	Allocation concealment not described
Blinding (performance bias and detection bias) All outcomes	Unclear risk	Quote: "Double‐blind" but no detail given on who was blinded
Incomplete outcome data (attrition bias) All outcomes	Unclear risk	Quote: "Subjects who missed a follow‐up visit were considered to be smoking.... The rate of completion of the study increased with the dose and was 57 percent, 65 percent, 64 percent, and 71 percent for the placebo, 100‐mg, 150‐mg, and 300‐mg groups, respectively..."
Other bias	Low risk	None detected

**Table:** Johns 2017

***Study characteristics***		
Methods	Study design: RCT Country: India Setting and recruitment method not specified	
Participants	300 participants randomised	
Interventions	Bupropion, 150 mg twice daily for 12 weeks Varenicline, 1 mg twice daily for 12 weeks Bupropion and varenicline, taken according to schedules above	
Outcomes	Smoking cessation: continuous abstinence at 6 months. Validated by CO Adverse events: period of measurement not detailed	
Funding Source	None specified	
Author conflicts of interest	None specified	
Notes	Abstract only. Insufficient data to add to meta‐analysis	
***Risk of bias***		
**Bias**	**Authors' judgement**	**Support for judgement**
Random sequence generation (selection bias)	Unclear risk	States trial was randomised, no further detail given
Allocation concealment (selection bias)	Unclear risk	No relevant information given
Blinding (performance bias and detection bias) All outcomes	Unclear risk	States only that the study was ‘double‐blind’, no further detail given
Incomplete outcome data (attrition bias) All outcomes	Unclear risk	No relevant information given
Other bias	Low risk	None detected

**Table:** Jorenby 1999

***Study characteristics***		
Methods	Study design: 2 x 2 factorial RCT Country: USA Setting: multicentre clinical trial units Recruitment: community volunteers	
Participants	893 smokers; 52% female; average age 43; average cigarettes per day 25	
Interventions	Nicotine patch and bupropion SR. Nicotine patch dosing and schedule: 21 mg/24 hours for 6 weeks, tapered for 2 weeks. Bupropion dosing and schedule was 300 mg for 9 weeks from 1 week before quit day Bupropion and placebo patch Nicotine patch and placebo tablets Placebo patch and placebo tablets Common components: brief (< 15 minutes) individual counselling session at each weekly assessment. One telephone call 3 days after quit day	
Outcomes	Smoking cessation: continuous ppa at 12 months. Validated by CO < 10 ppm at each clinic visit Adverse events: measured for unspecified period	
Funding Source	GlaxoWellcome	
Author conflicts of interest	"Dr Jorenby has organized medical education presentations sponsored by Glaxo Wellcome and SmithKline Beecham. Dr Leischow has served as a consultant for McNeil Consumer Products, Pharmacia and Upjohn, and Glaxo Wellcome and has organized medical education presentations sponsored by Glaxo Wellcome. Dr Nides has served as a consultant for Glaxo Wellcome, Novartis, and SmithKline Beecham and has organized medical education presentations sponsored by Glaxo Wellcome. Dr Rennard has served as a consultant for Glaxo Wellcome, Novartis, and SmithKline Beecham and has organized medical education presentations sponsored by Glaxo Wellcome. Dr Muramoto has organized medical education presentations sponsored by Glaxo Wellcome. Mr Daughton has served as a consultant for SmithKline Beecham and Hoechst Marion Roussel and has organized medical education presentations sponsored by Glaxo Wellcome and Hoechst Marion Roussel. Dr Fiore has served as a consultant for Novartis, Glaxo Wellcome, SmithKline Beecham, and McNeil Consumer Products and has organized medical education presentations sponsored by Novartis, Elan Pharma, Lederle Laboratories, Glaxo Wellcome, McNeil Consumer Products, and SmithKline Beecham. Dr Baker has served as a consultant for SmithKline Beecham and has organized medical education presentations sponsored by Elan Pharma and Glaxo Wellcome."	
Notes	Primary outcome for study was PP abstinence; this analysis uses continuous abstinence since quit day	
***Risk of bias***		
**Bias**	**Authors' judgement**	**Support for judgement**
Random sequence generation (selection bias)	Low risk	Quote: "The subjects were randomly assigned to one of four treatments with use of an unequal‐cell design... [but] Randomization was not balanced within sites."
Allocation concealment (selection bias)	Unclear risk	Allocation concealment method unclear
Blinding (performance bias and detection bias) All outcomes	Unclear risk	Quote: "Double‐blind" but no further detail provided
Incomplete outcome data (attrition bias) All outcomes	Low risk	Quote: "All subjects who discontinued treatment early or who were lost to follow‐up were classified as smokers." Approximately 20% left the study and provided no additional information. 15% stopped taking medication but participated in follow‐up assessments.
Other bias	Low risk	None detected

**Table:** Jorenby 2006

***Study characteristics***		
Methods	Study design: RCT Country: USA Setting: multicentre clinical trial units Recruitment: community volunteers	
Participants	683 smokers (in relevant arms), with prior exposure to bupropion excluded; 41% female; average age 42; average cigarettes per day 22	
Interventions	Bupropion 300 mg for 12 weeks plus placebo varenicline Varenicline 2 mg for 12 weeks plus placebo bupropion Placebo bupropion and placebo varenicline Common components: brief (< 10 minutes) individual counselling at each weekly assessment for 12 weeks and 5 follow‐up visits. One telephone call 3 days after quit day	
Outcomes	Smoking cessation: sustained abstinence at 12 months, from week 9. Validated by CO < 10 ppm at each clinic visit	
Funding Source	Pfizer Inc	
Author conflicts of interest	"Dr Jorenby reported receiving research support from Pfizer, Nabi Biopharmaceutical, Sanofi‐Aventis and consulting fees from Nabi Biopharmaceutical. Dr Hays reported receiving a research grant from Pfizer. Dr Rigotti reported receiving research grant funding and consulting fees from GlaxoSmithKline, which markets smoking cessation medications, and Pfizer and Sanofi‐Aventis, which are developing smoking cessation medications. Dr Rigotti also reported receiving consulting fees from Merck, which is developing smoking cessation medications."	
Notes	Bupropion was an active control for varenicline. Bupropion versus placebo and bupropion versus varenicline comparisons contribute to the review.	
***Risk of bias***		
**Bias**	**Authors' judgement**	**Support for judgement**
Random sequence generation (selection bias)	Low risk	Quote: "Randomization was completed centrally by using a computer‐generated list and sites used an electronic system to assign participants to treatment."
Allocation concealment (selection bias)	Low risk	Quote: "Folders [containing medication or placebo] for all participants (regardless of treatment assignment) were identical throughout the treatment phase including a period of dose titration (week 1) and treatment at the target dose (weeks 2‐12)."
Blinding (performance bias and detection bias) All outcomes	Unclear risk	Quote: "in a double‐blind manner," no further information provided
Incomplete outcome data (attrition bias) All outcomes	Low risk	Over the period of treatment and follow‐up 14% of those receiving varenicline were lost to follow‐up; 14% randomised to bupropion lost to follow‐up; 16% of the placebo group were lost to follow‐up. "Participants whose smoking status was unknown or whose carbon monoxide level was higher than 10 ppm were classified as smoking during both the treatment phase and follow‐up."
Other bias	Low risk	None detected

**Table:** Kahn 2012

***Study characteristics***		
Methods	Study design: RCT Country: USA Setting: clinics Recruitment: community	
Participants	246 smokers; 49% female; average age 46; average cigarettes per day 22	
Interventions	Selegiline patch (6 mg/24 hours) for 9 weeks, starting 7 days before TQD Placebo patch, same schedule as selegiline Common components: 9 weekly individual counselling sessions of approximately 10 minutes each	
Outcomes	Smoking cessation: prolonged abstinence at 6 months (continuous from week 6 onwards). Validated by CO < 9 ppm Adverse events: measured for 26 weeks	
Funding Source	National Institutes of Health, National Institute on Drug Abuse	
Author conflicts of interest	None specified	
Notes	Some additional information on study characteristics provided by author Mean compliance rates 91.6% and 91.3% for the selegiline and placebo groups	
***Risk of bias***		
**Bias**	**Authors' judgement**	**Support for judgement**
Random sequence generation (selection bias)	Unclear risk	Quote: "Adaptive randomization," method not reported
Allocation concealment (selection bias)	Unclear risk	Not specified
Blinding (performance bias and detection bias) All outcomes	Unclear risk	Quote: "Double‐blind," no further details provided
Incomplete outcome data (attrition bias) All outcomes	Low risk	70% placebo and 74% selegiline followed up at 12 months
Other bias	Low risk	None detected

**Table:** Kalman 2011

***Study characteristics***		
Methods	Study design: RCT Country: USA Setting: not specified Recruitment: Veterans Administration Medical Center	
Participants	143 smokers with 2 to 12 months of alcohol abstinence, with history of alcohol abuse or dependence; mean age 49; 17% female; average cigarettes per day 20.8; mean FTND 5.9	
Interventions	Bupropion, 8 weeks (started 1 week before TQD, first 3 days 150 mg/day, rest of period 2 x 150 mg/day) Placebo, same schedule as above Common components: nicotine patch (7 weeks starting on TQD; 21 mg weeks 1 to 4, 14 mg weeks 5 to 6, 7 mg week 7) and 8 weekly counselling sessions starting 1 week before TQD (one‐to‐one sessions based on cognitive behavioural therapy and motivational interviewing)	
Outcomes	Smoking cessation: prolonged abstinence at 24 weeks (no smoking after first 2 weeks after TQD). Validated by salivary cotinine ≤ 15 ng/mL Adverse events: measured for unspecified period	
Funding Source	National Institute of Drug Abuse, National Institute on Alcohol Abuse and Alcoholism	
Author conflicts of interest	None specified	
Notes	Number of participants who quit calculated from percentages provided	
***Risk of bias***		
**Bias**	**Authors' judgement**	**Support for judgement**
Random sequence generation (selection bias)	Unclear risk	Quote: "Urn randomization"; no further details provided
Allocation concealment (selection bias)	Unclear risk	Not described
Blinding (performance bias and detection bias) All outcomes	Unclear risk	Quote: "Double‐blind" but no detail on who was blinded in terms of study staff, including counsellors. "Both medication groups performed at the chance level in judging medication assignment."
Incomplete outcome data (attrition bias) All outcomes	Low risk	13 participants who dropped out prior to receiving medication, not included in denominators. Further 18% intervention and 14% control lost at 24 weeks, counted as smoking in analyses.
Other bias	Low risk	None detected

**Table:** Karam‐Hage 2011

***Study characteristics***		
Methods	Study design: RCT Country: USA Setting: University of Michigan outpatient addictions clinic Recruitment method: patients admitted to the outpatient intensive treatment programme	
Participants	Alcohol‐ and nicotine‐dependent patients 11 participants randomised; 55% female; average age 19.7; average cigarettes per day 1 pack; mean FTND 4.8	
Interventions	Bupropion, 150 mg once daily for 7 days, then twice daily for 7 weeks Placebo, same scheduling as bupropion Common components: minimal smoking cessation counselling and booklet “You Can Quit Smoking”	
Outcomes	Smoking cessation: 8 weeks ‐ too short a follow‐up to be considered for this outcome as part of our review Adverse events: measured for 8 weeks	
Funding Source	University of Michigan's General Clinical Research Center (GCRC) Grant # MO1 RR00042	
Author conflicts of interest	None specified	
Notes		
***Risk of bias***		
**Bias**	**Authors' judgement**	**Support for judgement**
Random sequence generation (selection bias)	Unclear risk	No relevant information given
Allocation concealment (selection bias)	Unclear risk	No relevant information given
Blinding (performance bias and detection bias) All outcomes	Unclear risk	No relevant information given
Incomplete outcome data (attrition bias) All outcomes	Low risk	Dropout rates are as follows: 1/5 placebo; 1/6 bupropion
Other bias	Low risk	None detected

**Table:** Killen 2000

***Study characteristics***		
Methods	Study design: RCT Country: USA Setting: clinic Recruitment: advertisements	
Participants	224 smokers; 46% female; average age 46; average cigarettes per day 26	
Interventions	Nicotine patch and paroxetine. Nicotine patch for 24 hours, 21 mg, 8 weeks. Paroxetine at 20 mg for 9 weeks, including tapering Nicotine patch and paroxetine. 40 mg paroxetine. Patch as above Nicotine patch and placebo paroxetine Common components: self‐help manual and 15‐minute behavioural counselling at weeks 1 and 4	
Outcomes	Smoking cessation: 7‐day ppa at 10 weeks, 26 weeks, and 6 months. Validated by CO < 9 ppm and saliva cotinine < 20 ng/mL at each visit Adverse events: measured for 26 weeks	
Funding Source	University of California Tobacco‐Related Disease Research Program, SmithKline Beecham	
Author conflicts of interest	None specified	
Notes	40 mg and 20 mg dose pooled in meta‐analysis from 2009. 20/75 quit on 40 mg, 15/75 on 20 mg	
***Risk of bias***		
**Bias**	**Authors' judgement**	**Support for judgement**
Random sequence generation (selection bias)	Unclear risk	Randomisation method not described
Allocation concealment (selection bias)	Unclear risk	Not described
Blinding (performance bias and detection bias) All outcomes	Unclear risk	Quote: "Double‐blind" but unclear who exactly was blinded
Incomplete outcome data (attrition bias) All outcomes	Unclear risk	Quote: "Those failing to provide confirmation [of smoking status] were reclassified as smokers." Number lost to follow‐up not reported
Other bias	Low risk	None detected

**Table:** Killen 2004

***Study characteristics***		
Methods	Study design: RCT Country: USA Setting: continuation high schools Recruitment: adolescents at schools	
Participants	211 adolescent smokers, at least 1 failed quit attempt; 31% female; average age 17; average cigarettes per day 15	
Interventions	Bupropion and nicotine patch. Bupropion at 150 mg for 9 weeks from 1 week before TQD. Nicotine patch for 8 weeks Placebo and nicotine patch Common components: weekly 45‐minute group sessions, skills training	
Outcomes	Smoking abstinence: 7 day ppa at 6 months. Validated by saliva cotinine < 20 ng/mL at 6 months (CO at EOT) Adverse events: measured for unspecified period	
Funding Source	National Cancer Institute. GlaxoSmithKline provided medication	
Author conflicts of interest	None specified	
Notes	Low compliance with both bupropion and patch therapy	
***Risk of bias***		
**Bias**	**Authors' judgement**	**Support for judgement**
Random sequence generation (selection bias)	Unclear risk	Randomisation method not described
Allocation concealment (selection bias)	Unclear risk	Allocation concealment not described
Blinding (performance bias and detection bias) All outcomes	Low risk	Quote: "Double‐blind." Though further details not provided, assessment of blind suggests it was successful (30% placebo and 31% bupropion correctly guessed assignment)
Incomplete outcome data (attrition bias) All outcomes	Low risk	38% bupropion and 35% placebo lost at 6 months, included in analysis
Other bias	Low risk	None detected

**Table:** Killen 2010

***Study characteristics***		
Methods	Study design: RCT Country: USA Setting: community Recruitment: radio, newspapers, community website, and notices distributed via local organisations	
Participants	243 smokers, 18 to 65 years old. 30% female; average age 45; average cigarettes per day 19	
Interventions	Selegiline patch. 8 weeks, 6 mg/24 hours, starting on TQD Placebo. Same schedule as above Common components: 9 sessions of individual counselling to develop cognitive and behavioural skills to resist urges to smoke	
Outcomes	Smoking cessation: 7‐day ppa at 12 months. Validated by CO < 10 ppm Adverse events: measured for unspecified period	
Funding Source	National Institute on Drug Abuse. Medication and matching placebo provided by Somerset Pharmaceuticals, Inc.	
Author conflicts of interest	None specified	
Notes		
***Risk of bias***		
**Bias**	**Authors' judgement**	**Support for judgement**
Random sequence generation (selection bias)	Low risk	Random number generator
Allocation concealment (selection bias)	Low risk	Participants assigned sequential ID numbers corresponding with drug “pre‐packaged and labelled by ID only at an off‐site location by an individual who had no association with the participants.”
Blinding (performance bias and detection bias) All outcomes	Low risk	Quote: “Treatment assignment was concealed from staff and both research staff and participants were blind to week 52.” Assessment of blinding in participants and study staff suggests it was successful
Incomplete outcome data (attrition bias) All outcomes	Low risk	87% followed up at 12 months, same in both arms. Missing counted as smokers in meta‐analysis
Other bias	Low risk	None detected

**Table:** Kumar 2020

***Study characteristics***		
Methods	Study design: cluster‐randomised controlled trial Country: India Setting: Designated Microscopy Centres (DMCs) of the Revised National Tuberculosis Control Program (RNTCP) centres in two districts of Tamilnadu Recruitment method: people attending the DMCs	
Participants	517 smokers with a diagnosis of smear‐positive TB were randomised; 0% female, average age 46; average cigarettes per day 13.1	
Interventions	Clinical guideline modules Bupropion SR along with standard counselling, 100 mg tablet was started once daily for a week and then twice daily for 7 weeks Common components: in‐person behavioural support following the '5As' approach	
Outcomes	Smoking cessation: 7‐day point prevalence at 26 weeks. Validated by CO < 10 ppm	
Funding Source	Quote: "The study was funded by The United States Agency for International Development (USAID) through the World Health Organization(WHO) under the Model DOTS Project (MDP) reference number: 2013/313079‐0"	
Author conflicts of interest	Quote: "No conflicts of interests reported"	
Notes	Additional enhanced counselling arm, but not relevant for this review as no pharmacotherapy was offered, and intensity of the counselling differed from the other two groups.	
***Risk of bias***		
**Bias**	**Authors' judgement**	**Support for judgement**
Random sequence generation (selection bias)	Unclear risk	No information apart from, quote: "each DMC as a cluster unit for randomization...Thirty‐six RNTCP centres (DMCs) from two districts of Tamilnadu, (18 each from Villupuram and Kanchipuram districts) were randomly selected to receive any one of the interventions"
Allocation concealment (selection bias)	Unclear risk	As above
Blinding (performance bias and detection bias) All outcomes	High risk	Study was unblinded, with no placebo or active pharmacotherapy treatment in the control arms.
Incomplete outcome data (attrition bias) All outcomes	Low risk	25.6% of the standard counselling group and 21.1% of the bupropion plus standard counselling group were lost to follow‐up
Other bias	High risk	This was a cluster‐RCT and baseline characteristics were unbalanced between arms: participants in the standard counselling arm appear to have been more highly addicted than bupropion arm, i.e. smoking more, for longer. Dependence, number of cigarettes smoked per day, all higher in control than intervention. There was also no evidence that analysis has been adjusted for clustering.

**Table:** Levine 2010

***Study characteristics***		
Methods	Study design: 2 x 2 factorial RCT Country: USA Setting: not specified Recruitment: community volunteers	
Participants	349 weight‐concerned women smokers; average age 42; average cigarettes per day 21; mean FTND 5.2	
Interventions	Bupropion SR. 26 weeks. 150 mg/day for first 2 days and 300 mg/day for remainder of treatment Placebo, same schedule Counselling conditions Standard cessation counselling Standard cessation counselling plus material on weight concerns Common components: 12 x 90‐minute group counselling sessions delivered over 3 months	
Outcomes	Smoking cessation: prolonged abstinence at 12 months. Validated by CO ≤ 8 ppm and salivary cotinine ≤ 15 µg Adverse events: measured for 26 weeks	
Funding Source	National Institute on Drug Abuse. Medication supplied by GlaxoSmithKline	
Author conflicts of interest	Dr Marcus has served as a consultant to GlaxoSmithKline and Sanofi‐Aventis. Dr Perkins has served as a consultant for GlaxoSmithKline	
Notes	Counselling arms combined in analyses (same intensity, just differed in content). N abstinent calculated from percentages given	
***Risk of bias***		
**Bias**	**Authors' judgement**	**Support for judgement**
Random sequence generation (selection bias)	Unclear risk	Blocked randomisation; method of sequence generation not reported
Allocation concealment (selection bias)	Unclear risk	Not reported
Blinding (performance bias and detection bias) All outcomes	Unclear risk	Quote: "Double‐blind"; no further information provided
Incomplete outcome data (attrition bias) All outcomes	High risk	Over half lost to follow‐up at 12 months. 48% followed up overall, similar rates between groups
Other bias	Low risk	None detected

**Table:** McCarthy 2008

***Study characteristics***		
Methods	Study design: 2 x 2 factorial RCT Country: USA Setting: cessation clinic Recruitment: community volunteers	
Participants	463 smokers; 50% female; average age 36 to 41; average cigarettes per day 22	
Interventions	Bupropion SR 300 mg for 8 weeks Placebo Counselling conditions 8 x 10‐minute sessions: 2 pre‐quit, TQD, 5 over 4 weeks Psychoeducation about medication, support and encouragement. Same number of sessions, 80 minutes less contact time	
Outcomes	Smoking cessation: 7 day ppa at 12 months. Validated by CO ≤ 10 ppm. Prolonged self‐reported abstinence also assessed Adverse events: measured for 9 weeks	
Funding Source	National Cancer Institute, National Instutute on Drug Abuse. GlaxoSmithKline provided placebo medication	
Author conflicts of interest	"Douglas E Jorenby has received research support from Nabi Biopharmaceutical and Pfizer, Inc. and consulting fees from Nabi Biopharmaceutical. Saul Shiffman serves as consultant to GlaxoSmithKline Consumer Healthcare on an exclusive basis regarding over‐the‐counter smoking cessation products and also is a partner in a company that is developing a new nicotine medication. He is a cofounder of invivodata, inc., which provides electronic diary services for clinical research. In 1998 the University of Wisconsin appointed Dr Fiore to a named Chair, made possible by an unrestricted gift to the university from GlaxoWellcome. GlaxoSmithKline provided complimentary active and placebo medication used in this study."	
Notes	Counselling conditions combined in main analysis, entered separately in subgroup analysis by intensity. Psychoeducation arms placed in multisession individual counselling subgroup due to high level of contact received, though not classified as counselling in paper.	
***Risk of bias***		
**Bias**	**Authors' judgement**	**Support for judgement**
Random sequence generation (selection bias)	Low risk	Random number table
Allocation concealment (selection bias)	Low risk	Staff who screened and enrolled participants were unaware of the experimental condition to be assigned
Blinding (performance bias and detection bias) All outcomes	Low risk	Double‐blind (for medication). "Research staff who interacted with participants were blind to participants' medication condition assignment."
Incomplete outcome data (attrition bias) All outcomes	Low risk	171 (37%) failed to attend quit date visit or lost to follow‐up, similar across groups, included in ITT analysis
Other bias	Low risk	None detected

**Table:** Minami 2014

***Study characteristics***		
Methods	Study design: RCT Country: USA Setting: community Recruitment method: "recruited from local community"	
Participants	People with elevated depressive symptoms, as indicated by a Center for Epidemiologic Studies Depression Scale (CES‐D) score > 6 206 participants randomised; 48% female; average age 43; average cigarettes per day 21; mean FTND 5.5	
Interventions	Fluoxetine. 20 mg each morning, 8 weeks prior to target quit date and 8 weeks following Placebo. According to the schedule detailed above Common components: 8‐week supply of nicotine patches and brief counselling, totalling a maximum of 150 minutes	
Outcomes	Smoking cessation: 8 weeks ‐ too short a follow‐up for this outcome to be considered as part of this review Adverse events: measured for 8 weeks pre‐quit, although whether they recorded post‐quit is not clearly specified	
Funding Source	NIDA	
Author conflicts of interest	Dr Price reports receiving grant/research support from Medtronic, Neuronetics, NIH, HRSA, and NeoSync; serving on an advisory panel for Abbott; and serving as a consultant for Wiley, Springer, Qatar National Research Fund, and Abbott.	
Notes	The way that side effect data were reported could not be entered into the meta‐analysis, but has been included narratively.	
***Risk of bias***		
**Bias**	**Authors' judgement**	**Support for judgement**
Random sequence generation (selection bias)	Low risk	Quote: "urn randomization to balance the groups on gender, depressive symptoms (CES‐D ≥16), and nicotine dependence (FTND ≥ 7)."
Allocation concealment (selection bias)	Unclear risk	No relevant information given
Blinding (performance bias and detection bias) All outcomes	Low risk	Quote: “assignment was double‐blind, such that neither participants nor study staff (including physicians, research assistants, and counselors) were aware of whether the participant was taking fluoxetine or placebo."
Incomplete outcome data (attrition bias) All outcomes	Unclear risk	No relevant information given
Other bias	Low risk	None detected

**Table:** Moreno‐Coutino 2015

***Study characteristics***		
Methods	Study design: RCT Country: Mexico Setting: smoking cessation clinic Recruitment method: people seeking smoking cessation treatment at clinic	
Participants	Heavy smokers with minimal/mild depressive symptomatology 60 participants randomised; 38% female; average age 45; average cigarettes per day 18.2; mean FTND 4.7	
Interventions	Bupropion. 150 mg once daily for 2 weeks prior to target quit date, then 150 mg twice daily from 1 week prior to target quit date until 4 months of treatment Nicotine patch. 21 mg starting 2 weeks before target quit date. 4 weeks at 21 mg following target quit date, 14 mg for 2 weeks, then 7 mg for two weeks Bupropion and nicotine patch. Given according to schedules above Common components: 4 individual in‐person CBT sessions (over 4 weeks, 2 pre‐quit and 2 post‐quit), plus 0.1 mg low nicotine cigarettes	
Outcomes	Smoking cessation: at 12.5 months Adverse events: period of measurement not specified	
Funding Source	Mexican National University Macro‐project in Addictions	
Author conflicts of interest	None specified	
Notes	Not included in meta‐analysis because abstinence data not reported by group	
***Risk of bias***		
**Bias**	**Authors' judgement**	**Support for judgement**
Random sequence generation (selection bias)	High risk	Quote: “Those who agreed to continue in the study, entered the raffle (three different color balls in a dark box) to assign a treatment setting, and were evaluated."
Allocation concealment (selection bias)	High risk	Quote: “Those who agreed to continue in the study, entered the raffle (three different color balls in a dark box) to assign a treatment setting, and were evaluated."
Blinding (performance bias and detection bias) All outcomes	High risk	Quote: “evaluations and treatments were conducted by clinical psychologists who were not blind to the study."
Incomplete outcome data (attrition bias) All outcomes	High risk	High dropout rate from each group (> 50%). Significantly more dropouts from NRT only arm
Other bias	Low risk	None detected

**Table:** Muramoto 2007

***Study characteristics***		
Methods	Study design: RCT Country: USA Setting: research clinic Recruitment: adolescent community volunteers	
Participants	312 adolescents (14 to 17); 46% females; median age 16; median cigarettes per day 11	
Interventions	Bupropion, 300 mg for 7 weeks Bupropion, 150 mg for 7 weeks Placebo Common components: brief (10‐ to 20‐minutes) individual counselling session pre‐quit and at each weekly assessment	
Outcomes	Smoking cessation: 7‐day ppa at 6 months. Validated by CO < 10 ppm (cotinine at weeks 2 and 6 only) Adverse events: measured for 26 weeks	
Funding Source	National Cancer Institute, The Robert Wood Johnson Foundation, GlaxoSmithKline	
Author conflicts of interest	Dr Muramoto has received research contracts from GlaxoSmithKline, Pfizer, and Sanofi‐Aventis and is a speaker for Pfizer. Dr Leischow is a speaker and consultant for Pfizer, and at the time this study was conducted he was receiving research support from GlaxoSmithKline.	
Notes	300 mg arm contributes to main analysis. 2/105 quit in 150 mg group	
***Risk of bias***		
**Bias**	**Authors' judgement**	**Support for judgement**
Random sequence generation (selection bias)	Low risk	Quote: "Active study medication and identical‐appearing placebo were prepackaged into 3 sets of identical‐appearing blister cards in accordance with a computer‐generated randomization list."
Allocation concealment (selection bias)	Low risk	Quote: "... a research assistant assigned the subject the next treatment number (and associated blister cards) in sequence."
Blinding (performance bias and detection bias) All outcomes	Low risk	Quote: "Study subjects and researchers remained blind to treatment group assignment throughout the study." "9.6% in the 300 mg group accurately guessed their treatment assignment. Across all treatment groups, there were no significant differences in the proportion of subjects who accurately guessed their treatment group."
Incomplete outcome data (attrition bias) All outcomes	Low risk	Slightly higher loss to follow‐up/declined further participation in placebo group (30%) than active arms (18%). ITT analysis
Other bias	Low risk	None detected

**Table:** Myles 2004

***Study characteristics***		
Methods	Study design: RCT Country: Australia Setting: preoperative clinic Recruitment: smokers awaiting surgery	
Participants	47 smokers expected to undergo surgery within 8 to 14 weeks; 34% female; average age 45/40; 49% smoked 21 to 30 cigarettes per day	
Interventions	Bupropion. 300 mg for 7 weeks Placebo Common components: advice at baseline, 1 phone call 2 to 4 days after TQD. Low intensity	
Outcomes	Smoking cessation: 28 day ppa at 6 months. Validated by CO ≤ 10 ppm Adverse events: not clearly specified	
Funding Source	Alfred Hospital Research Trust, GlaxoWellcome	
Author conflicts of interest	None specified	
Notes	More dropouts in placebo group. Only 20 had surgery	
***Risk of bias***		
**Bias**	**Authors' judgement**	**Support for judgement**
Random sequence generation (selection bias)	Low risk	Participants were randomly allocated from a table of random numbers into one of two groups: active (bupropion) or placebo (identical appearance)
Allocation concealment (selection bias)	Unclear risk	Not described
Blinding (performance bias and detection bias) All outcomes	Unclear risk	Quote: "Double‐blind," no further detail provided
Incomplete outcome data (attrition bias) All outcomes	Low risk	17% lost to follow‐up in the bupropion group; 9% lost to follow‐up in the placebo group. "Patients lost to follow‐up were assumed to still be smoking."
Other bias	Low risk	None detected

**Table:** NCT00132821

***Study characteristics***		
Methods	Study design: 2 x 2 factorial RCT Country: USA Setting: sleep clinic Recruitment: not specified	
Participants	59 participants enrolled; smoking at least 20 cigarettes per day. No further participant characteristics given	
Interventions	Starting 1 week prior to quit day Bupropion. 150 mg for 3 days and 300 mg for 60 days Placebo bupropion Added on quit day Nicotine patch (21 mg for 6 weeks, 14 mg for 1 week, and 7 mg for 1 week) Placebo nicotine patch	
Outcomes	Smoking cessation: at 12 months (no definition of abstinence given). Validated by CO Adverse events: not specified whether adverse events were recorded	
Funding Source	National Institute on Drug Abuse	
Author conflicts of interest	None specified	
Notes	Study detailed in trials registry only and results not reported. Attempt to contact the investigator for further information was unsuccessful	
***Risk of bias***		
**Bias**	**Authors' judgement**	**Support for judgement**
Random sequence generation (selection bias)	Unclear risk	No relevant information given
Allocation concealment (selection bias)	Unclear risk	No relevant information given
Blinding (performance bias and detection bias) All outcomes	Unclear risk	No relevant information given
Incomplete outcome data (attrition bias) All outcomes	Unclear risk	No relevant information given
Other bias	Low risk	None detected

**Table:** NCT00308763

***Study characteristics***		
Methods	Study design: RCT Country: USA Setting: not specified Recruitment: not specified	
Participants	594 younger, low‐income, and minority smokers enrolled. No further participant characteristics given	
Interventions	Nicotine patch and placebo bupropion SR. If smoking > 20 cigarettes per day, participants were initially given 21 mg patch; 10 to 19 cigarettes per day 14 mg patch; 5 to 9 cigarettes per day 7 mg patch. If initially placed on the 21 mg patch: 21 mg patch for 4 weeks, 14 mg patch for 4 weeks, 7 mg patch for 2 weeks; if initially placed on 14 mg patch: 14 mg patch for 6 weeks, 7 mg patch for 4 weeks; if initially placed on 7 mg patch: 7 mg patch for 10 weeks. Bupropion scheduling as below. Placebo nicotine patch and bupropion SR. Bupropion titrated to 150 mg, then 150 mg daily for approximately 11 weeks. Placebo patch scheduled as above. Nicotine patch and bupropion SR. Same scheduling as above.	
Outcomes	Smoking cessation: at 12 months (no definition of abstinence given). Validated by CO and saliva cotinine Adverse events: not specified whether adverse events were recorded	
Funding Source	National Institutes of Health (R01HL066025)	
Author conflicts of interest	None specified	
Notes	Study detailed in trials registry only and results not reported. Attempt to contact the investigator for further information was unsuccessful	
***Risk of bias***		
**Bias**	**Authors' judgement**	**Support for judgement**
Random sequence generation (selection bias)	Unclear risk	No relevant information given
Allocation concealment (selection bias)	Unclear risk	No relevant information given
Blinding (performance bias and detection bias) All outcomes	Unclear risk	No relevant information given
Incomplete outcome data (attrition bias) All outcomes	Unclear risk	No relevant information given
Other bias	Low risk	None detected

**Table:** NCT00495352

***Study characteristics***		
Methods	Study design: RCT Country: Taiwan Setting: not specified Recruitment: not specified	
Participants	360 motivated psychiatric outpatients with schizophrenia enrolled. No further participant characteristics detailed	
Interventions	High‐dose NRT Low‐dose NRT Bupropion	
Outcomes	Smoking cessation: at 8 weeks, too short a follow‐up for consideration in this review Adverse events: not specified whether adverse events were recorded	
Funding Source	Yu‐Li Hospital; Department Of Health, Executive Yuan, ROC (Taiwan); National Health Research Institutes, Taiwan	
Author conflicts of interest	None specified	
Notes	Study detailed in trials registry only and results not reported. Attempt to contact the investigator for further information was unsuccessful	
***Risk of bias***		
**Bias**	**Authors' judgement**	**Support for judgement**
Random sequence generation (selection bias)	Unclear risk	No relevant information given
Allocation concealment (selection bias)	Unclear risk	No relevant information given
Blinding (performance bias and detection bias) All outcomes	Unclear risk	No relevant information given
Incomplete outcome data (attrition bias) All outcomes	Unclear risk	No relevant information given
Other bias	Low risk	None detected

**Table:** NCT00578669

***Study characteristics***		
Methods	Study design: RCT Country: USA Setting and recruitment method not specified	
Participants	Participants had elevated depression symptoms. 206 participants randomised; 48% female; average age 44; average cigarettes per day 21; mean FTND 5.7	
Interventions	Fluoxetine. 20 mg once daily, 8 weeks prior to target quit date and 8 weeks thereafter Placebo. Given according to schedule detailed above Common components: nicotine patch as well as "standard smoking cessation treatment"	
Outcomes	Smoking cessation: 7‐day ppa at 12 months. Validated by CO and saliva cotinine Adverse events: measured for a period of one year	
Funding Source	None specified	
Author conflicts of interest	None specified	
Notes		
***Risk of bias***		
**Bias**	**Authors' judgement**	**Support for judgement**
Random sequence generation (selection bias)	Unclear risk	No relevant information given
Allocation concealment (selection bias)	Unclear risk	No relevant information given
Blinding (performance bias and detection bias) All outcomes	Unclear risk	Placebo‐controlled, but no further information on blinding provided
Incomplete outcome data (attrition bias) All outcomes	Unclear risk	Clinical trial registry implies 100%, but not explicitly, so we concluded there was insufficient information given
Other bias	Low risk	None detected

**Table:** NCT01406223

***Study characteristics***		
Methods	Study design: RCT Country: USA Setting and recruitment method not specified	
Participants	76 participants randomised; 53% female; average age 38.8	
Interventions	Bupropion and varenicline. Bupropion was given 150 mg once daily for the first week, then twice daily for remainder of the 12‐week treatment period. Varenicline was administered 0.5 mg once daily starting one week preceding the target quit date, 0.5 mg twice daily for the remaining 4 days of that week, then 1 mg twice daily of the remainder of the 12‐week treatment period. Placebo and varenicline. Given according to the relevant schedules detailed above.	
Outcomes	Smoking cessation: not specified Adverse events: measured for 13‐week treatment period	
Funding Source	Not specified	
Author conflicts of interest	Not specified	
Notes		
***Risk of bias***		
**Bias**	**Authors' judgement**	**Support for judgement**
Random sequence generation (selection bias)	Unclear risk	No relevant information given
Allocation concealment (selection bias)	Unclear risk	No relevant information given
Blinding (performance bias and detection bias) All outcomes	Unclear risk	Stated that the study was placebo‐controlled and there was “double masking”, but no further detail is given
Incomplete outcome data (attrition bias) All outcomes	High risk	Dropout rates were as follows: 12/18 varenicline; 18/20 varenicline
Other bias	Low risk	None detected

**Table:** Niaura 2002

***Study characteristics***		
Methods	Study design: RCT Country: USA Setting: 16 clinical trial centres Recruitment: community volunteers	
Participants	989 non‐depressed smokers; 61% female; average age 42; average cigarettes per day 28	
Interventions	Fluoxetine. 30 mg for 10 weeks, starting 2 weeks before TQD Fluoxetine. 60 mg for 10 weeks, starting 2 weeks before TQD Placebo Common components: 9 sessions (60 to 90 minutes) individual CBT. Included coping skills, stimulus control techniques, and relapse prevention	
Outcomes	Smoking cessation: multiple ppa at 32 weeks from TQD. Validated by saliva cotinine < 20 ng/mL at each visit Adverse events: measured for 6 months	
Funding Source	Eli Lilly and Company	
Author conflicts of interest	None specified	
Notes	Originally based on abstract and data from authors. From 2002, based on full report. Numbers quit derived from rounded quit rates (10% quit in each group)	
***Risk of bias***		
**Bias**	**Authors' judgement**	**Support for judgement**
Random sequence generation (selection bias)	Unclear risk	Randomisation method not described
Allocation concealment (selection bias)	Unclear risk	Not described
Blinding (performance bias and detection bias) All outcomes	Unclear risk	Double‐blind but further detail not provided
Incomplete outcome data (attrition bias) All outcomes	Low risk	Missing data in treatment phase addressed, but unclear whether missing data in follow‐up phase addressed. At 12 months, 42% missing data, similar across all arms; missing data counted as smokers in our analyses.
Other bias	Low risk	None detected

**Table:** Nides 2006

***Study characteristics***		
Methods	Study design: RCT Country: USA Setting: 5 clinical sites Recruitment: volunteers (phase II study)	
Participants	638 smokers (255 in relevant arms, including 2 bupropion and 4 placebo who did not start medication); 51% female; average age 41; average cigarettes per day 20	
Interventions	Bupropion, 300 mg for 7 weeks Varenicline, 2 mg for 7 weeks (other dose regimens not used in review) Placebo Common components: up to 10 minutes counselling at 7 weekly clinic visits, plus at 12 weeks and 24 weeks	
Outcomes	Smoking cessation: continuous abstinence at 12 months (starting from week 4). Validated by CO Adverse events: measured for 11 weeks	
Funding Source	Pfizer	
Author conflicts of interest	"Dr Nides has received research grants, consulting fees, and honoraria from Pfizer, Sanofi‐Aventis, and GlaxoSmithKline. Dr Oncken has received research grants, consulting fees, and honoraria from Pfizer; received, at no cost, nicotine replacement and placebo products from GlaxoSmithKline for smoking cessation studies; and received honoraria from Pri‐Med. Dr Gonzales reports having received research contracts from Pfizer, Sanofi‐Aventis, GlaxoSmithKline and Nabi Biopharmaceuticals; consulting fees and honoraria from Pfizer, Sanofi‐Aventis, and GlaxoSmithKline; and owning 5 shares of Pfizer stock. Dr Rennard has had or currently has a number of relationships with companies that provide product and/or services relevant to outpatient management of chronic obstructive pulmonary disease. These relationships include serving as a consultant (Adams, Almirall, Altana, Array Biopharma, AstraZeneca, Aventis, Biolipox, Centocor, Dey, Critical Therapeutics, GlaxoSmithKline, Johnson & Johnson, Merck, Novartis, Ono Pharma, Otsuka, RJ Reynolds, Roche, Sankyo, Schering‐Plough, Scios, and Wyeth); advising regarding clinical trials (Altana, AstraZeneca, Aventis, Centocor, GlaxoSmithKline, Novartis, Pfizer, and Philip Morris); speaking at continuing medical education programmes; and performing funded research at both basic and clinical levels (Altana, AstraZeneca, Boehringer Ingelheim, GlaxoSmithKline, and Novartis). He owns no stock in any pharmaceutical companies. Drs Watsky and Reeves and Mr Anziano are employees of Pfizer and own Pfizer stock or have stock options."	
Notes	Bupropion was an active control for varenicline. Bupropion versus placebo and bupropion versus 2 mg varenicline comparisons contribute to review. Inclusion of 6 pretreatment dropouts has minimal effect on risk ratio	
***Risk of bias***		
**Bias**	**Authors' judgement**	**Support for judgement**
Random sequence generation (selection bias)	Low risk	Quote: "...a randomization list was computer generated using a method of randomly permuted blocks and a pseudorandom number generator."
Allocation concealment (selection bias)	Low risk	Quote: "Investigators assigned medication to subjects in numerical order of acceptance into the study."
Blinding (performance bias and detection bias) All outcomes	Unclear risk	Quote: "double‐blind", "to preserve treatment blinding," no further information provided
Incomplete outcome data (attrition bias) All outcomes	Low risk	Quote: "Subjects who dropped out for any reason were considered to be smokers at all subsequent time points." 9.5% of varenicline tartrate 0.3 mg, once daily; 7% of varenicline tartrate 1.0 mg, once daily; 11 % of varenicline tartrate 1.0 mg, twice daily; 6% of bupropion hydrochloride 150 mg, twice daily and 13% of the placebo group were lost to follow‐up.
Other bias	Low risk	None detected

**Table:** Parsons 2009

***Study characteristics***		
Methods	Study design: 2 x 2 factorial RCT Country: UK Setting: smoking cessation clinic Recruitment: direct mail from general practitioner (GP), stop smoking service, newspaper advertisements	
Participants	143 adult smokers; 62% female; average age 46; average cigarettes per day 21; mean FTND 5.5	
Interventions	St John's wort, 900 mg/day (300 mg x 3/day) for 14 weeks, started 2 weeks prior to TQD Placebo, same schedule as above Common components: 7 weekly individual behavioural support sessions in clinic	
Outcomes	Smoking cessation: prolonged abstinence at 6 months. Validated by CO ≤ 10 ppm Adverse events: serious adverse events at anytime within the study, and side effects in the first 4 weeks after quit day (2 weeks prior to quit day to 4 weeks afterward)	
Funding Source	Cancer Research UK	
Author conflicts of interest	None specified	
Notes	Factorial trial ‐ also tested the use of chromium versus placebo for weight loss. Arms combined for analyses; no difference detected	
***Risk of bias***		
**Bias**	**Authors' judgement**	**Support for judgement**
Random sequence generation (selection bias)	Low risk	Via computer program
Allocation concealment (selection bias)	Low risk	Independent statistician sent randomisation codes to medication packing company, medication allocated in sequence. Researchers blind to allocation
Blinding (performance bias and detection bias) All outcomes	Low risk	Quote: “Participants, therapists, and outcome assessors were blind to the treatment allocation.”
Incomplete outcome data (attrition bias) All outcomes	Low risk	Over 90% followed up at 6 months, similar between groups
Other bias	Low risk	None detected

**Table:** Perkins 2013

***Study characteristics***		
Methods	Study design: cross‐over trial Country: USA Setting: university research centre Recruitment method: "recruitment notices” were used	
Participants	45 participants randomised; 60% female; average age 36; average cigarettes per day 16; mean FTND 4.6	
Interventions	Bupropion, 150 mg once daily for 3 days, then 150 mg twice daily for 2 weeks Placebo, same schedule as above	
Outcomes	Smoking cessation (strictest definition): measures days abstinent per participant, which is not a relevant outcome to our review Adverse events: measured over three‐week treatment period	
Funding Source	Funded by National Institutes of Health	
Author conflicts of interest	"Dr Perkins has served as a consultant for Embera Neurotherapeutics, which is unrelated to the current study. Dr Lerman has served as a consultant for GlaxoSmithKline, Pfizer and Astra Zeneca. She has received research funding, unrelated to the current study, from Pfizer and Astra Zeneca. Dr Chengappa has research funding from Pfizer that is unrelated to the current study. Dr Sparks, Mr Karelitz and Ms Jao have no potential conflicts of interest or disclosures to report."	
Notes	Adverse events data not reported in a way that could be included in meta‐analysis	
***Risk of bias***		
**Bias**	**Authors' judgement**	**Support for judgement**
Random sequence generation (selection bias)	Unclear risk	Quote: “assigned randomly”, but no further information provided
Allocation concealment (selection bias)	Unclear risk	No relevant information given
Blinding (performance bias and detection bias) All outcomes	Low risk	Analysis of participants' knowledge of drug allocation revealed no significant differences between trial arms: “The respective number (percentage) of subjects identifying the medication as bupropion, modafinil, placebo or do not know were four, two, five and 34 (8.9, 4.4, 11.1 and 75.6%) of 45 during the bupropion condition; seven, three, four and 31 (15.6, 6.7, 8.9 and 68.9%) of 45 during the modafinil condition; and four, three, eight and 30 (9.1, 6.8, 18.2 and 67.9%) of 44 (1 subject with missing data) during the placebo condition. None of these values differed by medication condition, indicating successful blinding.”
Incomplete outcome data (attrition bias) All outcomes	Unclear risk	No relevant information given
Other bias	Low risk	None detected

**Table:** Piper 2007

***Study characteristics***		
Methods	Study design: RCT Setting: none specified Country: USA Recruitment: volunteers	
Participants	608 smokers; 58% female; average age 42; average cigarettes per day 22	
Interventions	Nicotine gum and bupropion. Gum at 4 mg. Bupropion at 300 mg Placebo gum and bupropion Double placebo Common components: three 10‐minute counselling sessions over 3 weeks	
Outcomes	Smoking cessation: ppa at 12 months. Validated by CO or blood cotinine Adverse events: measured for unspecified period	
Funding Source	National Institutes for Health	
Author conflicts of interest	In 1998 the University of Wisconsin appointed Dr Fiore to a named chair, made possible by an unrestricted gift to the university from GlaxoWellcome. Dr Baker has received monies to conduct clinical trials from pharmaceutical companies (Nabi, Glaxo, Pfizer, Sanofi).	
Notes		
***Risk of bias***		
**Bias**	**Authors' judgement**	**Support for judgement**
Random sequence generation (selection bias)	Unclear risk	"Randomization was conducted in double‐blind fashion using blocked randomization within each of the 10 [orientation session] cohorts." No further information provided
Allocation concealment (selection bias)	Unclear risk	Not stated
Blinding (performance bias and detection bias) All outcomes	Unclear risk	Described as double‐blind, but no further information
Incomplete outcome data (attrition bias) All outcomes	Low risk	32% of bupropion and 36% of placebo groups lost at 12 months. "Participants who could not be reached at follow‐up were considered to be smoking for the purposes of follow‐up analyses."
Other bias	Low risk	None detected

**Table:** Piper 2009

***Study characteristics***		
Methods	Study design: RCT Country: USA Setting: community Recruitment: volunteers	
Participants	1504 smokers; 58% female; average age 45; average cigarettes per day 21.4	
Interventions	Bupropion SR. 150 mg twice/day, 1 week pre‐quit, 8 weeks post‐quit Bupropion and nicotine lozenge. Duration and dosage as below Nicotine lozenge. 2 mg or 4 mg for 12 weeks (based on dose‐for‐dependence level as per instructions) Nicotine patch (24 hr, 21, 14, and 7 mg titrated down over 8‐week period post‐quit) Nicotine lozenge and nicotine patch. Duration and dosage as above Placebo bupropion Placebo bupropion and placebo lozenge Placebo lozenge Placebo patch Placebo lozenge and placebo patch Common components: 7 one‐to‐one 10‐ to 20‐minute counselling sessions	
Outcomes	Smoking cessation: 7 day ppa at 6 months. Validated by CO < 10 ppm Adverse events: measured for 10 weeks	
Funding Source	Majority of funding from National Institute on Drug Abuse and National Center for Research Resources. Medication provided to participants at no extra cost by GlaxoSmithKline.	
Author conflicts of interest	"The authors report the following potential conflicts of interest for the last 5 years: Dr Smith has received research support from Elan Corporation. Dr Baker has served as an investigator on research projects sponsored by pharmaceutical companies, including Sanofi‐Synthelabo, Pfizer Inc, and Nabi Biopharmaceuticals. Dr Jorenby has received research support from the National Institute on Drug Abuse, the National Cancer Institute, Pfizer Inc, Sanofi‐Synthelabo, and Nabi Biopharmaceuticals. He has received support for educational activities from the National Institute on Drug Abuse and the Veterans Administration and consulting fees from Nabi Biopharmaceuticals. Dr Fiore has received honoraria from Pfizer. He has served as an investigator on research studies at the University of Wisconsin that were funded by Pfizer, Sanofi‐Synthelabo, GlaxoSmithKlein, and Nabi Biopharmaceuticals. In 1998, the University of Wisconsin appointed Dr Fiore to a named chair funded by an unrestricted gift to University of Wisconsin from Glaxo Wellcome."	
Notes	Placebo outcomes reported as a whole in published report; author provided data for individual groups. 1 versus 6 in Analyses 1.1, 1.2 and 1.3. 2 versus 3 included in Analysis 1.5. 1 versus 4 in Analysis 1.7.1, 1 versus 3 in Analysis 1.7.2 and 1 versus 5 in Analysis 1.7.3 (intervention arm split in three to avoid triple counting)	
***Risk of bias***		
**Bias**	**Authors' judgement**	**Support for judgement**
Random sequence generation (selection bias)	Unclear risk	Method of sequence generation not specified. "Randomization was double‐blind and used a block randomization scheme with sex and self‐reported race as the blocking variables."
Allocation concealment (selection bias)	Low risk	"Staff did not know to which type(s) of medication a participant would be assigned until the moment of randomization, and study staff were blinded to whether the medication was active or placebo."
Blinding (performance bias and detection bias) All outcomes	Unclear risk	"Double blind" but no further detail provided. "Study staff were blinded to whether the medication was active or placebo" Comment: type of medication (i.e. patch, gum, pill) would have been apparent to both groups
Incomplete outcome data (attrition bias) All outcomes	Low risk	90 dropouts (out of 1504). Analyses conducted using ITT. Individuals with missing data considered to be smoking
Other bias	Low risk	None detected

**Table:** Planer 2011

***Study characteristics***		
Methods	Study design: RCT Country: Israel Setting: hospitals, Jersulem Recruitment: patients hospitalised for acute coronary syndrome in 2 separate campuses in Jerusalem	
Participants	151 smokers with diagnosis of acute coronary syndrome, motivated to quit; average age 51.9; 20.1% female; average cigarettes per day 31	
Interventions	Bupropion, 150 mg once daily for 3 days, then twice daily for 2 months Placebo, same schedule as above Common components: counselling (at least 15 minutes of motivational support) during hospitalisation and continued after discharge (at least 2 visits with physician and nurse at 1 month and 2 months and weekly telephone call by nurse during first and second month, then monthly telephone calls during rest of the year)	
Outcomes	Smoking cessation: self‐reported continuous abstinence at 12 months Adverse events: measured for 12 months	
Funding Source	GlaxoSmithKline	
Author conflicts of interest	None specified	
Notes	Study stopped early after interim analysis indicated no benefit Odds ratio adjusted for age, sex, invasive procedure, risk factors, Fagerstrom score, cigarettes per day: 0.90 (95% confidence interval (CI) 0.39 to 2.09)	
***Risk of bias***		
**Bias**	**Authors' judgement**	**Support for judgement**
Random sequence generation (selection bias)	Unclear risk	Quote: "Randomized"; method not specified
Allocation concealment (selection bias)	Unclear risk	Method not specified
Blinding (performance bias and detection bias) All outcomes	Low risk	Participants and staff blind to treatment assignment. Quote: "Numbered study bottles were supplied by the study co‐ordinator and remained concealed from the patients and medical staff." Comment: no biochemical validation but participants blind to condition so differential misreport unlikely.
Incomplete outcome data (attrition bias) All outcomes	Low risk	1 lost to follow‐up in each group
Other bias	Low risk	None detected

**Table:** Prochazka 1998

***Study characteristics***		
Methods	Study design: RCT Country: USA Setting: VAMC and Army Medical Centre Recruitment: outpatient clinics and campus advertisements	
Participants	214 smokers (excludes 29 early dropouts); 38% female; average age 47	
Interventions	Nortriptyline, maximum 75 mg/day from 10 days pre‐quit date to 8 weeks after, tapered for 2 weeks Placebo capsules Common components: 2 behavioural group sessions prior to drug therapy. During treatment, individual support was provided by the study nurse.	
Outcomes	Smoking cessation: prolonged abstinence at 6 months. Validated by CO ≤ 9 ppm at each visit and urine cotinine < 50 ng/mL at 6 months Adverse events: measured for unspecified period	
Funding Source	Department of Veterans Affairs, US Department of Defense	
Author conflicts of interest	None specified	
Notes		
***Risk of bias***		
**Bias**	**Authors' judgement**	**Support for judgement**
Random sequence generation (selection bias)	Unclear risk	Randomisation method not described
Allocation concealment (selection bias)	Unclear risk	Not described
Blinding (performance bias and detection bias) All outcomes	High risk	Quote: "An unblinded research pharmacist recommended dosage reductions for those above the therapeutic range and dosage increases for those who were subtherapeutic. To maintain blinding, dose reductions and increases on an equal number of randomly selected placebo‐treated subjects were also recommended...our blinding was only partially effective. Because of the high frequency of dry mouth, the nurse and subjects were often able to identify the active drug."
Incomplete outcome data (attrition bias) All outcomes	High risk	75% dropout rate in placebo, 61% in drug group, majority classified as ineffective therapy
Other bias	Low risk	None detected

**Table:** Prochazka 2004

***Study characteristics***		
Methods	Study design: RCT Country: USA Setting: clinic Recruitment: outpatient clinic and community volunteers	
Participants	158 smokers; 54% female; average cigarettes per day 22	
Interventions	Nortriptyline and nicotine patch, maximum 75 mg/day for 14 weeks, from 2 weeks before TQD tapered for 2 weeks. Nicotine patch 8 weeks from TQD Placebo capsules and nicotine patch Common components: brief counselling from nurse at weekly visits	
Outcomes	Smoking cessation: prolonged abstinence at 6 months. Validated by CO ≤ 9 ppm at each visit, cotinine < 50 ng/mL at 6 months Adverse events: measured for unspecified period	
Funding Source	Department of Veterans Affairs	
Author conflicts of interest	None specified	
Notes		
***Risk of bias***		
**Bias**	**Authors' judgement**	**Support for judgement**
Random sequence generation (selection bias)	Low risk	Quote: "Subjects were stratified by history of previous major depression and randomized by means of a computer‐generated random number list that was held by the Research Pharmacy Service of the Denver Veterans Affairs Medical Center."
Allocation concealment (selection bias)	Low risk	Quote: "Once a patient was enrolled, the Research Pharmacy Service randomized the subject according to the randomization list." Judged adequate
Blinding (performance bias and detection bias) All outcomes	High risk	Quote: "...our blinding was only partially effective. Because of the high frequency of dry mouth, the study nurse was often able to identify the active drug."
Incomplete outcome data (attrition bias) All outcomes	Low risk	Quote: "Subjects who dropped out were counted as smokers." Number of dropouts not given
Other bias	Low risk	None detected

**Table:** Qin 2021

***Study characteristics***		
Methods	Study design: RCT Country: China Setting: China–Japan Friendship hospital in Beijing Recruitment method: via a trial site, a hotline of smoking cessation, advertisements in the community from February 2019 to June 2020	
Participants	136 smokers diagnosed with COPD randomised; 2.9% female, average age 62; average cigarettes per day 19.21; FTND mode 0‐3	
Interventions	Varenicline, 2 mg/day for 12 weeks Bupropion, 150 mg/day for 12 weeks Common components: "Participants received a counseling session for more than 60 minutes when they began medication at week 0, and they also received up to 10 min of counseling at weeks 1, 2, 4, 6, 9, 12, and 24."	
Outcomes	Smoking cessation: abstinence at 24 weeks (no further details given). Validated by CO ≤ 10 ppm	
Funding Source	Quote: "This study was supported by the Capital Health Development Research Project in China (Grant No. 2018‐2‐4066), the National Natural Science Foundation of China (Grant No. 81720108001) and the National Key R&D Program of China (Grant No. 2017YFC1309400)."	
Author conflicts of interest	Quote: "The authors report no conflicts of interest in this work."	
Notes		
***Risk of bias***		
**Bias**	**Authors' judgement**	**Support for judgement**
Random sequence generation (selection bias)	Low risk	Quote: "A biostatistician, inde‐ pendent of the study used Proc Plan in SAS version 9.4 (SAS Institute) to generate a table of random digit to randomly assign the numbers to the two groups. (the number of the random seed is 87,654,321)."
Allocation concealment (selection bias)	Low risk	Quote: "To ensure random concealment, the group information assigned to each participant was put in a sealed, and opaque envelope."
Blinding (performance bias and detection bias) All outcomes	Low risk	Quote: ""Because of the different medication packaging, only statisticians were blinded to medication allocation" however, all participants received an active evidence‐based smoking cessation pharmacotherapy."
Incomplete outcome data (attrition bias) All outcomes	Low risk	Quote: Only 8/68 and 7/68 lost in the varenicline and bupropion arms respectively."
Other bias	Low risk	None detected

**Table:** Richmond 2013

***Study characteristics***		
Methods	Study design: RCT Country: Australia Setting: 18 prisons Recruitment: referral from clinic staff, flyers and posters in prisons	
Participants	425 male prisoners aged > 18, incarcerated for ≥ 1 month with ≥ 6 months of current sentence remaining; FTND ≥ 5; average age 34; average cigarettes per day 23; 83% FTND ≥ 6	
Interventions	Nortriptyline, tablet form for 13 weeks (TQD week 3. Week 1: 25 mg/day for 3 days, 50 mg/day for 4 days. Weeks 2 to 12 75 mg/day. Week 13 50 mg/day for 4 days, then 25 mg/day for 3 days) Placebo, same schedule as above Common components: two x 30‐minute counselling sessions with CBT. Self‐help materials, access to quit‐line. 10 weeks NRT patch started on TQD; 21 mg weeks 1 to 6, 14 mg/day weeks 7 to 8, 7 mg/day weeks 9 to 10	
Outcomes	Smoking cessation: continuous abstinence at 12 months. Validated by CO < 10 ppm Adverse events: measured for unspecified period	
Funding Source	National Health and Medical Research Council, NSW Department of Health, Queensland Department of Health. NRT provided free of charge by GlaxoSmithKline.	
Author conflicts of interest	Tony Butler is supported by an ARC future Fellowship	
Notes	N quit extrapolated from percentages provided	
***Risk of bias***		
**Bias**	**Authors' judgement**	**Support for judgement**
Random sequence generation (selection bias)	Unclear risk	Quote: “Randomization algorithm,” no further information provided
Allocation concealment (selection bias)	Unclear risk	Not specified
Blinding (performance bias and detection bias) All outcomes	Unclear risk	Quote: “Follow‐up assessments were conducted… by a prison nurse research assistant who was blind to group allocation.” Identical placebo. No further information on blinding provided
Incomplete outcome data (attrition bias) All outcomes	Low risk	80% followed up at 12 months, similar in both groups
Other bias	Low risk	None detected

**Table:** Rigotti 2006

***Study characteristics***		
Methods	Study design: RCT Country: USA Setting: hospitals Recruitment: volunteers	
Participants	248 smokers hospitalised with cardiovascular disease (excludes 3/3 dropped prior to treatment and 2 placebo deaths during follow‐up); 31% female; average age 56; average cigarettes per day 23/21	
Interventions	Bupropion 300 mg for 12 weeks Placebo, same schedule as above Common components: multicomponent CBT cessation and relapse prevention programme, motivational interviewing approach. Begun in hospital, 30 to 45 minutes; 5 x 10‐minute post‐discharge contacts (at 2 days, 1 week, 3 weeks, 8 weeks, 12 weeks), self‐help, chart prompt for physician. Total time 80 to 95 minutes	
Outcomes	Smoking cessation: sustained abstinence at 12 months (at multiple follow‐ups). Validated by saliva cotinine at 12 weeks and 52 weeks, CO at 2 weeks and 4 weeks Adverse events: measured for 52 weeks	
Funding Source	National Heart, Lung and Blood Institute, National Institutes of Health General Clinical Research Centers Program, GlaxoSmithKline	
Author conflicts of interest	None specified	
Notes		
***Risk of bias***		
**Bias**	**Authors' judgement**	**Support for judgement**
Random sequence generation (selection bias)	Low risk	Quote: "Using a computer program, the study statistician generated a sequence of randomly‐permuted blocks of 4 within strata formed by study site and daily cigarette consumption (10 vs 10)."
Allocation concealment (selection bias)	Low risk	Quote: "The study pharmacist used this sequence, concealed from enrolment staff, to assign participants to study arm. Subjects and study personnel, except the statistician and pharmacist, were blind to treatment assignment."
Blinding (performance bias and detection bias) All outcomes	Low risk	Quote: "Subjects and study personnel, except the statistician and pharmacist, were blind to treatment assignment."
Incomplete outcome data (attrition bias) All outcomes	Low risk	Quote: "Subjects were considered smokers if they were lost to follow‐up..."; 23% lost to follow‐up in the bupropion group and 23% in the placebo group
Other bias	Low risk	None detected

**Table:** Rose 2013

***Study characteristics***		
Methods	Study design: RCT Country: USA Setting: clinic Recruitment: community volunteers	
Participants	440 smokers who did not respond successfully to cessation treatment with NRT (phase 1 = 335 participants whose smoking did not decrease by > 50% after 1 week NRT (prior to TQD); phase 2 = 105 participants who lapsed within one week after TQD); 50% female; average age 43; average cigarettes per day 22; mean FTND 5.8	
Interventions	Bupropion and nicotine patch. Bupropion for 12 weeks (150 mg/day for 3 days, 300 mg/d for remainder). Nicotine patch (patch dose based on CO, 21 mg/day for CO ≤ 30 ppm, 42 mg/day for CO > 30 ppm) Placebo and nicotine patch. Dosing as above Common components: cessation programme with nicotine patch (discontinued after 1 week in Phase 1 varenicline arm) and 4 to 6 brief (< 15 minutes) counselling sessions	
Outcomes	Smoking cessation: continuous abstinence at 6 months. Validated by CO ≤ 10 ppm Adverse events: measured for 6 weeks after the quit date	
Funding Source	Supported by grant to Duke University from Philip Morris USA. NRT donated by GlaxoSmithKline	
Author conflicts of interest	Dr Rose has served as a consultant for Targacept and Philip Morris USA and has a patent purchase agreement with Philip Morris International. Both authors have received research funding from Philip Morris USA.	
Notes	Phase 1 and Phase 2 combined in meta‐analysis. Sensitivity analyses including both separately did not detect any significant effect on the pooled result.	
***Risk of bias***		
**Bias**	**Authors' judgement**	**Support for judgement**
Random sequence generation (selection bias)	Unclear risk	Not reported
Allocation concealment (selection bias)	Unclear risk	Not reported
Blinding (performance bias and detection bias) All outcomes	Unclear risk	Quote: "Double‐blind"; no further information provided
Incomplete outcome data (attrition bias) All outcomes	High risk	< 50% followed up at 6 months in both phases, similar rates of dropout across all arms. 27 participants censored from reported analyses, mainly for protocol violations, included as smoking here.
Other bias	Low risk	None detected

**Table:** Rose 2014

***Study characteristics***		
Methods	Study design: RCT Country: USA Setting: university Recruitment method: newspaper, radio, and television advertisements	
Participants	Participants were nicotine patch non‐responders (failed to show a reduction of more than 50% in smoking after 1 week of nicotine patch treatment) 222 participants randomised; 54.5% female; average age 44.1; average cigarettes per day 20.7; mean FTND 6.1	
Interventions	Bupropion and varenicline. Bupropion given 150 mg once daily for 3 days, then 150 mg twice daily for remainder of 12‐week treatment period. Varenicline given 0.5 mg once daily on days 1 to 3, 0.5 mg twice daily on days 4 to 7; and 1 mg twice daily for remainder of 12‐week treatment period Placebo and varenicline. Given according to schedule above Common components: brief support at each study session, totalling 1 hour and 45 minutes	
Outcomes	Smoking cessation (strictest definition): 7‐day ppa at 6 months. Validated by CO ≤ 10 ppm Adverse events: measured for an unspecified period	
Funding Source	National Institute on Drug Abuse grant 1P50 DA027840 and a grant from Philip Morris USA. The sponsors had no role in the planning or execution of the study, data analysis, or publication of results. Active bupropion sustained‐release and placebo tablets were supplied by Murty Pharmaceuticals, under contract from the National Institute on Drug Abuse.	
Author conflicts of interest	The authors have consulting and patent purchase agreements with Philip Morris International for nicotine inhalation technology and consulting agreements with Targacept and Novartis.	
Notes		
***Risk of bias***		
**Bias**	**Authors' judgement**	**Support for judgement**
Random sequence generation (selection bias)	Unclear risk	No relevant information given
Allocation concealment (selection bias)	Unclear risk	No relevant information given
Blinding (performance bias and detection bias) All outcomes	Unclear risk	Quote: “The study was a double‐blind, parallel‐arm adaptive treatment trial." Placebo tablets were used. No further information provided regarding who was blinded
Incomplete outcome data (attrition bias) All outcomes	Low risk	Dropout rates are as follows: 41/113 (36.3%) for varenicline and bupropion; 38/109 (34.9%) for varenicline and placebo
Other bias	Low risk	None detected

**Table:** Rose 2017

***Study characteristics***		
Methods	Study design: RCT Country: USA Setting: research centre (Duke Center for Smoking Cessation) Recruitment method: not specified	
Participants	All participants were male 174 participants randomised; 0% female; average age 44.0; average cigarettes per day 20.0; mean FTND 5.5	
Interventions	Bupropion and varenicline. Bupropion scheduling was 150 mg once daily for 3 days, followed by 150 mg twice daily for the remainder of the 12‐week treatment period. Varenicline scheduling was 0.5 mg once daily on days 1 to 3, 0.5 mg twice daily on days 4 to 7, followed by 1 mg twice daily for the remainder of the 12‐week treatment period Placebo and varenicline. Same schedule as above Common components: pre‐cessation patches for 1 week prior to pharmacological treatments above, and brief support was provided at each session, totalling 1 hour and 30 minutes	
Outcomes	Smoking cessation (strictest definition): 11 weeks ‐ too short a follow‐up for this outcome to be considered in this review Adverse events: measured for 12 weeks	
Funding Source	Grant 1P50 DA027840 from the National Institute on Drug Abuse and a grant from Philip Morris, USA	
Author conflicts of interest	The authors disclose consulting and patent purchase agreements with Philip Morris International relating to reduced risk tobacco products.	
Notes		
***Risk of bias***		
**Bias**	**Authors' judgement**	**Support for judgement**
Random sequence generation (selection bias)	Unclear risk	No relevant information given
Allocation concealment (selection bias)	Unclear risk	No relevant information given
Blinding (performance bias and detection bias) All outcomes	Unclear risk	Quote: “double‐blind, placebo‐controlled, parallel‐arm trial”. No further information provided regarding who was blinded
Incomplete outcome data (attrition bias) All outcomes	Low risk	Dropout rates are as follows: (13.1%) in the bupropion and varenicline arm; 13/90 (14.4%) in the placebo and varenicline arm; 11/84. Therefore dropout was low and similar between groups.
Other bias	Low risk	None detected

**Table:** Rovina 2003

***Study characteristics***		
Methods	Study design: RCT Country: Greece Setting: cessation clinic Recruitment: not reported	
Participants	233 heavy smokers randomised; 46% female, average age 46; average packs of cigarettes per year 48.3	
Interventions	Short‐term treatment with bupropion HCl, 300 mg/day for 7 weeks Prolonged treatment with bupropion HCl, 300 mg/day for 19 weeks	
Outcomes	Continuous abstinence at 26 weeks	
Funding Source	Supported by Thorax Foundation	
Author conflicts of interest	Not reported	
Notes	Only abstract available	
***Risk of bias***		
**Bias**	**Authors' judgement**	**Support for judgement**
Random sequence generation (selection bias)	Unclear risk	No information available
Allocation concealment (selection bias)	Unclear risk	No information available
Blinding (performance bias and detection bias) All outcomes	High risk	Unblinded with differing lengths of pharmacotherapy treatment between arms.
Incomplete outcome data (attrition bias) All outcomes	High risk	Quote: "The program was successfully completed in 60% of the participants of group A and in 35% of the participants of group B. 9% discontinued because of side effects of bupropion HCl. The drop out rate was similar in between the two groups for the first 7 weeks. An additional 20% of the participants discontinued the study during the prolonged treatment."
Other bias	Low risk	None detected

**Table:** Rovina 2009

***Study characteristics***		
Methods	Study design: RCT Country: Greece Setting: cessation clinic Recruitment: clinic attenders invited to participate	
Participants	205 smokers; 40% female; average age 45; average cigarettes per day 37	
Interventions	Bupropion 300 mg/day for 19 weeks plus 15 minutes of physician counselling Bupropion 300 mg/day for 19 weeks plus nonspecific group therapy, 1 hour weekly for 1 month, then every 3 weeks until 19 weeks Bupropion 300 mg/day for 19 weeks plus CBGT, same schedule CBGT without bupropion	
Outcomes	Smoking cessation: continuous abstinence at 12 months after end of treatment. Validated by CO ≤ 10 ppm Adverse events: measured for 31 weeks	
Funding Source	None specified	
Author conflicts of interest	"All the authors of this paper declare that they have no financial or other potential conflicts of interest concerning the subject of this manuscript."	
Notes	3 versus 4 used in analyses; 1 and 2 not included in any analyses (effect of different counselling would confound effect of bupropion) Authors do not report N abstinent; numbers included in meta‐analysis extrapolated from applying percentage to overall N randomised	
***Risk of bias***		
**Bias**	**Authors' judgement**	**Support for judgement**
Random sequence generation (selection bias)	Unclear risk	Randomised, method not stated, 3:1:1:1 ratio
Allocation concealment (selection bias)	Unclear risk	No details reported
Blinding (performance bias and detection bias) All outcomes	High risk	Open‐label, participants and staff aware of allocation
Incomplete outcome data (attrition bias) All outcomes	Unclear risk	90% followed up at 12 months, but authors do not specify percentage per group and do not specify how participants lost to follow‐up were treated. Authors only provide percentages abstinent, so number abstinent in this review may be inflated.
Other bias	Low risk	None detected

**Table:** Saules 2004

***Study characteristics***		
Methods	Study design: RCT Country: USA Setting: cessation clinic Recruitment: volunteers	
Participants	150 smokers; 55% female; average age 40	
Interventions	Fluoxetine 40 mg for 14 weeks, nicotine patch for 10 weeks Fluoxetine 20 mg for 14 weeks, nicotine patch for 10 weeks Placebo and nicotine patch Common components: TQD end of week 4, CBT 6 sessions starting 2 weeks before TQD, 11 clinic visits	
Outcomes	Smoking cessation: at 12 months (unspecified definition). Validated by CO < 10 ppm Adverse events: measured for 15 weeks	
Funding Source	National Institute on Drug Abuse, State of Michigan. Nicotine patch provided by McNeil Consumer Healthcare	
Author conflicts of interest	None specified	
Notes	Authors provided quit numbers by treatment group	
***Risk of bias***		
**Bias**	**Authors' judgement**	**Support for judgement**
Random sequence generation (selection bias)	Unclear risk	Randomisation method not described
Allocation concealment (selection bias)	Unclear risk	Not described
Blinding (performance bias and detection bias) All outcomes	Unclear risk	Quote: "Double‐blind" but no further information provided
Incomplete outcome data (attrition bias) All outcomes	Unclear risk	Numbers lost to follow‐up not provided by study arm but high: at six months, only 58 of 150 participants were followed up. Participants who dropped out of the study or were lost to follow‐up were considered to be smoking again.
Other bias	Low risk	None detected

**Table:** Schepis 2006

***Study characteristics***		
Methods	Study design: RCT Country: no information available Setting: no information available Recruitment method: no information available	
Participants	Preliminary data in abstract states 15 smokers (aged 14 to 25) randomised; no further information available	
Interventions	Placebo plus Modified Brief Office Intervention (M‐BOI) for 9 weeks Bupropion (300 mg/day) plus Modified Brief Office Intervention (M‐BOI). Bupropion 300 mg/day for 9 weeks Common components: the interactive behavioural support, M‐BOI, was a novel cognitive‐behavioral treatment and common between groups	
Outcomes	Smoking cessation: definition not reported	
Funding Source	No information available	
Author conflicts of interest	No information available	
Notes	Only abstract with preliminary short‐term data available. Study does not contribute to any data analyses.	
***Risk of bias***		
**Bias**	**Authors' judgement**	**Support for judgement**
Random sequence generation (selection bias)	Unclear risk	No information available
Allocation concealment (selection bias)	Unclear risk	No information available
Blinding (performance bias and detection bias) All outcomes	Low risk	Placebo‐controlled and contact matched between arms
Incomplete outcome data (attrition bias) All outcomes	Unclear risk	No information available
Other bias	Low risk	None detected

**Table:** Schmitz 2007

***Study characteristics***		
Methods	Study design: 2 x 2 factorial RCT Country: USA Setting: research clinic Recruitment: community volunteers	
Participants	154 women smokers; average age 48; average cigarettes per day 21	
Interventions	Bupropion 300 mg/day for 7 weeks Placebo Common components: either CBT based on relapse prevention model, or group support therapy, both 7 weekly 60‐minute meetings, TQD morning of 1st session, 10 days after start of medications	
Outcomes	Smoking cessation: 7 day ppa at 12 months. Validated by CO ≤ 10 ppm, saliva cotinine < 15 ng/mL Adverse events: 7 weeks	
Funding Source	National Institute on Drug Abuse. Bupropion provided by GlaxoSmithKline	
Author conflicts of interest	None specified	
Notes	Group therapy variants combined in main analysis	
***Risk of bias***		
**Bias**	**Authors' judgement**	**Support for judgement**
Random sequence generation (selection bias)	Low risk	Urn procedure, balancing on a range of outcome‐related variables
Allocation concealment (selection bias)	Low risk	Quote: "Investigators and research staff were blind to the randomization codes, which were kept by a faculty member independent of the research and treatment team."
Blinding (performance bias and detection bias) All outcomes	Unclear risk	Quote: "Double‐blind"; further information not provided
Incomplete outcome data (attrition bias) All outcomes	Low risk	14 'enrolment failures' who did not receive any treatment were excluded from analyses. Other non‐completers and losses to follow‐up included in ITT analysis
Other bias	Low risk	None detected

**Table:** Schnoll 2010

***Study characteristics***		
Methods	Study design: RCT Country: USA Setting: not specified (presumably clinic) Recruitment: patient lists from physicians treating people with cancer	
Participants	246 cancer patients smoking ≥ 2 cigarettes per day; 48% female; average age 54.8; average cigarettes per day 17.5; mean FTND 3.2; 32% had tobacco‐related tumours	
Interventions	Bupropion 9 weeks, started 2 weeks before TQD (150 mg/day first week, 300 mg/day remaining 8 weeks) Placebo, same schedule as above Common components: 8 weeks nicotine patches and 5 sessions of behavioural counselling (3 in person, 2 over phone)	
Outcomes	Smoking cessation: 7‐day ppa at 6 months. Validated by CO ≤ 10 ppm Adverse events: measured for 9‐week treatment period	
Funding Source	National Cancer Institute. NRT provided free of charge from GlaxoSmithKline.	
Author conflicts of interest	None specified	
Notes		
***Risk of bias***		
**Bias**	**Authors' judgement**	**Support for judgement**
Random sequence generation (selection bias)	Unclear risk	Stratified by depression status. Method of sequence generation not specified
Allocation concealment (selection bias)	Unclear risk	Not specified
Blinding (performance bias and detection bias) All outcomes	Unclear risk	Quote: "Double‐blind," no further information provided
Incomplete outcome data (attrition bias) All outcomes	Low risk	65% intervention and 72% control followed up at 6 months
Other bias	Low risk	None detected

**Table:** Selby 2003

***Study characteristics***		
Methods	Study design: RCT Country: Canada Setting: 15 clinical centres Recruitment: community volunteers	
Participants	284 smokers previously exposed to bupropion for at least 2 weeks; had not quit for more than 24 hours in previous month	
Interventions	Bupropion 300 mg for 12 weeks Placebo Behavioural support not described	
Outcomes	Smoking abstinence, ppa at 12 months. Validated by CO ≤ 10 ppm at treatment visits Adverse events: measured for unspecified period	
Funding Source	None specified	
Author conflicts of interest	None specified	
Notes	Based on abstract	
***Risk of bias***		
**Bias**	**Authors' judgement**	**Support for judgement**
Random sequence generation (selection bias)	Unclear risk	Randomisation method not described
Allocation concealment (selection bias)	Unclear risk	No details given
Blinding (performance bias and detection bias) All outcomes	Unclear risk	No details given
Incomplete outcome data (attrition bias) All outcomes	Unclear risk	No details given; unclear how participants lost to follow‐up treated in outcome data. 70% intervention group and 50% control group completed study
Other bias	Low risk	None detected

**Table:** Sheng 2013

***Study characteristics***		
Methods	Study design: RCT Country: China Setting: hospital outpatient centres Recruitment method: newspaper advertisements and by word of mouth	
Participants	Participants were mainly male 257 participants randomised; 5.5% female; average age 39.1; average cigarettes per day 22.5; mean FTND 5.6	
Interventions	Bupropion 150 mg daily for days 1 to 3, 150 mg twice daily for days 4 to 56, then 150 mg daily for days 57 to 63 and discontinued on day 64 Placebo: same tablets and schedule as for bupropion above All participants were given the same brief education and counselling was administered to both groups by research staff. Counselling topics included motivation, identification of smoking triggers, coping responses, weight management, and use of the medications. The total duration of counselling was 1 hour and 30 minutes.	
Outcomes	Smoking cessation: 12 weeks ‐ too short a follow‐up for this outcome to be considered in this review Adverse events: not specified	
Funding Source	Zhejiang Jinxin Pharmaceutical Co, Ltd	
Author conflicts of interest	L‐XS, Z‐NJ, and G‐ZX declare that they have undertaken research and consultancy for, and received honoraria for speaking at meetings for, the manufacturers of smoking cessation medications.	
Notes		
***Risk of bias***		
**Bias**	**Authors' judgement**	**Support for judgement**
Random sequence generation (selection bias)	Low risk	Quote: “Participants were assigned to one of two study arms using a computer algorithm to generate a random list of treatment assignments."
Allocation concealment (selection bias)	Unclear risk	No relevant information given
Blinding (performance bias and detection bias) All outcomes	Low risk	Quote: “Participants in the control arm received placebo pills identical in appearance. All study personnel were blinded to treatment assignment. The same brief education and counseling were administered to both groups by a research staff.”
Incomplete outcome data (attrition bias) All outcomes	Low risk	Dropout rates are as follows: 14/127 (11.0%) in the bupropion arm; 18/130 (13.9%) in the placebo. Therefore, dropout rates are low and similar between groups.
Other bias	Low risk	None detected

**Table:** Siddiqi 2013

***Study characteristics***		
Methods	Study design: cluster‐RCT Country: Pakistan Setting: 33 health centres Recruitment: patients from participating health centres with suspected pulmonary tuberculosis	
Participants	1955 adult smokers with suspected tuberculosis (1299 included in arms relevant to this review), smoking ≥ 1 cigarettes per day or smoking hookah on a daily basis; 5% female; average age 41; average cigarettes per day 19 (where one hookah counts as 2 cigarettes)	
Interventions	Bupropion 7 weeks (75 mg/day first week, 150 mg/day thereafter) No pharmacotherapy Common components: 2 sessions of brief, in‐person behavioural support (Note: third arm received usual care only; not included in this review)	
Outcomes	Smoking cessation: continuous abstinence at 6 months. Validated by CO ≤ 9 ppm	
Funding Source	International Development Research Centre	
Author conflicts of interest	Link provided to list of declarations of interest, but link does not give access to active webpage	
Notes	Reported narratively only due to substantial heterogeneity of programme effects across clusters. 275/659 quit intervention versus 254/640 control, adjusted risk ratio 1.1 (0.5 to 2.3)	
***Risk of bias***		
**Bias**	**Authors' judgement**	**Support for judgement**
Random sequence generation (selection bias)	Low risk	Computer‐generated
Allocation concealment (selection bias)	Low risk	Quote: “A researcher who was blinded to center identity” allocated conditions
Blinding (performance bias and detection bias) All outcomes	High risk	No blinding
Incomplete outcome data (attrition bias) All outcomes	Low risk	No clinics dropped out post‐randomisation. Over 90% of participants followed up at 6 months
Other bias	High risk	Substantial heterogeneity of programme effects across clusters. 20% of participants in control arm smoked only hookah (no cigarettes) compared to 4% in intervention arm

**Table:** Simon 2004

***Study characteristics***		
Methods	Study design: RCT Country: USA Setting: VAMC outpatient units Recruitment: outpatients	
Participants	244 smokers, 79% veterans; 5% female; average age 50; average cigarettes per day 24	
Interventions	Bupropion and nicotine patch. Bupropion at 300 mg for 7 weeks. Nicotine patch for 2 months Placebo bupropion and nicotine patch. Schedules as above Common components: 3 months CBT counselling, self‐help materials, and telephone follow‐up counselling	
Outcomes	Smoking cessation: sustained abstinence at 12 months (sustained at multiple follow‐ups). Validated by saliva cotinine Adverse events: measured for 8 weeks	
Funding Source	California Tobacco‐Related Disease Research Program	
Author conflicts of interest	None specified	
Notes	Used in bupropion plus NRT versus NRT comparison 2 placebo and 3 bupropion deaths excluded from denominators Originally based on abstract, now uses published data and sustained quitting outcome	
***Risk of bias***		
**Bias**	**Authors' judgement**	**Support for judgement**
Random sequence generation (selection bias)	Low risk	Quote: "We assigned participants to the 2 study arms by using a computer algorithm to generate a random list of treatment assignments."
Allocation concealment (selection bias)	Unclear risk	Not described
Blinding (performance bias and detection bias) All outcomes	Unclear risk	Quote: "All study personnel engaged in providing interventions to participants were blinded to treatment assignment." "Blinding appeared to be effective in our study; an approximately equal number of participants were able to guess what their treatment had been at the end of the study."
Incomplete outcome data (attrition bias) All outcomes	Low risk	Quote: "Of the 244 participants enrolled, 3 (1%) were lost to follow‐up (all randomized to the placebo arm).... Participants lost to follow‐up were considered smokers."
Other bias	Low risk	None detected

**Table:** Simon 2009

***Study characteristics***		
Methods	Study design: RCT Country: USA Setting: VAMC hospital Recruitment: hospitalised volunteers	
Participants	83 inpatients smoking at least 5 cigarettes per day in previous year, smoking in week before admission, in contemplation or preparation stage of change	
Interventions	Bupropion 300 mg for 7 weeks Placebo Common components: individual CBT 30 to 60 minutes during hospital stay plus 5 phone calls at week 1, week 3, week 5, week 8, week 1. Participants who had returned to smoking were encouraged to make another quit attempt.	
Outcomes	Smoking cessation: continuous abstinence at 6 months. Validated at each visit by saliva cotinine < 15 ng/mL Adverse events: measured for 7 weeks	
Funding Source	California Tobacco‐Related Disease Research Program	
Author conflicts of interest	None specified	
Notes	1 death in bupropion, 1 in placebo excluded from analyses	
***Risk of bias***		
**Bias**	**Authors' judgement**	**Support for judgement**
Random sequence generation (selection bias)	Low risk	Quote: "computer algorithm to generate a random list of treatment assignments."
Allocation concealment (selection bias)	Unclear risk	Not described
Blinding (performance bias and detection bias) All outcomes	Low risk	Quote: "All study personnel engaged in providing interventions to participants were blinded to treatment assignment." "A significant percentage of participants were able to guess correctly whether they were taking active bupropion or placebo" but as results did not favour intervention group, authors suggest this unblinding did not bias the results.
Incomplete outcome data (attrition bias) All outcomes	Low risk	5 withdrawals, 1 lost to follow‐up, 1 death in placebo; 2 withdrawals, 1 lost to follow‐up, 1 death in bupropion. All except deaths included in meta‐analysis
Other bias	Low risk	None detected

**Table:** Singh 2010

***Study characteristics***		
Methods	Study design: RCT Country: India Setting: anti‐smoking clinic of Vallabhbhai Patel Chest Institute Recruitment method: not clearly specified	
Participants	Participants almost solely men 30 participants randomised; 3.3% female; average age 43.1; average cigarettes per day 18.8; mean FTND 5.6	
Interventions	Bupropion 300 mg daily for seven weeks Placebo Common components: physician advice based on National Cancer Institute's '5 As' approach, i.e. ASK, ADVICE, ASSESS, ASSIST and ARRANGE. Brief face‐to‐face personalised anti‐smoking advice was given at each of the 11 visits.	
Outcomes	Smoking cessation: 16 weeks ‐ too short a follow‐up for this outcome to be considered in this review Adverse events: measured for six weeks	
Funding Source	Quote: “nil”	
Author conflicts of interest	None declared	
Notes		
***Risk of bias***		
**Bias**	**Authors' judgement**	**Support for judgement**
Random sequence generation (selection bias)	Unclear risk	Quote: “At the baseline, subjects were randomly assigned to two groups” Comment: no further information is given
Allocation concealment (selection bias)	Unclear risk	Quote: “At the baseline, subjects were randomly assigned to two groups” Comment: no further information is given
Blinding (performance bias and detection bias) All outcomes	High risk	Quote: “It was a single blind placebo control study."
Incomplete outcome data (attrition bias) All outcomes	Unclear risk	No relevant information given
Other bias	Low risk	None detected

**Table:** Smith 2009

***Study characteristics***		
Methods	Study design: RCT Country: USA Setting: 12 primary care clinics Recruitment: volunteers from primary care clinics	
Participants	1346 smokers; 56% female; average age 44; average cigarettes per day 20.3	
Interventions	Bupropion. Up‐titrated during week pre‐quitting, 150 mg twice/day for 8 weeks post‐quit Nicotine lozenge. 4 mg lozenge if first cigarette of day smoked > 30 minutes after waking, 2 mg otherwise. 1 lozenge every 1 to 2 hours post‐quit weeks 1 through 6; 1 lozenge every 2 to 4 hours in weeks 7 through 9; 1 lozenge every 4 to 8 hours in weeks 10 through 12 Nicotine patch. 21 mg post‐quit weeks 1 through 4; 14 mg in weeks 5 through 6; 7 mg in weeks 7 through 8 Bupropion and nicotine lozenge. Dosing as above Nicotine patch and nicotine lozenge. Dosing as above Common components: quit‐line counselling (state provided). All participants received initial session, then could elect to receive up to 4 additional calls plus could call for additional support if required.	
Outcomes	Abstinence definition: 7‐day ppa at 6 months. No validation method specified Adverse events: measured for unspecified period	
Funding Source	Majority of funding from National Institutes of Health, National Institute on Drug Abuse, and National Cancer Institute. Medication provided to participants at no cost by GlaxoSmithKline	
Author conflicts of interest	"Dr Smith has received research support from Elan Corporation plc. Dr Jorenby has received research support from Pfizer Inc, Sanofi‐Synthelabo, and Nabi Biopharmaceuticals and has received consulting fees from Nabi Biopharmaceuticals. Dr Fiore has received honoraria from Pfizer Inc and has served as an investigator on research studies at the University of Wisconsin that were funded by Pfizer Inc, Sanofi‐Synthelabo, and Nabi Biopharmaceuticals. In 1998, the University of Wisconsin (UW) appointed Dr Fiore to a named Chair funded by an unrestricted gift to UW from Glaxo Wellcome. Dr Baker has served as an investigator on research projects sponsored by pharmaceutical companies including Sanofi‐Synthelabo, Pfizer Inc, and Nabi Biopharmaceuticals."	
Notes	No control so does not contribute to primary analysis. 4 versus 2 used in Analysis 1.5. 1 versus 3 used in Analysis 1.7.1. 1 versus 2 used in Analysis 1.7.2. 1 versus 5 used in Analysis 1.7.3 (n in 1 divided equally between subgroups to avoid triple counting)	
***Risk of bias***		
**Bias**	**Authors' judgement**	**Support for judgement**
Random sequence generation (selection bias)	Unclear risk	Quote: "Smokers were randomized to the 5 treatment conditions within each clinic with blocking on sex and self‐identified race." Insufficient detail with which to judge
Allocation concealment (selection bias)	Unclear risk	Not specified
Blinding (performance bias and detection bias) All outcomes	High risk	Open‐label
Incomplete outcome data (attrition bias) All outcomes	Low risk	158 individuals who did not pick up study medication at first point not included in analyses; 122 withdrawals and 9 deaths considered to be smoking
Other bias	Low risk	None detected

**Table:** SMK20001

***Study characteristics***		
Methods	Study design: RCT Country: USA Setting: 6 clinical trial centres Recruitment: volunteers for phase II trial	
Participants	286 smokers; 48% female; average age 42; average cigarettes per day not specified	
Interventions	Bupropion 300 mg for 7 weeks and placebo novel therapy Double placebo No information about behavioural support	
Outcomes	Smoking cessation: continuous abstinence at 12 months. Validated by CO ≤ 10 ppm	
Funding Source	GlaxoSmithKline	
Author conflicts of interest	None specified	
Notes	Identified from GSK trials website. Also included a novel cessation aid	
***Risk of bias***		
**Bias**	**Authors' judgement**	**Support for judgement**
Random sequence generation (selection bias)	Unclear risk	Randomisation method not specified
Allocation concealment (selection bias)	Unclear risk	Allocation concealment not described
Blinding (performance bias and detection bias) All outcomes	Unclear risk	Double‐blind but methods not described
Incomplete outcome data (attrition bias) All outcomes	Low risk	34% lost in bupropion, 29% placebo; included as smokers in meta‐analysis
Other bias	Low risk	None detected

**Table:** Sood 2010

***Study characteristics***		
Methods	Study design: RCT Country: USA Setting: community Recruitment: press releases and local advertising	
Participants	118 adult smokers; 82% female; average age 38; average cigarettes per day 20; mean FTND 5.0	
Interventions	St John's wort 900 mg/day (300 mg tablet 3 x day for 12 weeks) St John's wort 1800 mg/day (3 x 300 mg/day tablet first week, 3 x 600 mg/day tablet weeks 2 through 12) Matched placebo on same schedule Common components: 12‐week behavioural intervention using Mayo Clinic ‘Smoke Free and Living It’ manual (type and number of sessions not stated)	
Outcomes	Smoking cessation: prolonged abstinence at 24 weeks (2‐week grace period following quit date). Validated by CO ≤ 8 ppm Adverse events: measured for unspecified period	
Funding Source	National Cancer Institute	
Author conflicts of interest	None specified	
Notes	Groups 1 and 2 combined in meta‐analysis; no significant difference between the two (at 24 weeks, 1/39 abstinent in intervention 1, 2/40 abstinent in intervention 2)	
***Risk of bias***		
**Bias**	**Authors' judgement**	**Support for judgement**
Random sequence generation (selection bias)	Low risk	Generated centrally by Mayo Clinic Division of Biostatistics
Allocation concealment (selection bias)	Unclear risk	Not specified
Blinding (performance bias and detection bias) All outcomes	Unclear risk	Quote: “Blinded” with matched placebo, no further information provided
Incomplete outcome data (attrition bias) All outcomes	Unclear risk	43% dropped out within first 12 weeks, unclear how many dropped out by 24 weeks. Not given by arm
Other bias	Low risk	None detected

**Table:** Sood 2012

***Study characteristics***		
Methods	Study design: RCT Country: USA Setting: clinic Recruitment: community volunteers	
Participants	120 smokers; 47% female; average age 40; average cigarettes per day 20; mean FTND 5.2	
Interventions	SAMe 1600 mg/day (via mouth) for 8 weeks SAMe 800 mg/day. Same schedule as above Placebo. Same schedule as above Common components: behavioural counselling using “Smoke Free and Living It” manual at every clinic visit (approx. 7)	
Outcomes	Smoking cessation: 7‐day ppa at 6 months (prolonged abstinence measured but not reported). Validated by CO ≤ 8 ppm Adverse events: measured for unspecified period	
Funding Source	National Institutes of Health	
Author conflicts of interest	None specified	
Notes	SAMe is a dietary supplement used to treat depression No difference between arms 1 and 2, hence combined in meta‐analysis	
***Risk of bias***		
**Bias**	**Authors' judgement**	**Support for judgement**
Random sequence generation (selection bias)	Unclear risk	Not reported
Allocation concealment (selection bias)	Unclear risk	Not reported
Blinding (performance bias and detection bias) All outcomes	Unclear risk	Quote: "Blinded"; no further detail provided
Incomplete outcome data (attrition bias) All outcomes	Low risk	57% followed up overall, similar rates between groups
Other bias	Low risk	None detected

**Table:** Spring 2007

***Study characteristics***		
Methods	Study design: RCT Country: USA Setting: clinic Recruitment: community volunteers	
Participants	247 smokers; 54% female; average age 44; average cigarettes per day 23	
Interventions	Fluoxetine 60 mg (titrated up over 2 weeks) for 12 weeks Placebo Common components: group behavioural counselling, 9 meetings over 12 weeks	
Outcomes	Smoking cessation: prolonged abstinence at 6 months (starting from 2 weeks after quit date). Validated by CO < 10 ppm, urine cotinine < 20 ng/mL Adverse events: measured for unspecified period	
Funding Source	National Institutes of Health, Veterans Affairs. Medication provided by Eli Lilly and Company	
Author conflicts of interest	None specified	
Notes	First included as Spring 2004 with unpublished data. Full publication reports sustained abstinence	
***Risk of bias***		
**Bias**	**Authors' judgement**	**Support for judgement**
Random sequence generation (selection bias)	Low risk	Quote: "The study pharmacist stratified participants by depression history and used computer‐generated random numbers to assign them to drug or placebo."
Allocation concealment (selection bias)	Unclear risk	Allocated by unblinded pharmacist; method not described
Blinding (performance bias and detection bias) All outcomes	Unclear risk	Quote: "Research staff and participants were blinded to medication status." "Drug assignment was guessed correctly by 59.8% of placebo and 64.6% of fluoxetine participants. Facilitators guessed correctly for 65.3% of placebo and 55.6% of fluoxetine participants."
Incomplete outcome data (attrition bias) All outcomes	Low risk	Withdrawals/lost to follow‐up 40% for fluoxetine, 48% placebo. Authors report similar results from missing assumed smoking and generalised estimating equation (GEE) analyses. All participants included in meta‐analysis
Other bias	Low risk	None detected

**Table:** Stapleton 2013

***Study characteristics***		
Methods	Study design: RCT Country: UK Setting: smoking cessation clinics Recruitment: people attending smoking cessation clinics	
Participants	1071 daily smokers; 53% female; average age 41; average cigarettes per day 20	
Interventions	Bupropion 8 weeks, started prior to TQD (exact period not specified), 150 mg/day for first 6 days, then 300 mg for remainder Bupropion and NRT. Bupropion as above. NRT given as choice of single product, 12 weeks started on TQD, dosage determined on individual basis NRT. As above Common components: 7 weekly behavioural support sessions as per standard service protocol. Mainly group, 60 to 90 minutes each	
Outcomes	Smoking cessation: prolonged abstinence at 6 months. Validated by CO < 10 ppm Adverse events: measured for unspecified period	
Funding Source	Department of Health for England. Study medication provided free of charge by Pfizer UK, GSK UK and Novartis UK	
Author conflicts of interest	None specified	
Notes		
***Risk of bias***		
**Bias**	**Authors' judgement**	**Support for judgement**
Random sequence generation (selection bias)	Low risk	Quote: “Randomization and packaging was organized by an independent statistician at the host site.”
Allocation concealment (selection bias)	Low risk	Quote: “On enrolment, participants selected their envelope from a large batch and signed it before breaking the seal to reveal their allocation.”
Blinding (performance bias and detection bias) All outcomes	High risk	Open‐label, no blinding
Incomplete outcome data (attrition bias) All outcomes	Low risk	61.5% followed up at both 1 month and 6 months, no significant difference between groups. Prolonged abstinence only imputed for 16% of total
Other bias	Low risk	None detected

**Table:** Swan 2003

***Study characteristics***		
Methods	Study design: 2 x 2 factorial RCT Country: USA Setting: a large health system (Group Health Cooperative) in Seattle Recruitment: volunteers from Group Health Co‐op membership	
Participants	1524 smokers; 57% female; average age 45; average cigarettes per day 23	
Interventions	Factorial design crossing 2 drug doses with 2 intensities of behavioural counselling: Bupropion 300 mg/day versus 150 mg/day Free and Clear proactive telephone counselling (4 brief calls), access to quit‐line and S‐H materials versus Zyban Advantage Program (ZAP) tailored S‐H materials, single telephone call after TQD, access to Zyban support line	
Outcomes	Smoking cessation: 7‐day ppa at 12 months. Validation method not specified Adverse events: measured for 13 weeks	
Funding Source	National Cancer Institute	
Author conflicts of interest	None specified	
Notes	Based on published data from 2004 No dose/behavioural treatment interaction at 12 months so arms combined to compare 300 mg versus 150 mg doses. Effects differed at 3 months and 12 months. Effect of higher dose disappeared and additional support aided people to make repeated quit attempts.	
***Risk of bias***		
**Bias**	**Authors' judgement**	**Support for judgement**
Random sequence generation (selection bias)	Low risk	Quote: "Open‐label randomized trial...The computer code for the procedure calculated probabilities of group assignment that were dynamically modified based on the number of members in each group so that final group sizes were equal. No restrictions such as stratification or blocking were used as part of the randomization process."
Allocation concealment (selection bias)	Low risk	Procedure built into study database
Blinding (performance bias and detection bias) All outcomes	High risk	Open‐label
Incomplete outcome data (attrition bias) All outcomes	Low risk	Similar percentage lost to follow‐up across all groups (approx. 15%). Non‐responders treated as smoking
Other bias	Low risk	None detected

**Table:** Swanson 2003

***Study characteristics***		
Methods	Study design: parallel RCT Country: USA Setting: aboard navy ships Recruitment method: convenience sampling; quote: "at the worksites, subjects were recruited by flyers, public announcements, and word of mouth."	
Participants	140 smokers randomised; 7% female, average age 27; average cigarettes per day 19, mean FTND 5.6	
Interventions	Control: counselling only, 9 weeks Nicotine patch, 9 weeks Bupropion, 9 weeks Nicotine patch and bupropion, 9 weeks Common components: "American Cancer Society 'FreshStart' program. This is a 4‐week class (1.5‐hour class per week) stressing behavior modification, identification of smoking triggers, nicotine fading, and relaxation... Following the American Cancer Society 'FreshStart' program, counselling was offered in the form of a support group for 1 hour on weeks 5, 6, 8, 10, and 12."	
Outcomes	Smoking cessation: "Continuous abstinence was defined as the percentage of subjects reporting no smoking since the quit date and having an expired carbon monoxide concentration of less than 10 ppm at sessions 2, 3, and 4", measured for 26 and 52 weeks. Adverse events: measured for 52 weeks, but not reported by subgroup	
Funding Source	Not reported	
Author conflicts of interest	Not reported	
Notes		
***Risk of bias***		
**Bias**	**Authors' judgement**	**Support for judgement**
Random sequence generation (selection bias)	Unclear risk	No information provided
Allocation concealment (selection bias)	Unclear risk	No information provided
Blinding (performance bias and detection bias) All outcomes	High risk	No placebo
Incomplete outcome data (attrition bias) All outcomes	Unclear risk	Less than 50% overall, and less than 20% difference between arms
Other bias	Low risk	None detected

**Table:** Tashkin 2001

***Study characteristics***		
Methods	Study design: RCT Country: USA Setting: multicentre Recruitment: advertisements for volunteers	
Participants	404 smokers with mild to moderate COPD (excludes 7 early dropouts who did not take any study medication); 45% female; average age 53 to 54; average cigarettes per day 28	
Interventions	Bupropion SR 300 mg/day for 12 weeks from 1 week before TQD Placebo Common components: brief face‐to‐face counselling at each clinic visit (weeks 1 to 7, 10, 12), telephone counselling 3 days after TQD	
Outcomes	Smoking cessation: sustained abstinence at 52 weeks (starting from week 4). Validated by CO ≤ 10 ppm at each visit Adverse events: measured for 12 weeks	
Funding Source	GlaxoWellcome Inc.	
Author conflicts of interest	None specified	
Notes	ITT population defined as those taking at least one dose of study medication	
***Risk of bias***		
**Bias**	**Authors' judgement**	**Support for judgement**
Random sequence generation (selection bias)	Low risk	Quote: "Randomised as per code provided by Glaxo Wellcome, using block sizes of four stratified by centre. Within each block of four, two participants were assigned placebo and two bupropion SR. The randomisation codes were kept at the study sites during the trial and we instructed investigators to break the code only for a medical emergency."
Allocation concealment (selection bias)	Low risk	See above
Blinding (performance bias and detection bias) All outcomes	Unclear risk	Double‐blind study, but further detail not provided
Incomplete outcome data (attrition bias) All outcomes	Low risk	64% intervention and 73% control followed up at 6 months. "All participants who withdrew from the study were taken to be smokers thereafter."
Other bias	Low risk	None detected

**Table:** Tidey 2011

***Study characteristics***		
Methods	Study design: factorial trial Country: USA Setting: Providence Veterans Affairs Medical Center and the Brown University Center for Alcohol and Addiction Studies Recruitment method: advertisements posted in the surrounding community and at an outpatient clinic at a local VA medical centre	
Participants	Participants diagnosed with schizophrenia or schizoaffective disorder as confirmed by the Structured Clinical Interview for the Diagnostic and Statistical Manual of Mental Disorders, Fourth Edition, Text Revision (DSM‐IV‐TR) Axis I Disorders 57 participants randomised; 29% female; average age 45.1; average cigarettes per day 27; mean FTND 7.1	
Interventions	Bupropion 150 mg daily for 3 days, then 150 mg twice daily for 3 weeks, starting 1 week prior to TQD Placebo As this was a factorial trial, all participants were randomised to contingency management or none.	
Outcomes	Smoking cessation: 22 days ‐ too short a follow‐up to be considered as part of this review Adverse events: measured for 22 days	
Funding Source	National Institutes of Health (NIH) grant R01‐DA17566 to the first author and a Senior Research Career Scientist Award from the Department of Veterans Affairs to the second author	
Author conflicts of interest	None detailed	
Notes		
***Risk of bias***		
**Bias**	**Authors' judgement**	**Support for judgement**
Random sequence generation (selection bias)	Low risk	Quote: “randomized by coin toss."
Allocation concealment (selection bias)	Unclear risk	No relevant information given
Blinding (performance bias and detection bias) All outcomes	Unclear risk	States "double‐blind", but no further information given
Incomplete outcome data (attrition bias) All outcomes	Low risk	> 94% follow‐up in all groups, with no between‐group differences
Other bias	Low risk	None detected

**Table:** Tonnesen 2003

***Study characteristics***		
Methods	Study design: RCT Country: 8 European countries, Australia, New Zealand Setting: 28 clinical trial centres Recruitment: community volunteers	
Participants	710 smokers; 51% female; average age 42; median cigarettes per day 20	
Interventions	Bupropion SR 300 mg/day for 7 weeks Placebo Common components: brief motivational support at weekly clinic visits and telephone support during follow‐up. 11 clinic visits and 10 phone calls scheduled	
Outcomes	Smoking cessation: prolonged abstinence at 52 weeks (starting from week 4). Validated by CO ≤ 10 ppm Adverse events: measured for 52 weeks	
Funding Source	GlaxoSmithKline	
Author conflicts of interest	S Tonstad has received honoraria from GlaxoSmithKline for lectures on smoking cessation. R Sweet is a former employee of GlaxoSmithKline. A Hider and J Townsend are currently employees of GlaxoSmithKline. For A Hjalmarsson, PI VanSpiegel, P Tonnesen: no conflict of interest was declared	
Notes	First included in 2003 as Tonstad 2001 ITT population defined as those taking at least one dose of study medication; excludes 3 randomised participants	
***Risk of bias***		
**Bias**	**Authors' judgement**	**Support for judgement**
Random sequence generation (selection bias)	Low risk	Quote: "GlaxoSmithKline created a randomization schedule in a 3:1 bupropion: placebo ratio. Each centre received a list with treatment numbers and subjects were consecutively assigned a treatment number at the baseline visit."
Allocation concealment (selection bias)	Low risk	Quote: "GlaxoSmithKline supplied bupropion SR 150 mg and placebo‐to‐match tablets for oral administration as white, film‐coated tablets."
Blinding (performance bias and detection bias) All outcomes	Unclear risk	Double‐blind but methods not described
Incomplete outcome data (attrition bias) All outcomes	Low risk	9% of bupropion SR and 12% placebo were lost to follow‐up
Other bias	Low risk	None detected

**Table:** Tonstad 2003

***Study characteristics***		
Methods	Study design: RCT Country: 10 countries including European countries, Australia, and New Zealand Setting: 28 clinical trial centres Recruitment: volunteers with CVD	
Participants	629 smokers with stable CVD; 23% female; average age 55; average cigarettes per day 25; 49% had history of MI	
Interventions	Bupropion SR 300 mg/day for 7 weeks, begun 1 to 2 weeks before TQD Placebo Common components: brief motivational support at weekly clinic visits and telephone support during follow‐up. 9 clinic visits and 10 phone calls scheduled	
Outcomes	Smoking cessation: prolonged abstinence at 12 months (starting from week 4). Validated by CO ≤ 10 ppm Adverse events: measured for 9 weeks	
Funding Source	GlaxoSmithKline	
Author conflicts of interest	None specified	
Notes	First included in 2003 as McRobbie 2003. ITT population = 626 defined as those taking at least one dose of study medication	
***Risk of bias***		
**Bias**	**Authors' judgement**	**Support for judgement**
Random sequence generation (selection bias)	Unclear risk	Randomisation method not described
Allocation concealment (selection bias)	Unclear risk	Not described
Blinding (performance bias and detection bias) All outcomes	Unclear risk	Double‐blind, but no further detail provided
Incomplete outcome data (attrition bias) All outcomes	Unclear risk	Number missing follow‐up in each group not provided. At 12 months, 38% bupropion and 50% placebo had prematurely discontinued treatment. "Subjects with missing investigator assessments were assumed to be smokers at that visit."
Other bias	Low risk	None detected

**Table:** Urdapilleta‐Herrera 2013

***Study characteristics***		
Methods	Study design: RCT Country: Mexico Setting: not specified Recruitment: not specified	
Participants	94 "chronic smokers" randomised; average age 48; average pack per year 25	
Interventions	Bupropion, no schedule and dose detailed Placebo, no schedule and dose detailed Common components: CBT	
Outcomes	Smoking cessation: at 1 year (no definition of abstinence given). No validation method detailed Adverse events: not detailed whether adverse events were recorded	
Funding Source	None specified	
Author conflicts of interest	None specified	
Notes	Only limited information available as study was only reported as a conference abstract. Outcome data are insufficient to include in meta‐analysis as it is unclear whether percentages reported were calculated using all participants randomised or only those followed up as the denominator. Attempt to contact the authors was unsuccessful. Results are summarised narratively.	
***Risk of bias***		
**Bias**	**Authors' judgement**	**Support for judgement**
Random sequence generation (selection bias)	Unclear risk	No relevant information given
Allocation concealment (selection bias)	Unclear risk	No relevant information given
Blinding (performance bias and detection bias) All outcomes	Unclear risk	Quote: "double‐blind" although no information given regarding who was blinded
Incomplete outcome data (attrition bias) All outcomes	Unclear risk	No relevant information given
Other bias	Low risk	None detected

**Table:** Uyar 2007

***Study characteristics***		
Methods	Study design: RCT Country: Turkey Setting: cessation clinic Recruitment: cessation clinic patients	
Participants	131 smokers; 19% female; average age 36	
Interventions	Bupropion 300 mg for 7 weeks Nicotine patch 21 mg for 6 weeks including tapering Advice and follow‐up only Common components: brief counselling on consequences of smoking with follow‐up for 24 weeks ‐ more than low intensity	
Outcomes	Smoking cessation: abstinence at 24 weeks (definition not specified). Validated by CO < 10 ppm Adverse events: measured for unspecified period	
Funding Source	None specified	
Author conflicts of interest	None specified	
Notes	First included based on abstract. Contributes to bupropion versus control and bupropion versus nicotine patch	
***Risk of bias***		
**Bias**	**Authors' judgement**	**Support for judgement**
Random sequence generation (selection bias)	Unclear risk	Quote: "Randomly allocated"; method not described, unclear why fewer in control condition
Allocation concealment (selection bias)	Unclear risk	Allocation concealment not described
Blinding (performance bias and detection bias) All outcomes	High risk	Open‐label
Incomplete outcome data (attrition bias) All outcomes	Unclear risk	No mention of any losses to follow‐up
Other bias	Low risk	None detected

**Table:** Wagena 2005

***Study characteristics***		
Methods	Study design: RCT Country: the Netherlands Setting: university medical centre Recruitment: community volunteers	
Participants	255 smokers with or at risk of COPD; 51% female; average age 51; average cigarettes per day 23	
Interventions	Bupropion SR 300 mg/day for 12 weeks Nortriptyline 75 mg/day for 12 weeks Placebo bupropion or placebo nortriptyline Common components: individual counselling 10 to 20 minutes at baseline, 1 week and 3 weeks post‐TQD (TQD typically day 11). Telephone support TQD, 2 weeks, 4 weeks, 6 weeks, 8 weeks, 11 weeks	
Outcomes	Smoking cessation: prolonged abstinence at 26 weeks (puff‐free from week 4). Validated by urine cotinine ≤ 60 ng/mL at 4 weeks, 12 weeks and 26 weeks Adverse events: none specified	
Funding Source	Netherlands Asthma Foundation, Netherlands Organization for Health Research and Development. Lundbeck BV provided nortriptyline free of charge	
Author conflicts of interest	None specified	
Notes		
***Risk of bias***		
**Bias**	**Authors' judgement**	**Support for judgement**
Random sequence generation (selection bias)	Low risk	Computer‐generated by pharmacist, stratified by COPD severity, block size 33
Allocation concealment (selection bias)	Low risk	Research staff blinded throughout study
Blinding (performance bias and detection bias) All outcomes	Unclear risk	Double‐blind but "at both time points, participants receiving active drug compared with those receiving placebo were more likely to guess that they had received bupropion SR and nortriptyline treatment (72% vs 43%, P.01; and 62% vs 37%; P=.001; respectively)."
Incomplete outcome data (attrition bias) All outcomes	Low risk	10 (12%) bupropion, 13 (16%) nortriptyline, 12 (13%) lost or withdrawn. All included in ITT analysis
Other bias	Low risk	None detected

**Table:** Weinberger 2008

***Study characteristics***		
Methods	Study design: RCT Country: USA Recruitment method: outpatient mental health clinics Setting: not specified	
Participants	Participants were clinically stable outpatients with Diagnostic and Statistical Manual of Mental Disorders, Fourth Edition (DSM‐IV) diagnoses of bipolar I disorder. 5 participants randomised; 60% female; average age 57; average cigarettes per day 20; mean FTND 6.4	
Interventions	Bupropion. 75 mg for 3 days following quit date, increased to 150 mg for 4 days, then increased to final dose of up to 150 mg twice daily by day 15. Continued for an additional 8 weeks Placebo. Same dose and scheduling as bupropion Common components: weekly sessions of manualised group behavioral therapy	
Outcomes	Smoking cessation: not specified Adverse events: measured for 10 weeks	
Funding Source	NIDA; National Alliance for Research in Schizophrenia and Depression	
Author conflicts of interest	"Dr Weinberger reports receiving grant support from Sepracor, Inc. and the National Alliance for Research on Schizophrenia and Depression (NARSAD). Dr George reports that he received grant support from the National Institute on Drug Abuse (NIDA), NARSAD, The Donaghue Medical Research Foundation, Sanofi‐Aventis, Targacept, and Sepracor. Inc. He is on Advisory Boards and a consultant to Pfizer, Inc. Eli Lilly, Janssen, and Evotec. Dr Chengappa reports that he received grant support from Janssen‐Ortho, Inc, Stanley Medical Research Institute, NIDA, NARSAD. He is on Advisory Boards for Astra Zeneca and Lilly."	
Notes	Only safety data available in letter to editor, and included in analysis.	
***Risk of bias***		
**Bias**	**Authors' judgement**	**Support for judgement**
Random sequence generation (selection bias)	Unclear risk	No relevant information given
Allocation concealment (selection bias)	Unclear risk	No relevant information given
Blinding (performance bias and detection bias) All outcomes	Unclear risk	Paper states that the trial was placebo‐controlled, but no further information given
Incomplete outcome data (attrition bias) All outcomes	Low risk	Dropout rates were as follows: 1/3 in placebo; 1/2 in bupropion. Therefore loss to follow‐up was less than 50% and similar between groups.
Other bias	Low risk	None detected

**Table:** Weinberger 2010

***Study characteristics***		
Methods	Study design: RCT Country: USA Setting: clinics Recruitment: community volunteers	
Participants	101 smokers (excludes 2 taking no medication); 50% female; average age 47; average cigarettes per day 22	
Interventions	Selegiline 10 mg/day for 9 weeks (5 mg/day in week 1 and week 9) Placebo Common components: brief weekly counselling	
Outcomes	Smoking cessation: 7‐day ppa at 6 months. Validated by CO and urinary cotinine Adverse events: measured for 10 weeks	
Funding Source	National Institute of Drug Abuse, Veteran's Administration, Women's Health Research at Yale, NIH, University of Toronto	
Author conflicts of interest	None specified	
Notes		
***Risk of bias***		
**Bias**	**Authors' judgement**	**Support for judgement**
Random sequence generation (selection bias)	Unclear risk	Randomisation method not described
Allocation concealment (selection bias)	Unclear risk	Not described
Blinding (performance bias and detection bias) All outcomes	Low risk	Quote: "Both participants and research staff were blinded to study medication assignment". Comment: assessments of staff and participants suggest blinding was adequate
Incomplete outcome data (attrition bias) All outcomes	Unclear risk	27.5% selegiline, 42% placebo lost at 6 months. Including all participants is less conservative
Other bias	Low risk	None detected

**Table:** Weiner 2012

***Study characteristics***		
Methods	Study design: RCT Country: USA Setting: Maryland Psychiatric Research Center Recruitment method: clinically stable outpatients from the Maryland Psychiatric Research Center volunteered to participate	
Participants	Participants had a Diagnostic and Statistical Manual of Mental Disorders, Fourth Edition (DSM‐IV) diagnosis of schizophrenia or schizoaffective disorder made through a best estimate diagnostic approach. 46 participants randomised; 19.6% female; average age 49; average cigarettes per day 44.0; mean FTND 5.8	
Interventions	Bupropion 150 mg daily for 3 days, then 150 mg twice daily from day 4 onwards, through 12 weeks Placebo, dose and scheduling the same as bupropion All participants had a 9‐week group support programme led by staff trained in the education model of the American Cancer Society FreshStart Program modified for people with schizophrenia. Each session was structured and incorporated relation exercises with practice “homework”. The first group sessions were designed to increase awareness of specific smoking habits and to develop a ‘Quit Plan’. A Quit Day Ceremony was held at the fifth group session. Subsequent sessions focused on reworking the Quit Plan. Later groups focused on strategies for participants minimising weight gain, managing high risk situations, and imagining themselves as non‐smokers.	
Outcomes	Smoking cessation: 14 weeks ‐ too short a follow‐up for this outcome to be considered as part of this review Adverse events: measured for 14 weeks	
Funding Source	Veterans Affairs Capitol Network (VISN 5) Mental Illness Research, Education, and Clinical Center. National Institute of Mental Health Grant (MH068580‐01), Advance Center for Intervention Services Research	
Author conflicts of interest	"Ms Ball has served as a consultant to ePharmaSolutions and Pfizer; Dr Gold has served as a consultant to Merck, AstraZeneca, Solvay, Pfizer, and GlaxoSmithKline. Dr Evins has served as a consultant to Pfizer, Boehringer, and Schering Plough and has received grant/research support from GlaxoSmithKline and Pfizer. Dr Buchanan has served as a consultant to Abbott and ClaxoSmithKline; has received grant/research support from Novartis and Janssen; has served on advisory boards for AstraZeneca, Wyeth, Schering Plough, Solvay and Pfizer, and has received other material or financial support from Bristol‐Myers Squibb, Otsuka, Pfizer and Cephalon. Drs Weiner and McMahon and Ms Buchholz report no financial or other relationship relevant to the subject of this article."	
Notes		
***Risk of bias***		
**Bias**	**Authors' judgement**	**Support for judgement**
Random sequence generation (selection bias)	Unclear risk	Quote: “Random assignments made by the statistician." Comment: no further information given
Allocation concealment (selection bias)	Unclear risk	Quote: “Random assignments made by the statistician." Comment: no further information given
Blinding (performance bias and detection bias) All outcomes	Unclear risk	Quote: “double‐blind, placebo‐controlled clinical trial." Comment: no further information given
Incomplete outcome data (attrition bias) All outcomes	Low risk	Dropout rates are as follows: 8/24 (33.3%) in the bupropion group; 6/22 (27.3%) in the placebo group. Therefore overall dropout was less than 50% and similar between groups.
Other bias	Unclear risk	Quote: “While the target completion number was 40 there was insufficient study drug available to meet this goal." It is unclear how this was dealt with and whether it is accounted for in the dropouts reported in the flow diagram. However, loss to follow‐up was similar between arms.

**Table:** White 2005

***Study characteristics***		
Methods	Study design: RCT Country: Canada Setting: university Recruitment method: local media	
Participants	36 participants randomised; 61.1% female; average age 41.9; average cigarettes per day 24.0; mean FTND 7.2	
Interventions	Bupropion 150 mg on days 1 to 3, then 150 mg twice daily for the remainder of the 6‐week study Gabapentin started at 300 mg daily, with titration to 1800 mg daily by day 6 All participants each week received 15‐minute one‐to‐one smoking cessation counselling with a study investigator, using the Mayo Clinic workbook "Smoke‐Free and Living It" for a total of 1 hour and 30 minutes	
Outcomes	Smoking cessation: 6 weeks ‐ too short a follow‐up for this outcome to be considered as part of this review Adverse events: measured for 6 weeks	
Funding Source	Calgary Centre for Advancement of Health. Gabapentin (Neurontin) samples were donated through an informal arrangement with a local representative of Pfizer Canada Inc.	
Author conflicts of interest	None detailed	
Notes		
***Risk of bias***		
**Bias**	**Authors' judgement**	**Support for judgement**
Random sequence generation (selection bias)	Unclear risk	Quote: “we conducted a randomized, open‐label pilot trial." Comment: no further information given
Allocation concealment (selection bias)	Unclear risk	Quote: “we conducted a randomized, open‐label pilot trial." Comment: no further information given
Blinding (performance bias and detection bias) All outcomes	High risk	Quote: “we conducted a randomized, open‐label pilot trial” Comment: open‐label
Incomplete outcome data (attrition bias) All outcomes	Low risk	Dropout rates are as follows: 9/19 (47.4%) in the bupropion group; 6/17 (35.3%) in the gabapentin group. Therefore overall attrition was less than 50% and similar between arms.
Other bias	Low risk	None detected

**Table:** Wittchen 2011

***Study characteristics***		
Methods	Study design: RCT Country: Germany Setting: 167 primary care clinics Recruitment: patients at participating primary care clinics	
Participants	467 "current regular smokers"; 52% female; average age 43; average cigarettes per day 20	
Interventions	CBT 4 to 5 one‐on‐one counselling sessions for 20 to 30 minutes CBT and bupropion SR. CBT as above. Bupropion SR (9 to 12 weeks, 150 mg; 1/day for first 6 days; 2/day thereafter) CBT and NRT. CBT as above. NRT for 9 to 12 weeks, patient's choice of patch (7 mg to 52.5 mg), gum (2 or 4 mg) or spray (10 mg/mL) Minimal intervention (not used in review)	
Outcomes	Smoking cessation: abstinence at 12 months (from EOT). Validation method not specified Adverse events: measured for 12 weeks	
Funding Source	Participants covered all costs for pharmaceutical treatments. Sponsored by the Federal Ministry of Education and Research; additional support provided by GlaxoSmithKline GmbH & Co and Pharmacia GmbH	
Author conflicts of interest	None specified	
Notes	3 versus 2 included in primary analyses. 2 versus 4 included in Analysis 1.7 comparison of NRT with bupropion. 1 not used as results versus bupropion would be confounded with CBT	
***Risk of bias***		
**Bias**	**Authors' judgement**	**Support for judgement**
Random sequence generation (selection bias)	Unclear risk	Quote: "Generated by the study center"; used to put 4 different coloured questionnaires in random order
Allocation concealment (selection bias)	High risk	Quote: "questionnaires were distributed consecutively to all attending patients on the target days by nurses. Thus, the assignment of patients was entirely dependent on the consecutive attendance of patients and the random assignment of a color. Doctors were not allowed to interfere with this study procedure." But numbers allocated to groups very uneven and discussion states: "Random checks of this procedure [randomisation] and quality assurance tests by study monitors revealed that in some cases in the latter part of the study treatment was based on patient and physician preferences." Comment: therefore no concealment
Blinding (performance bias and detection bias) All outcomes	High risk	Neither participants nor providers were blind
Incomplete outcome data (attrition bias) All outcomes	Low risk	Similar number of dropouts between groups; participants lost to follow‐up considered smokers for meta‐analysis
Other bias	Low risk	None detected

**Table:** Zellweger 2005

***Study characteristics***		
Methods	Study design: RCT Countries: 12 European countries Setting: 26 clinical trial centres Recruitment: volunteers, healthcare professionals (qualified practising physician or nurse)	
Participants	667 smokers (excludes 1 centre enrolling 20 people, and 3 people who took no medication); 64% female; average age 40; average cigarettes per day 23; 32% doctor, 68% nurse	
Interventions	Bupropion SR. 300 mg/day for 7 weeks Placebo Common components: brief (10‐ to 15‐minute) motivational support at weekly clinic visits and telephone support one day before TQD, 3 days after TQD, monthly during follow‐up	
Outcomes	Smoking cessation. Prolonged abstinence at 52 weeks (starting from week 4). Validated by CO ≤ 10 ppm	
Funding Source	GlaxoSmithKline	
Author conflicts of interest	None specified	
Notes	Continuous abstinence rates and information on adverse events from GlaxoSmithKline data. One centre excluded	
***Risk of bias***		
**Bias**	**Authors' judgement**	**Support for judgement**
Random sequence generation (selection bias)	Unclear risk	Randomisation method not described
Allocation concealment (selection bias)	Unclear risk	Not described
Blinding (performance bias and detection bias) All outcomes	Unclear risk	Double‐blind but further detail not provided
Incomplete outcome data (attrition bias) All outcomes	Unclear risk	Number lost to follow‐up not stated. Participants with missing assessments or dropouts considered to be smoking
Other bias	Low risk	None detected

**Table:** Zhang 2022

***Study characteristics***		
Methods	Study design: RCT Country: Canada Recruitment method (quote): "The primary method of recruitment was by word of mouth and Facebook advertisement. Interested participants were directed to the study website, where they could indicate their consent for participation and complete eligibility questionnaires"	
Participants	2461 smokers; 56% female, average age 46.5, 46.9% of bupropion group and 44.9% of varenicline group smoked 11 to 20 cigarettes/day	
Interventions	Bupropion 150 mg once daily for first 3 days, then twice daily for the remainder of 12 weeks. Starting 7 days prior to TQD Varenicline 0.5 mg once daily for first 3 days, then 0.5 mg twice daily for next 4 days, then 1 mg twice daily for the remainder of 12 weeks. Starting 7 days prior to TQD Common components: weekly motivational emails	
Outcomes	Smoking cessation: point prevalence measured at 52 weeks. Not biochemically verified. Adverse events: measured at 12 weeks	
Funding Source	Quote: "This research was funded by Global Research Awards for Nicotine Dependence (GRAND), a peer‐reviewed research grant competition funded by Pfizer Pharmaceuticals [Zawertailo (GRAND2012) WS2391913]. The study’s funder had no role in study design, data collection, data analysis, data interpretation, or writing of the report, and had no decision to submit the article for publication. Dr. Le Foll is supported by a clinician‐scientist award from the Department of Family and Community Medicine and by the Addiction Psychiatry Chair of the Department of Psychiatry of University of Toronto. Dr. Selby is sup‐ ported by a clinician‐scientist award from the Department of Family and Community Medicine and CAMH."	
Author conflicts of interest	Quote: "All authors have completed the ICMJE uniform disclosure form at www.icmje.org/coi_disclosure.pdf and declare: no support from any organization for the submitted work; PS reports receiving funding and/ or honoraria from Pfizer Inc./Canada, Shoppers Drug Mart, Bhasin Consulting Fund Inc., Patient‐Centered Outcomes Research Institute, ABBVie, and Bristol‐Myers Squibb; BLF and LZ both receive support from Pfizer Global Research Awards in Nicotine Dependence (GRAND) Award Program; there are no other relationships or activities that could appear to have influenced the submitted work. BLF also reports grants from Brainsway, grants from Bioprojet, grants from Alkermes, grants from Canopy, grants from ACS, non‐financial support from Aurora, outside the submitted work."	
Notes		
***Risk of bias***		
**Bias**	**Authors' judgement**	**Support for judgement**
Random sequence generation (selection bias)	Low risk	Study quote: "Participants were randomly assigned to one of two medication arms (varenicline or bupropion) using permuted‐block randomization in a 1:1 ratio in blocks of 100." Protocol quote: "The randomization process will be computerized."
Allocation concealment (selection bias)	Low risk	Protocol quote: "The randomization process will be computerized."
Blinding (performance bias and detection bias) All outcomes	Low risk	Study quote: "Participants were not blinded to treatment since their health care provider signing the prescription form was required to know which drug was being prescribed to their patient." However, both study groups received active smoking cessation pharmacotherapy treatment. The abstinence outcome was not biochemically verified; however, study arms received the same behavioural support.
Incomplete outcome data (attrition bias) All outcomes	Low risk	The overall number of participants lost at the 52‐week follow up was greater than 50% in each condition. However, a complete case sensitivity analysis (section 3.5.2) did not change findings at the 52‐week follow up (i.e. no difference between conditions).
Other bias	Low risk	None detected

**Table:** Zincir 2013

***Study characteristics***		
Methods	Study design: RCT Country: Turkey Setting: outpatient smoking cessation clinic in a hospital Recruitment method: patients who presented at the smoking cessation outpatient clinic were included in the study on a voluntary basis	
Participants	300 participants randomised; average age: 45.8 in those who stopped smoking and 40.8 in those who continued smoking; average boxes of cigarettes per year: 23.62 in those who stopped smoking and 23.26 in those who continued smoking; mean FTND: 5.9 in those who stopped smoking and 6.7 in those who continued smoking	
Interventions	Bupropion 150 mg/day, started a week before the quit day and continued from days 1 to 3, raised to 300 mg daily on day 4, with this dose maintained until the end of week 12 Varenicline 0.5 mg daily, raised to 1 mg daily at day 4, then to 2 mg daily at day 8, with this dose maintained until the end of week 12 Nicotine replacement therapy. Administered using either a nicotine patch or nicotine gum, or a combination of both. Nicotine patches were used in their three forms containing 21 mg, 14 mg and 7 mg of nicotine, and in cases of excessive nicotine craving, 2 mg nicotine gum was used. For each dose of nicotine patches, 4 weeks of administration in decreasing doses was recommended. The nicotine gum was started between 12 and 24 doses (2 mg) a day and gradually decreased.	
Outcomes	Smoking cessation: definition not specified; measured at 24 to 28 weeks from study start Adverse events: measured for unspecified period	
Funding Source	None specified	
Author conflicts of interest	None detailed	
Notes		
***Risk of bias***		
**Bias**	**Authors' judgement**	**Support for judgement**
Random sequence generation (selection bias)	Unclear risk	Quote: “…they were randomized to the pharmacological therapy groups” Comment: no further information given
Allocation concealment (selection bias)	Unclear risk	Quote: “…they were randomized to the pharmacological therapy groups" Comment: no further information given
Blinding (performance bias and detection bias) All outcomes	High risk	Quote: “This was a naturalistic clinical follow‐up study." Comment: those involved in the study were therefore unblinded
Incomplete outcome data (attrition bias) All outcomes	Unclear risk	300 participants were randomised and 251 completed the study. Therefore 49/300 (16.3%) were lost to follow‐up overall. However, it is impossible to establish the number lost to follow‐up by group.
Other bias	High risk	Quote: “no adverse event was reported during the study”. This is highly unlikely to be correct when considering standard definitions of adverse events. There is no explanation of how adverse events were assessed in this study. In addition, the wording of the paper makes the final follow‐up slightly unclear. After discussion, we judged final follow‐up to be 24 to 28 weeks from study start, although quit rates were higher than would be expected at this time point.

**AE:** adverse event; **CBGT:** cognitive behavioral group therapy; **CBT:** cognitive behavioural therapy; **CES‐D:** Center for Epidemiologic Studies Depression Scale; **CO:** carbon monoxide (in exhaled breath); **COPD:** chronic obstructive pulmonary disease; **CVD:** cardiovascular disease; **EOT:** end of treatment; **FTND:** Fagerstrom Test for Nicotine Dependence; **FTQ:** Fagerstrom Tolerance Questionnaire; **ITT:** intention‐to‐treat; **MDD:** major depressive disorder; **mins:** minutes; **NRT:** nicotine replacement therapy; **ppa:** point prevalence abstinence; **ppm:** parts per million; **RCT:** randomised controlled trial; **RP:** relapse prevention; **Rx:** treatment; **SAE:** serious adverse event; **SAMe:** S‐adenosyl‐L‐methionine; **S‐H:** self‐help; **SR:** sustained release; **TQD:** target quit date; **VAMC:** Veterans Affairs Medical Center

### Characteristics of excluded studies [ordered by study ID]

**Table:**

Study	Reason for exclusion
Akbarpour 2010	Bupropion ‐ short follow‐up
Aryanpur 2016	Arms not matched ‐ different behavioural interventions in each
Banham 2010	Not an RCT ‐ review of smoking cessation treatment for people with severe mental illness
Becker 2003	St John's wort ‐ short follow‐up (1 month)
Berlin 2005	Befloxatone (reversible monoamine oxidase‐B inhibitor) ‐ data not published, treatment reported to have had no effect on abstinence rates
Bloch 2010	Bupropion ‐ trial in people with schizophrenia, short follow‐up and cessation not reported
Bowen 1991	Tryptophan ‐ short follow‐up Tryptophan 50 mg/kg/day, with high carbohydrate low protein diet (7/1 ratio), versus placebo and low carbohydrate high protein diet (1/1 ratio) for two weeks
Brauer 2000	Selegiline ‐ only preliminary short‐term results available. Six‐month follow‐up planned
Breitling 2008	Trial of practitioner education and financial incentives, or cessation drug costs reimbursement
Brody 2013	Ineligible outcomes ‐ less than six months' follow‐up and no safety data reported
Carrão 2007	Sertraline ‐ combined with buspirone so effect of sertraline could not be isolated
Chan 2005	Bupropion ‐ case control study in pregnant women
Chandrashekar 2015	Short‐term follow‐up and no safety assessment
ChiCTR1900020676	Ineligible comparator
Christenhusz 2012	Not randomised to treatments, only treatment strategies
Cornelius 1997	Fluoxetine ‐ cessation not an outcome. Fluoxetine reduced the amount smoked by depressed alcoholic smokers
Cornelius 1999	Fluoxetine ‐ short‐term outcome in a study of depressed alcoholic participants not attempting to quit
Covey 2007	Previously included. Relapse prevention study. See Livingstone‐Banks 2019
Croghan 2007	Previously included. Relapse prevention study. See Livingstone‐Banks 2019
Cropsey 2015	Randomisation to treatment strategy, not actual treatment
Dalack 1995	Fluoxetine ‐ refers to but does not report on a cessation study
Dale 2002	Bupropion ‐ used for smokeless tobacco cessation, not smoking cessation
Dale 2007	Bupropion ‐ for smokeless tobacco cessation, see Ebbert 2011
Edwards 1989	Doxepin ‐ short follow‐up (2 months)
EUCTR2005‐006189‐32‐AT	Arms not matched
Fatemi 2005	Bupropion ‐ short‐term cross‐over trial
Frederick 1997	Venlafaxine ‐ short follow‐up (8 weeks)
Gawin 1989	Buspirone ‐ open trial
Ghorbani Behnam 2019	Ineligible comparator
Gifford 2011	Bupropion ‐ test of behavioural therapy, all participants received bupropion
Glover 2002	Bupropion ‐ used for smokeless tobacco cessation, not smoking cessation
Gold 2002	Bupropion ‐ non‐random assignment, participant preference
Grandi 2011	Bupropion ‐ not an RCT, review of bupropion use in patients with CVD
Grassi 2009	Not an RCT, pre‐post study of influence of smoking ban on people's selection of smoking cessation treatment
Hall 2009	Bupropion ‐ all participants received bupropion for quitting, test of extended CBT or NRT
Hall 2011	Previously included. Relapse prevention study. See Livingstone‐Banks 2019
Hatsukami 2004	Previously included. Harm reduction study. See Lindson‐Hawley 2016
Hawk 2008	Bupropion ‐ short follow‐up (12 weeks). Compares 1 week to 4 week pre‐quit use
Hawk 2015	Interventions not matched ‐ same intervention post‐quit date
Hays 2001	Previously included. Relapse prevention study. See Livingstone‐Banks 2019
Hays 2009	Previously included. Relapse prevention study. See Livingstone‐Banks 2019
Hitsman 1999	Fluoxetine ‐ the majority of participants in this study were also part of the multicentre trial reported in Niaura 2002
Houtsmuller 2002	Selegiline ‐ short‐term laboratory study
Hurt 2003	Previously included. Relapse prevention study. See Livingstone‐Banks 2019
Hussain 2010	Bupropion ‐ short follow‐up, trial in unmotivated smokers
Ionescu 2008	Sertraline and buspirone ‐ effect of antidepressant confounded with that of anxiolytic
Isgro 2015	Topiramate not an antidepressant
Jacobs 1971	Imipramine ‐ short follow‐up. Outcome was reduction in smoking to less than 10% of baseline
Kalman 2004	Bupropion ‐ short follow‐up (12 weeks)
Khunrong 2016	Ineligible outcomes
Killen 2006	Previously included. Relapse prevention study. See Livingstone‐Banks 2019
Kotz 2009	Nortriptyline ‐ pharmacotherapy was confounded with additional counselling from nurse (control group 1), compared to usual care
Kras 2010	St John's wort ‐ short follow‐up
Lawvere 2006	St John's wort ‐ uncontrolled study
Li 2009	Bupropion ‐ short follow‐up
Li 2019	Ineligible comparator
Miller 2003	Bupropion ‐ short follow‐up (8 weeks)
Monuteaux 2007	Bupropion ‐ participants were adolescent non‐smokers, not for cessation
Mooney 2008	Bupropion ‐ short follow‐up, bupropion for opioid and tobacco dependence
Mooney 2016	Bupropion same in both arms
Naranjo 1990	Fluoxetine ‐ study of short‐term smoking behaviour
NCT00032084	Trial terminated before completion
NCT00119210	Trial terminated before completion
NCT00136747	Smoking cessation not measured
NCT00136786	Smoking cessation not measured
NCT00158171	Cessation not measured ‐ harm reduction study
NCT00248118	Bupropion ‐ trial was terminated prior to completion
NCT00320697	Pharmacotherapies not matched
NCT00390923	Selegiline ‐ study terminated early due to lack of efficacy, results available at 9 weeks only
NCT00484692	Bupropion ‐ used as an active control to a psychosocial intervention, cannot estimate pharmacotherapy effect
NCT00580853	Does not measure smoking cessation ‐ ability to resist smoking
NCT00670904	No randomisation ‐ participants chose their medication
NCT00936299	Bupropion ‐ no abstinence outcome reported and follow‐up only 16 weeks
NCT01850589	Behavioural intervention and pharmacotherapy is different between arms
NCT01965405	All participants in all arms receive the same bupropion treatment
NCT02736474	Both naltrexone and bupropion given together in same arm
NCT03471767	Bupropion given in both arms
NCT03920319	Ineligible outcomes
Neumann 2000	Bupropion ‐ smokers randomised to 1 or 2 months of medication (300 mg/day). 91/165 randomised were not included in the analysis, including some 1‐month group participants who requested further medication.
Neumann 2002	Bupropion ‐ short‐term follow‐up. Comparison of 300 mg and 150 mg doses
Niederhofer 2004	Participants were required to be abstinent for at least five days prior to enrolment to trial
Olmstead 1999	Bupropion ‐ all participants received bupropion. Short‐term follow‐up
Paluck 2006	Bupropion ‐ uncontrolled prospective observational study
Pomerleau 1991	Fluoxetine ‐ no cessation data reported
Raynor 2005	Bupropion ‐ short (90‐day) follow‐up. Substudy within a larger trial with long‐term follow‐up, not yet published
Robinson 1991	Buspirone ‐ case series
Ruehle 2021	Inadequate follow‐up
Sanchez 2019	Ineligible intervention
Schiavon 2018	Ineligible comparator
Sellers 1987	Zimelidine or citalopram (SSRIs) ‐ placebo‐controlled cross‐over design study of smoking behaviour and alcohol use in non‐depressed heavy drinkers
Sherman 2008	Bupropion ‐ trial of NRT as adjunct to bupropion
Shiffman 2000	Bupropion ‐ placebo‐controlled short‐term study of effects on craving and withdrawal in participants not wanting to quit smoking permanently
Shoptaw 2008	Bupropion ‐ tested for methamphetamine dependence. Reduction in smoking was a secondary outcome. Only 48/73 participants smoked, quitting not reported.
Sittipunt 2007	Nortriptyline ‐ only 3‐month follow‐up
Spring 1995	Fluoxetine ‐ 6‐month cessation not reported. Primarily a study of post‐cessation weight gain
Stein 1993	Fluoxetine ‐ does not report outcomes from a double‐blind study
Steinberg 2009	Bupropion ‐ confounded with nicotine inhaler and treatment duration in comparison with nicotine patch alone
Strayer 2004	Bupropion ‐ all participants prescribed bupropion. Test of behavioural interventions, not bupropion. Adverse event data from author used
TCTR20190506002	Ineligible comparator
Tidey 2009	Bupropion ‐ laboratory study, outcomes included urge to smoke, not cessation
Toll 2007	Bupropion ‐ all participants had same pharmacotherapy
Weiner 2001	Bupropion ‐ no control group
Winhusen 2012	Bupropion confounded by other agents
Zernig 2008	Bupropion ‐ used as an active control to a psychosocial intervention, cannot estimate pharmacotherapy effect
ZYB30011	Bupropion ‐ follow‐up only to end of treatment (7 weeks)

**CBT:** cognitive behavioural therapy; **CVD:** cardiovascular disease; **NRT:** nicotine replacement therapy; **RCT:** randomised controlled trial; **SSRI:** selective serotonin reuptake inhibitor

### Characteristics of ongoing studies [ordered by study ID]

**Table:** NCT03326128

Study name	High dose bupropion for smoking cessation
Methods	Triple‐blind randomised trial
Participants	300 heavy smokers who also experience psychiatric symptoms
Interventions	Bupropion 300 mg 4 weeks before and 4 weeks after TQD Bupropion 450 mg 4 weeks before and 4 weeks after TQD Common components: standard smoking cessation counselling for 8 weeks
Outcomes	Smoking cessation: point prevalence abstinence at 26 weeks post‐quit date. Validated by self‐report Self‐report of smoking status
Starting date	May 2019
Contact information	Lauren Whitted, 323‐442‐1197, lwhitted@usc.edu
Notes	At time of 2023 update, not yet recruiting

**Table:** NCT03342027

Study name	Smoking cessation interventions for people living with HIV in Nairobi, Kenya
Methods	2 x 2 factorial, double‐blind randomised controlled trial
Participants	300 participants living with HIV, who smoke and who are receiving care in a methadone maintenance programme, will be randomised
Interventions	Bupropion and Positively Smoke Free (an 8‐session tailored behavioural intervention for smokers living with HIV) Bupropion and standard of care (brief advice to quit) Placebo and Positively Smoke Free Placebo and standard of care
Outcomes	Smoking cessation: 7‐day point prevalence abstinence at 36 weeks. Validated by expired CO < 7 ppm
Starting date	20 August 2019
Contact information	Wendy Potts, (410) 706‐2490, wpotts@som.umaryland.edu
Notes	At time of 2023 update, study recruiting

**Table:** NCT04604509

Study name	Nicotine replacement therapy, counselling, varenicline, and bupropion for smoking cessation, the PISCES I Trial
Methods	Parallel, open‐label, randomised controlled trial
Participants	2010 participants estimated who smoke 5 or more cigarettes/day and who are motivated to quit
Interventions	Varenicline and counselling NRT and counselling Varenicline or NRT and counselling Higher dose varenicline or NRT and counselling Varenicline or NRT, bupropion, counselling
Outcomes	Primary End of treatment 7‐day point prevalence, measured up to 6 months Expired carbon monoxide value, measured up to 6 months Abstinence at 12 weeks Secondary Days to relapse, measured up to 6 months
Starting date	October 2020
Contact information	Paul Cinciripini, pcinciri@mdanderson.org
Notes	At time of 2023 update, still recruiting

**Table:** NCT05205811

Study name	The effects of combination zonisamide and bupropion on switching to an electronic cigarette
Methods	Parallel, double‐blind, randomised controlled trial
Participants	180 participants estimated who smoke at least 10 commercially available cigarettes per day and who are interested in switching to an e‐cigarette
Interventions	Combination zonisamide and bupropion with e‐cigarette Bupropion with e‐cigarette Placebo with e‐cigarette
Outcomes	Complete switching from combustible cigarettes to JUUL e‐cigarette as measured by exhaled carbon monoxide (CO), measured after 8 weeks Complete switching from combustible cigarettes to JUUL e‐cigarette as measured by change in total urinary 4‐(methylnitrosamino)‐1‐(3‐pyridyl)‐1‐butanol (NNAL), measured at baseline, week 8 and week 12 Complete switching from combustible cigarettes to JUUL e‐cigarette as measured by change in self‐report of daily cigarette and e‐cigarette use, measured daily from weeks 2 to 12
Starting date	January 2022
Contact information	Derek Mercedes, derek.mercedes@roseresearchcenter.com
Notes	At time of 2023 update, still recruiting

**CO:** carbon monoxide (in exhaled breath); **NRT:** nicotine replacement therapy; **ppm:** parts per million; **TQD:** target quit date

## Differences between protocol and review

We made the changes below for the 2023 update.

- For pragmatic reasons, we only generated funnel plots for the most clinically relevant comparisons and outcomes (i.e. those included in our summary of findings tables).
- As piloted in the previous version of the review, we combined analyses of harms and tolerability for bupropion only across the relevant comparisons, to increase the power of our analyses. We continued to subgroup according to the relevant comparison.
- We screened previously excluded studies for a complementary network meta‐analysis (Lindson 2022), and through duplicate consensus, included some studies that were formerly excluded from previous updates of this review.
- We discovered that two references to the same study had been included as separate studies in the previous update of the review. In that version of the review, this study did not contribute to meta‐analyses and therefore did not threaten their integrity. We have combined the references in this version of the review under the study ID Weinberger 2008.
